# Nematicidal spore-forming Bacilli share similar virulence factors and mechanisms

**DOI:** 10.1038/srep31341

**Published:** 2016-08-19

**Authors:** Ziqiang Zheng, Jinshui Zheng, Zhengming Zhang, Donghai Peng, Ming Sun

**Affiliations:** 1 State Key Laboratory of Agricultural Microbiology, College of Life Science and Technology, Huazhong Agricultural University, Wuhan 430070, China

## Abstract

In the soil environment, Bacilli can affect nematode development, fecundity and survival. However, although many *Bacillus* species can kill nematodes, the virulence mechanisms Bacilli utilize remain unknown. In this study, we collected 120 strains comprising 30 species across the Bacillaceae and Paenibacillaceae families of the Bacillales order and measured their nematicidal activities *in vitro*. Comparison of these strains’ nematicidal capacities revealed that nine species, including *Bacillus thuringiensis, B. cereus, B. subtilis, B. pumilus, B. firmus, B. toyonensis, Lysinibacillus sphaericus, Brevibacillus laterosporus* and *B. brevis*, were highly nematicidal, the first of which showed the highest activity. Genome sequencing and analysis identified many potential virulence factors, which grouped into five types. At least four possible mechanisms were deduced on the basis of the combination of these factors and the bacterial nematicidal activity, including a pore-forming mechanism of crystal proteins, an inhibition-like mechanism of thuringiensin and a degradation mechanism of proteases and/or chitinases. Our results demonstrate that 120 spore-forming Bacilli across different families share virulence factors that may contribute to their nematicidal capacity.

Bacilli and nematodes coexist in environments, notably in soil. In the soil, Bacilli can affect nematode development, fecundity and survival^1^. Some interactions between Bacilli and nematodes may be beneficial for nematodes. For example, *Bacillus subtilis* GS67 can protect *Caenorhabditis elegans* from Gram-positive pathogens through fengycin-mediated microbial antagonism^2^. Furthermore, Bacilli can provide *C. elegans* with nitric oxide (NO)^3^. Many *Bacillus* species can kill nematodes, such as *C. elegans* which prey on soil bacteria, including *Bacillus*^4^. Rae *et al*. found that 20 out of 768 *Bacillus* strains isolated from soil, consisting of *B. cereus, B. weihenstephanensis, B. mycoides* and other *Bacillus* sp. showed nematicidal activity^1^. The study of the relationships between Bacilli and nematodes is critical for understanding microbial pathogenesis in nematodes. However, although many *Bacillus* species can kill nematodes, the virulence mechanisms Bacilli utilize remain unknown. We used spore-forming Bacilli and free-living *C. elegans* to investigate the virulence mechanisms of Bacilli to nematodes. We focus on their roles in nematicide, but many of these mechanisms are general virulence factors with roles in insect or vertebrate pathogenesis as well.

Spore-forming Bacilli, such as *B. subtilis*, are important members of the Bacilli, the largest class in the phylum Firmicutes, which contains two orders, Bacillales and Lactobacillales. According to the list of prokaryotic names with standing in nomenclature (LPSN, http://www.bacterio.net/index.html) sponsored by Bergey’s Manual of Systematics of Archaea and Bacteria (BMSAB), a total of 729 species of Bacilli can form endospores. These species group into seven families, including Alicyclobacillaceae, Bacillaceae, Paenibacillaceae, Pasteuriaceae, Planococcaceae, Sporolactobacillaceae and Staphylococcaceae. Up to 47 species in the spore-forming Bacillaceae, Paenibacillaceae and Pasteuriaceae family of Bacillales order have nematicidal activity, according to the literature (Fig. S1 and Table S1). Although the nematicidal capacities of these species have been documented in recent decades, and some nematicidal virulence factors have been reported, the nematicidal mechanisms of spore-forming Bacilli are unclear, with some exceptions. First, the pore-forming mechanism of crystal proteins (Cry), such as Cry5B from *B. thuringiensis*^5^, is similar to the insecticidal mechanism of Cry. Second, the Trojan horse mechanism of *B. nematocida* lures nematodes through volatile organic compounds (VOCs) and uses two proteases, Bace16 and Bae16, to kill nematodes^6^. Third, the heat shock pathway and necrosis pathway is triggered by a two-domain protein Nep1^7^. Fourth, destruction of the nematode intestine is accomplished by a novel metalloproteinase ColB derived nematicidal *B. thuringiensis* YBT-1518^8^. However, how many types of nematicidal factors exist across the Bacilli and their mechanisms is unclear.

Spore-forming Bacilli with insecticidal or nematicidal activity usually contain multiple virulence factors that contribute to pathogenic effects through different mechanisms. For example, nematicidal Cry toxins, such as Cry5Ba2, Cry6Aa2 and Cry55Aa1, and metalloproteinase Bmp1 are all expressed in *B. thuringiensis* YBT-1518 and have been shown to kill nematodes through pore-forming and degradation mechanisms^9^,^10^. The different types of nematicidal factors use specific mechanisms, and comparison of their presence and absence across the Bacilli may elucidate the contributions of the corresponding mechanisms to pathogenic effects. Moreover, genomic-based methods have enhanced research strategies to clarify the mechanisms of bacterial pathogenicity in combination with assays for function^11^. For example, whole-genome sequencing of *B. thuringiensis* DB27 revealed multiple potential nematicidal factors, including novel Cry21Fa1 and Cry21Ha1 proteins encoded by different plasmids^12^. Systematic study of nematicidal capacity as well as genomic analysis of spore-forming Bacilli may have several advantages. Descriptions of the genomes of nematicidal bacteria have usually been limited to single genomes^9^,^12^,^13^, and multiple genomes from diverse nematicidal species have rarely been reported.

In this study, we sought to understand how diverse spore-forming Bacilli are nematicidal to nematodes. First, we collected 120 spore-forming Bacilli and compared their pathogenicity against *C. elegans in vitro* to determine their potential nematicidal capability. Second, we sequenced the genomes of these Bacilli and analyzed their genomes to identify potential virulence factors. This study revealed that spore-forming Bacilli appear to share virulence factors and nematicidal mechanisms at the genome level.

## Results

### 120 spore-forming Bacilli across two families show various nematicidal capacities

**Figure 1:**
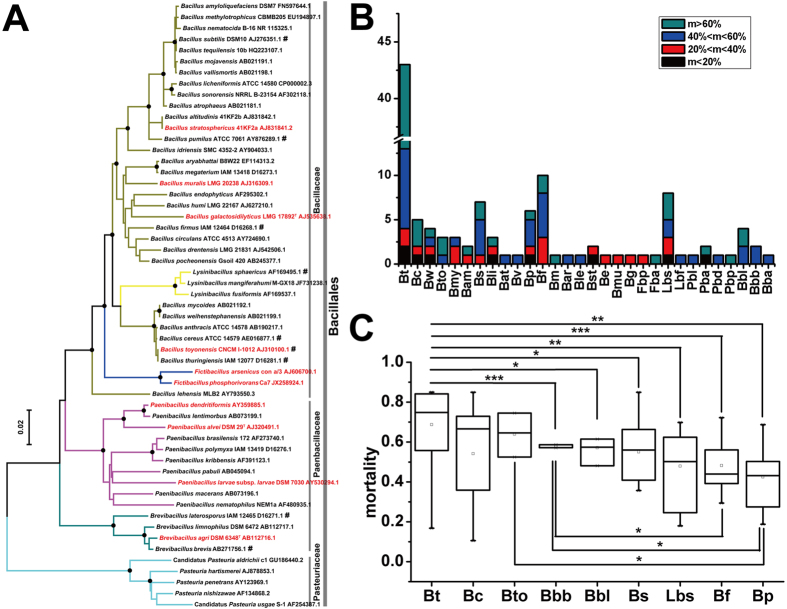
Diverse species of spore-forming Bacilli with various nematicidal activities. (**A**) Phylogenetic tree of 16S rRNA genes of nematicidal spore-forming Bacilli species. This tree does not include nematicidal *Pasteuria ramose* and *P. thornei*, which are shown in Table S1, because of a failure to obtain 16S rRNA genes of their type strains. Nodes supported with a bootstrap of ≥70% are represented by a black dot. The colors of branches represented different genera: olive drab (*Bacillus*), orange (*Lysinibacillus*), blue (*Fictibacillus*), purple (*Paenibacillus*), dark blue (*Brevibacillus*) and light blue (*Pasteuria*). The names in red represent ten new nematicidal species, and the names followed by ‘#’ represent nine highly nematicidal species. (**B**) Number of strains of the 30 spore-forming Bacilli species with various nematicidal activities. The nematicidal activities were classified into four ranks as following: mortality >60% (ultra-high, UH), 40%< mortality ≤60% (high, H), 20%< mortality ≤40% (medium, M) and 0< mortality ≤20% (low, L). (**C**) Comparison of the mortality of nine highly nematicidal species. Data are shown as means ± standard deviation of at least two strains. Bars indicate means ± standard deviation of at least two strains. The significance of differences among samples were evaluated using a two sample t-test at *p* < 0.001 (***), *p* < 0.01 (**) and *p* < 0.05 (*).

Up to 47 species of spore-forming Bacillaceae, Paenibacillaceae and Pasteuriaceae families of the Bacillales order have been found to have nematicidal activity (Fig. 1A, Fig. S1 and Table S1). To study the virulence mechanisms of Bacilli to nematodes, strains of nematicidal spore-forming Bacilli species across the three well-described families were obtained from other researchers or purchased from stock collections. We obtained a total of 120 spore-forming Bacilli.

The growth status of some strains from the collections was not consistent with their species’ characteristics, implying errors of classification in these strains. Thus, a re-identification through 16S rRNA genes was performed in this study. The 120 strains comprised 30 species across the Bacillaceae and Paenibacillaceae families of the Bacillales order, and 106 of the 120 strains comprised 20 out of the 47 above-mentioned species. The remaining 14 strains comprised 10 species that were not included in the 47 species (Fig. 1A, Table S1 and Table S4). Strains of the 10 species with classification errors were newly determined to be nematicidal, together with other strains, in the following bioassay. The 10 species tested were: *B. toyonensis, B. stratosphericus, B. muralis, B. galactosidilyticus, Fictibacillus phosphorivorans, F. arsenicus, Paenibacillus larvae, P. alvei, P. dendritiformis* and *Brevibacillus agri* (Fig. 1A and Table S1). Strains of the Pasteuriaceae family were not studied here because of their obligatory parasitism on root-knot nematodes.

To determine the potential nematicidal capacities of spore-forming Bacilli, a systematic bioassay was performed against *C. elegans in vitro*. Cultures of 120 spore-forming Bacilli, diluted by 10 times and 100 times, were tested for their nematicidal activity (Table S2). The results showed marked differences among the Bacilli in nematicidal capacities. To illustrate these differences, four ranks were defined: ultra-high (mortality > 60%, UH), high (40% < mortality ≤ 60%, H), medium (20% < mortality ≤ 40%, M) and low (0 < mortality ≤ 20%, L), according to the activity of the 10 times diluted cultures. Based on this classification, this bioassay showed that nematicidal species show a wide range of relative abilities. Five species in the *B. cereus* group (*B. thuringiensis, B. cereus, B. weihenstephanensis, B. toyonensis* and *B. mycoides*), three species in the *B. subtilis* group (*B. amyloliquefaciens, B. subtilis* and *B. licheniformis*), and *B. pumilus, B. firmus, L. sphaericus, P. alvei, B. laterosporus* and *B. brevis* showed a wide range of nematicidal ranks, other species showed a narrow range (Fig. 1B and Table S2). The results of classical survival tests on nematode growth medium (NGM) plates demonstrated that strains from UH, H, M and L category have similar differences in the 96-plates bioassays after 84 h (Fig. S2). In addition, more than half of the strains of 9 species, including *B. thuringiensis* (40/44), *B. cereus* (3/5), *B. toyonensis* (2/3), *B. subtilis* (5/6), *B. pumilus* (4/6), *B. firmus* (7/10), *L. sphaericus* (5/8), *B. laterosporus* (4/4) and *B. brevis* (2/2), killed nematodes at high or ultra-high levels (Fig. 1B). Nematicidal capability of *B. thuringiensis* was greater than that of the 6 species *B. pumilus, B. firmus, L. sphaericus, B. laterosporus, B. brevis* and *B. subtilis (p* < 0.01, *p* < 0.001, *p* < 0.01, *p* < 0.05, *p* < 0.001 and *p* < 0.05, two sample t-test, Fig. 1C), the nematicidal capability of *B. toyonensis* was greater than that of *B. pumilus (p* < 0.05, two sample t-test), and the nematicidal capability of *B. brevis* was greater than that of *B. firmus* and *B. pumilus (p* < 0.05 and *p* < 0.05, two sample t-test). These results demonstrated that spore-forming Bacilli species and strains have varied capacities to kill nematodes.

### General genomic features of the 120 nematicidal spore-forming Bacilli

The genomes of the 115 strains (the other five genomes are available in GenBank, including our 3 previously published genomes) were sequenced using an Illumina HiSeq2000 or HiSeq2500 platform in order to mine the genomes for potential nematicidal mechanisms. The genomes were sequenced to an average fold coverage of 138.8 and were assembled with AbySS^14^ into scaffolds with a range from 54 to 627 over 500 bp after gapfilling, containing a long-range continuity, as reflected by the N50 scaffold sizes from 19,349 bp of *B. thuringiensis* to 2,935,418 bp of *B. megaterium* (Table S3 and Table S4). The assembled genomes were then submitted to NCBI. The genomic DNA base compositions (GC content) ranged from 34.8% for *B. thuringiensis* to 54.53% for *P. dendritiformis*, and the genome sizes ranged from 3,586,955 bps for *B. stratosphericus* to 6,907,172 bps for *B. thuringiensis* (Table S4).

### 120 spore-forming Bacilli harbor five types of putative virulence factors

To predict the virulence mechanisms of these 120 spore-forming Bacilli, we sought to determine the presence of virulence factors in their genomes. We conducted a BLASTP search of known virulence factors that originated from animals, plants, fungi and bacteria (Table S5 and Table S6). The results demonstrate that spore-forming Bacilli harbor many putative virulence factors. These grouped into five categories: crystal proteins, compounds, proteases, chitinases and other proteins. More specifically, the five types of factors consisted of nematicidal crystal proteins predicted by our online database (http://bcam.hzaubmb.org/BtToxin_scanner/)^15^; adenine nucleoside derivative thuringiensin (β-exotoxin, gene cluster CT43_P127037-CT43_P127041)^16^; proteases, including alkaline serine protease Bace16 (AAV30845.1)^17^, neutral protease Bae16 (AAV30844.1)^18^, alkaline serine protease BLG4 (AAU81559.2)^19^, extracellular neutral proteases (Enp) NPE-4 (ABI93802.1)^20^ and Npr219 (ABI93803.1)^21^, metalloproteinase Bmp1 (AFZ77001.1)^10^ and collagenase metalloprotease ColB (AHA71938.1)^8^; chitinases, including Lpchi1 (ABQ57240.1)^22^, CrChi1 (ABV57861.1)^23^ and Chi46 (AAL78814.1)^24^; and other proteins, such as amidophosphoribosyltransferase PurL (NP_388531.2)^25^, calcium-transporting ATPase Eca1 (XP_572412.1)^26^ and two-domain protein Nel (AHZ54746.1)^7^, which is composed of a necrosis-inducing phytophthora protein 1-like domain and a ricin B-like lectin domain. Up to 1246 homologs were identified in the genomes of the 120 strains, with an average of 10 putative virulence factors per strain (Table S7).

**Figure 2:**
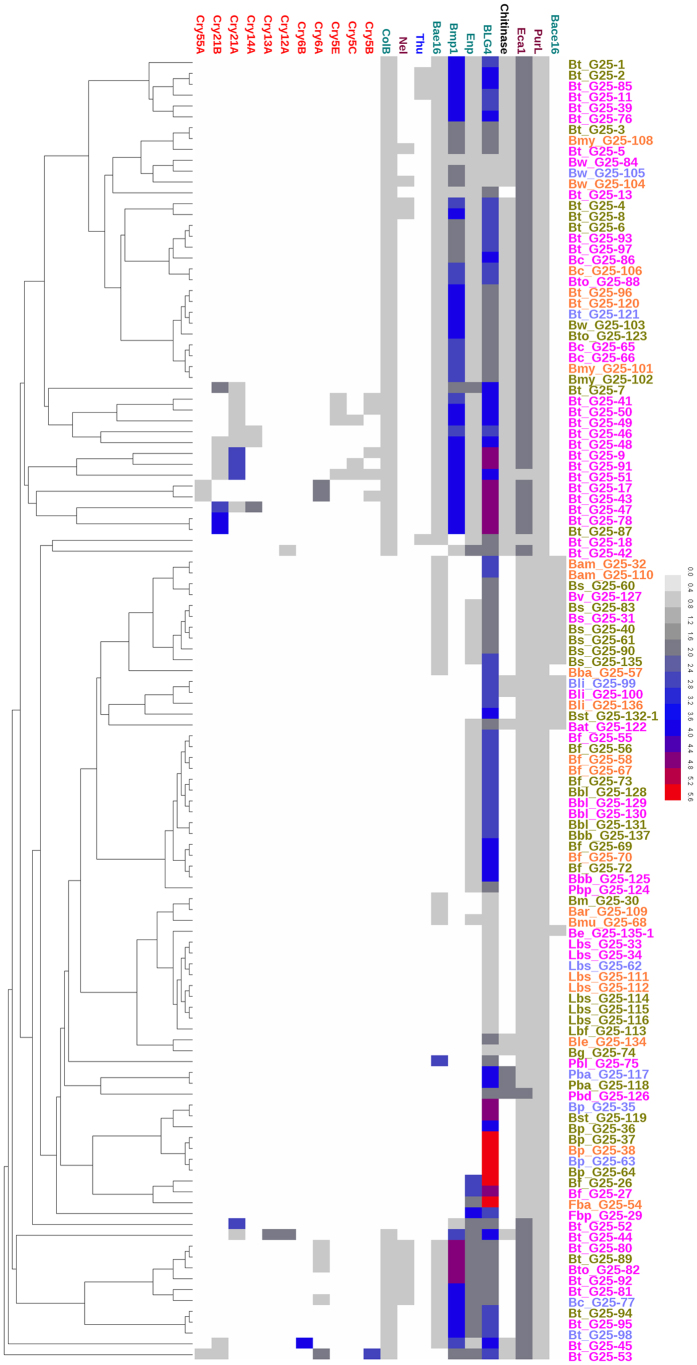
Putative virulence factors in the 120 spore-forming Bacilli. The 120 spore-forming strains and the 25 known virulence factors were clustered based on the number of homologs of nematicidal factors, which is shown in Table S6. Enp represents putative extracellular neutral protease, including NPE-4 (ABI93802.1) and Npr219 (ABI93803.1), and chitinase contains Lpchi1 (ABQ57240.1), CrChi1 (ABV57861.1) and Chi46 (AAL78814.1). Crystal proteins, thuringiensin, proteases, chitinases and other proteins are indicated by red, blue, cyan, black and violet, respectively. Strains with UH, H, M and L are indicated with pink, olive drab, orange and mediumslateblue, respectively. Strain names were abbreviated as follows: Bt for *B. thuringiensis*, Bc for *B. cereus*, Bw for *B. weihenstephanensis*, Bto for *B. toyonensis*, Bmy for *B. mycoides*, Bam for *B. amyloliquefaciens*, Bs for *B. subtilis*, Bli for *B. licheniformis*, Bat for *B. atrophaeus*, Bv for *B. vallismortis*, Bp for *B. pumilus*, Bf for *B. firmus*, Bm for *B. megaterium*, Bar for *B. aryabhattai*, Bl for *B. lehensis*, Bst for *B. stratosphericus*, Be for *B. endophyticus*, Bmu for *B. muralis*, Bg for *B. galactosidilyticus*, Fbp for *F. phosphorivorans*, Fba for *F. arsenicus*, Lbs for *L. sphaericus*, Lbf for *L. fusiformis*, Pbl for *P. larvae*, Pba for *P. alvei*, Pbd for *P. dendritiformis*, Pbp for *P. polymyxa*, Bbl for *B. laterosporus*, Bbb for *B. brevis* and Bba for *B. agri*. Each row corresponds to one strain, and each column shows the homologs of one virulence factor. Color of the heat map represents content of homologs.

The 120 spore-forming Bacilli had different numbers of potential virulence factors in their genomes, ranging from three factors in *L. sphaericus* to twenty-eight factors in *B. thuringiensis. B. thuringiensis* contained crystal proteins, including tertiary and quaternary ranks of Cry genes only from Cry5B, Cry5C, Cry5E, Cry6A, Cry6B, Cry12A, Cry13A, Cry14A, Cry21A, Cry21B and Cry55A subfamilies, and thuringiensin. Crystal proteins and thurigiensin coexisted in some strains (Fig. 2 and Table S6), such as UH and H nematicidal *B. thuringiensis* strains. Moreover, some UH nematicidal *B. thuringiensis* strains, such as *B. thuringiensis* G25-41 and G25-51, harbored up to 10 crystal protein genes. Other members of the *B. cereus* group had a truncated crystal protein gene *cry6A*, which was mainly found in *B. cereus*. Although there were only five types of proteases, their distributions were complex. *B. amyloliquefaciens, B. subtilis* and *B. vallismortis* of the *B. subtilis* group, which were close to *B. nematocida* in the phylogenetic tree (Fig. 1A), contained homologs of the later’s protease Bace16 and Bae16. However, *B. licheniformis, B. atrophaeus, B. stratosphericus*, and *B. endophyticus* harbored homologs of Bace16, and homologs of Bae16 were notably present in the genomes of the *B. cereus* group, *B. megaterium, B. aryabhattai, B. muralis, P. larvae* and *B. agri*. Almost all strains of the *B. cereus* group contained one to five homologs of metalloproteinase Bmp1, but *B. thuringiensis* G25-18 had none. However, putative extracellular neutral protease Enp and extracellular alkaline serine protease BLG4 were distributed more widely. Chitinase Lpchi1, CrChi1 and Chi46 degrade chitinous components of the egg or cuticle of nematodes^22^,^23^,^27^. The *B. cereus* group genomes harbored the most chitinase homologs. Fifty-nine homologs were identified in 120 spore-forming Bacilli and were clustered into three groups based on their domain compositions (Fig. S3 and Table S9). These homologs from spore-forming strains shared the same GH18_chitinase domain as chitinases from nematode-parasitic fungi. In the group of factors classified as other proteins, the homologs of virulence protein Nel^7^ was present primarily in UH and H nematicidal *B. cereus* group strains. However, the homologs of amidophosphoribosyltransferase PurL^25^ and calcium-transporting ATPase Eca1^26^ existed in all genomes of the 120 strains with low contents. These features demonstrated that numerous diverse virulence factors may underlie various nematicidal capabilities of spore-forming Bacilli. Although the actual activities of these putative virulence factors have not been determined here, their presences in the genomes indicate their potential roles in nematicidal activity.

### Four putative virulence mechanisms may contribute to the nematicidal capacity of spore-forming Bacilli

**Figure 3:**
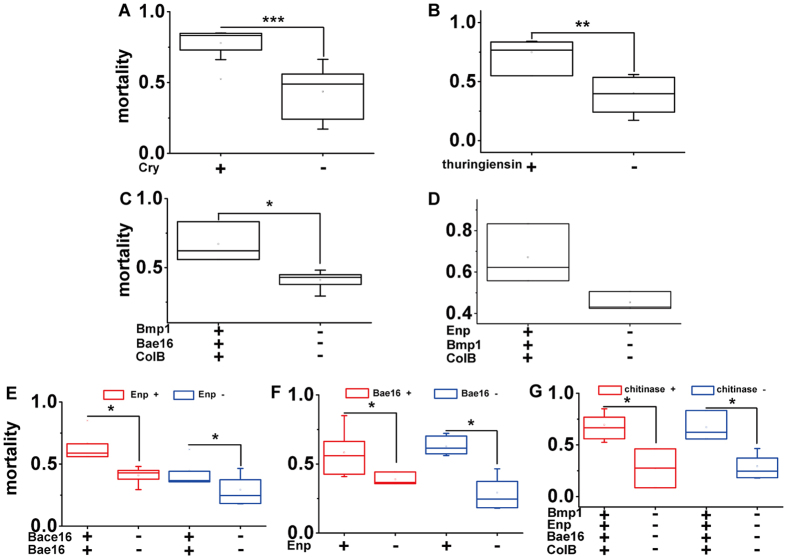
Relationships between the presence of putative virulence factors and nematicidal capacities. Correlations between virulence factors and capacities are based on the combination of putative virulence factors and nematicidal activity (Table S8). Bars represent the standard error given the sampling size of strains in each mechanism. Comparisons were carried out between factor-producing strains and non-producing strains. (**A**) Pore-forming mechanism of Cry, (**B**) Inhibition-like mechanism of thuringiensin, (**C**) Degradation mechanism of putative Bmp1, Bae16 and ColB, (**D**) Degradation mechanisms of putative Enp, Bmp1 and ColB, (**E**) Trojan horse mechanism (right) and combined degradation mechanism of Trojan horse mechanism and putative Enp (left), (**F**) Combined degradation mechanism of putative Enp and Bae16 (left) and degradation mechanism of putative Enp (right), (**G**) Combined degradation mechanism of putative Enp, Bmp1, Bae16, ColB and chitinases (left) and degradation mechanism of putative Enp, Bmp1, Bae16 and ColB (right). Data are shown as the means ± the standard deviation of at least three strains. Bars indicate the means ± standard deviation of at least three strains. The significance of differences among samples was evaluated using a two sample t-test at *p* < 0.001 (***), *p* < 0.01 (**) and *p* < 0.05 (*).

The diversity of potential virulence factors found suggests that multiple mechanisms may exist in these nematicidal spore-forming Bacilli. Determining these mechanisms is important for understanding the virulence of spore-forming Bacilli to nematodes. We combined genomic analysis and the nematicidal capacity of the 120 strains and performed a series of comparisons of nematicidal capacity between factor-producing strains and non-producing strains, both of which have same backgrounds of other virulence factors. Four possible virulence mechanisms were deduced based on these comparisons, including the pore-forming mechanism of crystal proteins, the inhibition-like mechanism of thuringiensin, and the degradation mechanisms of proteases and chitinases (Fig. 3). Combinations of these mechanisms occurred in some strains.

The pore-forming mechanism of Cry was present in *B. thuringiensis*. In nematicidal *B. thuringiensis*, three subfamilies of crystal proteins toxic to nematodes (Cry5B, Cry5C, Cry5E, Cry12A, Cry13A, Cry14A, Cry21A and Cry21B in Cry5 subfamily, Cry6A and Cry6B in Cry6 subfamily and Cry55A in Cry55 subfamily) were found, indicating that these strains may kill nematodes through a pore-forming mechanism exerted by Cry proteins, such as Cry5B, which causes lysis of the intestine and nematode death after interaction with specific receptors^28^,^29^. Strains expressing nematicidal Cry toxins killed nematodes with a mortality of 77.92 ± 9.44% higher than that of 43.71 ± 17.75% of *cry* non-coding *B. thuringiensis* strains (Fig. 3A and Table S8) (*p* < 0.001, two sample t-test). In addition, combinations of three subfamilies of crystal proteins show synergistic activity against *C. elegans* in some strains^30^ (Table S7).

The inhibition-like mechanism of thuringiensin also existed in *B. thuringiensis*. Thuringiensin (β-exotoxin) is an adenine nucleoside derivative produced by *B. thuringiensis* that contributes to insecticidal abilities through the inhibition of RNA polymerases by competing with ATP for binding sites^16^. Thuringiensin can be used to control *Heterodera glycines* in soybeans and shows toxicity to *C. elegans* and *Pristionchus pacificus*^31^,^32^. Nematicidal strains containing the thuringiensin synthesizing cluster killed nematodes with a mortality of 74.86 ± 13.68%, which was higher than the 39.93 ± 16.06% mortality for thuringiensin non-synthesizing strains (Fig. 3B and Table S8) (*p* < 0.01, two sample t-test).

Virulence proteases, such as the homologs of metalloproteinase Bmp1 and extracellular neutral protease Enp, may digest proteins from the nematode’s intestines and cuticles, respectively. Spore-forming Bacilli usually harbored diverse protease genes, which may lead to multiple degradation mechanisms with different combinations of the same type or multiple types. The Trojan horse mechanism, which has been found in *B. nematocida*^6^,^21^, may also exist in nematicidal *B. amyloliquefaciens, B. subtilis* and *B. vallismortis* because some strains of these species harbored homologs of both extracellular alkaline serine protease Bace16 and neutral protease Bae16. Through this mechanism, once bacteria enter the intestine of nematodes, they secrete Bace16 and Bae16, both of which have broad substrate ranges but preferentially target essential intestinal proteins, thus leading to nematode death^6^. Nematicidal spore-forming strains with putative Bace16 and Bae16 killed nematodes with a mortality of 44.62 ± 12.08%, which was higher than the 29.29 ± 11.58% mortality for the putative Bace16 and Bae16 non-encoding strains (Fig. 3E_right and Table S8) (*p* < 0.05, two sample t-test). Combinations of different virulence proteases may exist in the nematicidal spore-forming Bacilli. For example, both the metalloproteinase Bmp1 and the neutral protease Bae16 degrade the nematode intestine^10^,^18^. Nematicidal *B. thuringiensis* strains harboring genes for the proteases Bmp1, Bae16 and ColB killed nematodes with a mortality of 67.12 ± 14.38%, which was higher than the 40.89 ± 5.87% mortality for Bmp1, Bae16 and ColB non-encoding *B. thuringiensis* strains (Fig. 3C and Table S8) (*p* < 0.05, two sample t-test).

Enp is a neutral protease that has been found in *Bacillus* sp. strain RH219^21^. Purified Enp kills nematodes, causing low mortality by digesting the cuticle of the nematodes, but it may produce increased mortality in conjunction with another virulence protease. The putative degradation mechanism of Enp may exist in some strains of *B. firmus, F. phosphorivorans, F. arsenicus, B. brevis*, and *B. laterosporus*. Strains with genes for Enp killed nematodes with a mortality of 62.81 ± 6.32%, which was higher than the 29.29 ± 11.58% mortality for putative Enp non-encoding strains in the absence of putative Bae16 (Fig. 3F_right and Table S8) (*p* < 0.05, two sample t-test).

The systematical bioassay and genome resource of the 120 spore-forming Bacilli provided an opportunity to characterize correlations between presence of virulence genes and nematicidal capacity indirectly. We identified combinations of virulence proteases that may enhance nematicidal capacity by facilitating digestion of both the intestine and cuticle. For example, a combination of the putative Trojan horse mechanism and putative Enp degradation, may exist in some *B. subtilis* strains. Nematicidal *B. subtilis* strains with this combination killed nematodes with a mortality of 66.51 ± 13.04%, which was higher than the 40.89 ± 5.87% mortality for putative Bace16 and Bae16 non-encoding strains (Fig. 3E_left and Table S8) (*p* < 0.05, two sample t-test). In addition, the coexistence of putative Enp and Bae16 in *B. subtilis*, led to a mortality of 58.26 ± 16.31%, which was higher than the 38.89 ± 4.69% mortality for putative Enp non-encoding strains (Fig. 3F_left and Table S8) (*p* < 0.001, two sample t-test). Furthermore, *B. thuringiensis* containing putative Enp, Bmp1 and ColB, killed nematodes with a mortality of 67.12 ± 14.38%, which was higher than the 45.4 ± 4.51% mortality for putative Enp, Bmp1 and ColB non-encoding strains (Fig. 3D and Table S8) (*p* = 0.05479, two sample t-test, no significant differences). Finally, the combination of putative Enp, Bmp1, Bae16 and ColB, had a mortality of 67.12 ± 14.38%, which was higher than the 29.29 ± 11.58% mortality for putative Enp, Bmp1, Bae16 and ColB non-encoding strains (Fig. 3G_right and Table S8) (*p* < 0.05, two sample t-test).

Putative virulence chitinases may digest chitinous components of the eggs of the nematodes. Homologs of Lpchi1, CrChi1 and Chi46 also existed in spore-forming Bacilli and may kill nematodes through a combination of Enp, Bmp1, Bae16, ColB and chitinase expression in *B. thuringiensis, B. cereus, B. weihenstephanensis* and *B. toyonensis*. Strains with this combination killed nematodes with a mortality of 69.56 ± 11.85%, which was higher than the 27.46 ± 18.8% mortality for putative Enp, Bmp1, Bae16 and ColB non-encoding strains (Fig. 3G_left and Table S8) (*p* < 0.05, two sample t-test).

These results provided evidence that expression of multiple putative virulence mechanisms may contribute to the pathogenesis of spore-forming Bacilli to nematodes. Among these mechanisms, the degradation mechanisms of Enp are found in nematicidal species in *Bacillus, Fictibacillus* and *Brevibacillus* across the Bacillaceae and Paenbacillaceae families; moreover, the Trojan horse mechanism is mainly found in the *B. subtilis* group and the degradation mechanisms of chitinases are mainly found in the *B. cereus* group, thus suggesting that different species of spore-forming Bacilli may share mechanisms to kill nematodes.

## Discussion

*B. thuringiensis* is a pathogen, which can also kill nematodes with different nematicidal factors and mechanisms^33^. However, the pathogenicity of other spore-forming bacteria in nematodes is not well known. A survey of the published literature showed that 47 species from the spore-forming Bacillaceae, Paenbacillaceae and Pasteuriaceae families of the Bacillales order have nematicidal activity. In soil, free-living nematodes prey on bacteria, including spore-forming Bacilli. These genomes of these spore-forming Bacilli may encode virulence mechanisms against nematodes, including the obligatory parasitism of the Pasteuriaceae family to root knot nematodes^34^. Therefore, we believe that spore-forming Bacilli are a good resource for the identification and study of nematicidal bacteria.

Environmental adaptation can shape the ecological diversity of *Bacillus* spp.^35^ and the trophic interactions between these bacteria and their consumers can drive ecological differentiation^33^. To adapt to nematode-associated environmental niches, spore-forming species may utilize virulence factors to kill nematodes. Our results showed that spore-forming Bacilli genomes can encode several putative virulence factors that may be used to kill nematodes, either independently or in combination. Furthermore, these nematicidal species may have similar pathogenesis, necromeny, and phoresy to *B. thuringiensis*, as described in our recent review paper^33^, and the presence of different virulence factors in the genome may correlate to one of the three lifestyles. The existence of nematicidal crystal protein toxins and thuringiensin in *B. thuringiensis* provides evidence that nematicidal species have pathogenic lifestyles arising from different mechanisms. For example, Cry6Aa triggers a *C. elegans* necrosis pathway mediated by aspartic protease (ASP-1)^36^. The presence of diverse putative virulence serine proteases and chitinases, which may digest nematode cuticle and eggs, in the nematicidal species showed that necromenic lifestyles may be ubiquitous among these spore-forming Bacilli (Table S7). In addition, conversion among different lifestyles may occur. For example, some strains that had same potential virulence factors as highly nematicidal strains exhibited low toxicity, indicating that these strains may be at the stage of phoresy but have the potential for an evolutionary shift toward pathogenesis or necromeny (Table S2).

How diverse spore-forming Bacilli evolve nematicidal capabilities is not clear, although there are some clues provided by our analysis. First, we found that nematicidal *B. amyloliquefaciens, B. subtilis* and *B. vallismortis* of the *B. subtilis* group, which are close to *B. nematocida*, shared homologs of both of the latter’s Bace16 and Bae16. However, Bace16 existed in nematicidal *B. licheniformis, B. atrophaeus, B. stratosphericus*, and *B. endophyticus*, and Bae16 appeared in the genomes of the *B. cereus* group, *B. megaterium, B. aryabhattai, B. muralis, P. larvae* and *B. agri*. Second, in the spore-forming mechanism of Cry, *B. thuringiensis* evolved three types of nematicidal crystal proteins with no sequence similarity, including the Cry5 subfamily, Cry6 subfamily and Cry55 subfamily. The toxicity of these crystal proteins to nematodes correlates with damage to the intestine, consistently with the mechanism of crystal toxin action in insects. Although they kill nematodes through similar pore-forming mechanisms, the crystal proteins of three subfamilies have different properties based on their structures. The Cry5 subfamily crystal proteins except Cry5A, such as Cry5B, binds nematode-specific glycolipids through the unusual domain II in the familiar three-domain arrangement seen in insecticidal Cry proteins^28^,^29^,^37^. The Cry6 subfamily crystal proteins, such as Cry6A, contain an unusually small active toxin core with a predicted molecular mass of 43 kDa^28^. No conserved domains match the Cry55Aa from Cry55 subfamily, but our research has revealed a synergistic activity between Cry6Aa and Cry55Aa toxins against *Meloidogyne incognita*^30^. Although 16S rDNA is not sufficient to discern evolutionary relationships among bacteria, the scattered distribution of virulence factors among these strains suggests that these may be ancestral genes or that the acquisition of virulence factor genes may occur during the interactions of spore-forming Bacilli and other nematicidal microorganisms. Although the evolution of nematicidal traits need further study, the results in this work demonstrate that 120 spore-forming Bacilli across different families share virulence factors that may contribute to their nematicidal capacity. These virulence factors may participate in bacterial pathogenesis in nematodes through diverse mechanisms. In addition to the homologs of known virulence factors, there may be other nematicidal factors in spore-forming Bacilli. Some spore-forming strains, such as *L. sphaericus* G25-33, G25-34 and G25-62, had few identified nematicidal virulence factors but showed high nematicidal activity. This phenomenon indicates that these spore-forming strains may have some undescribed virulence factors, which will be studied further.

## Methods

### Strains, nematodes and toxicity assays

The 120 spore-forming Bacilli were selected according to previous studies, as determined by the number of studies published related to the nematicidal spore-forming Bacilli species across Bacillaceae, Paenibacillaceae and Pasteuriaceae (Table S1). We obtained the strains from other researchers or purchased them from stock collections (Table S4). The *C. elegans* N2 wild-type strain was provided by the Caenorhabditis Genetics Center (CGC) and was maintained at 20 °C on NGM agar plates with *E. coli* OP50 as a food source. The whole cultures of spore-forming Bacilli were tested against *C. elegans* N2. Mortality was assayed according to the method of Bischof *et al*.^38^ with some modifications. Bacterial cultures incubated in Luria-Bertani (LB) liquid medium at 28 °C after 48 hrs were used as the bioassay samples. After adjustment by OD600, the original cultures, 10-times diluted cultures, and 100-times diluted cultures were tested against *C. elegans* in triplicate. The bioassay mixtures contained 150 μl culture and 40 μl *E. coli* OP50 which may be important for basic growth of *C. elegans* at a final absorbance at 600 nm of 0.6 in S medium, 5 μl of L4 N2 worms in M9 medium and 5 μl of 5-fluoro-2′-deoxy-uridine (FUdR, Alfa Aesar, Tianjin, China, cat. no. M0103). 200 μl *E. coli* OP50 was used as a control. Incubation was performed in 96-well microtiter plates (Corning, 3513), which were enclosed with parafilm to maintain the appropriate humidity, at 20 °C for 3 days. Worm viability was determined by observing their movement. A visibly moving worm was defined as being alive. Worms that were not moving were lightly touched with a platinum pick to confirm their movement. Worms that failed to respond after several touches were defined as dead^38^. The mortality of nematodes was defined as the ratio of dead nematodes over tested nematodes^18^. 30 μl of overnight spore-forming Bacilli cultures were spread evenly over the surface of NGM plates and incubated at 20 °C overnight for survival tests of nematodes. The following morning 20–30 L4 stage *C. elegans* were placed onto three separate plates and survival was recorded every 12 h for 84 hrs. The survival of nematodes fed OP50 was also tested using the same procedures as a control.

### Whole genome sequencing and annotation

The total genomic DNA of 115 nematicidal spore-forming strains was extracted from the cells according to the procedure of Andrup *et al*.^39^. The sequences were generated using Illumina HiSeq 2000 technology. Sequence data were assembled using AbySS^14^. K-mer was debugged to obtain an optimized N50 in each assembly. The gapped scaffold sequences after draft assembly were almost closed with original data by GapFiller software^40^. Gene prediction and genome annotation were performed with the Rapid Annotation Subsystem Technology server (http://rast.nmpdr.org/rast.cgi)^41^ and Prokka software.

### Phylogenetic tree construction

Sequences of the type strain 16S rRNA genes of all nematicidal spore-forming Bacilli species were retrieved from the Greengenes database^42^. Sequences of virulence chitinases of spore-forming Bacilli were extracted from genomes, and the sequences of fungal nematicidal chitinases were retrieved from GenBank (http://www.ncbi.nlm.nih.gov/protein/). Both sequences of 16S rRNA genes and virulence chitinases were used for phylogenetic tree construction. Two maximum likelihood phylogenetic trees were generated by the MEGA 5.03 software^43^ with bootstrap support calculated from 500 replicates after sequences were aligned by ClustalW^44^.

### Identification of virulence factors

Known virulence factors are proteins or compounds that have been reported to be toxic to any type of nematodes. They were mined from the published literature, and their sequences were obtained from GenBank (http://www.ncbi.nlm.nih.gov/protein/) and Cybase: the Cyclotide Database. The nematicidal Cry genes were identified by our online database (http://bcam.hzaubmb.org/BtToxin_scanner/)^15^. The sequence IDs and the corresponding references are listed in Table S5. Homologous sequences of known virulence factors were extracted from the analyzed genomes by sequence similarity using Blastp with an e-value of 10^−5^ and at least 30% sequence identity^45^ over 60% of both protein lengths^46^.

### Sequence alignment and protein domain prediction

Representative sequences of the four groups of virulence chitinases were used for complete alignment with ClustalX^47^. All sequences of virulence chitinases were used for domain prediction by the conserved domain database (CDD) of GenBank, with an e-value of 0.01 and a maximum number of hits of 500. The schematic diagram of domains were generated by the DOG software^48^.

## Additional Information

**Accession codes:** The genome sequences of the 115 strains which were sequenced in this study have been deposited under the Genbank accession numbers.

**How to cite this article**: Zheng, Z. *et al*. Nematicidal spore-forming Bacilli share similar virulence factors and mechanisms. *Sci. Rep.* **6**, 31341; doi: 10.1038/srep31341 (2016).

## Supplementary Material

Supplementary Information
