# Supplementary material for: Nematicidal spore-forming Bacilli share similar virulence factors and mechanisms
Source: Sci Rep. 2016 Aug 19;6:31341. doi: 10.1038/srep31341 (PMC4990965; doi:10.1038/srep31341)
Supplement: Supplementary Information [file srep31341-s1.doc]

**Supplementary information**

**Nematicidal spore-forming Bacilli share similar virulence factors and mechanisms**

Ziqiang Zheng, Jinshui Zheng, Zhengming Zhang, Donghai Peng and Ming Sun^*^

State Key Laboratory of Agricultural Microbiology, College of Life Science and Technology, Huazhong Agricultural University, Wuhan 430070, China

*Corresponding author

State Key Laboratory of Agricultural Microbiology, College of Life Science and Technology, Huazhong Agricultural University, Wuhan 430070, China. Phone: 86-27-87283455. Fax: 86-27-87280670. E-mail: m98sun@mail.hzau.edu.cn.

**Supplementary Figure S1-S3**

**
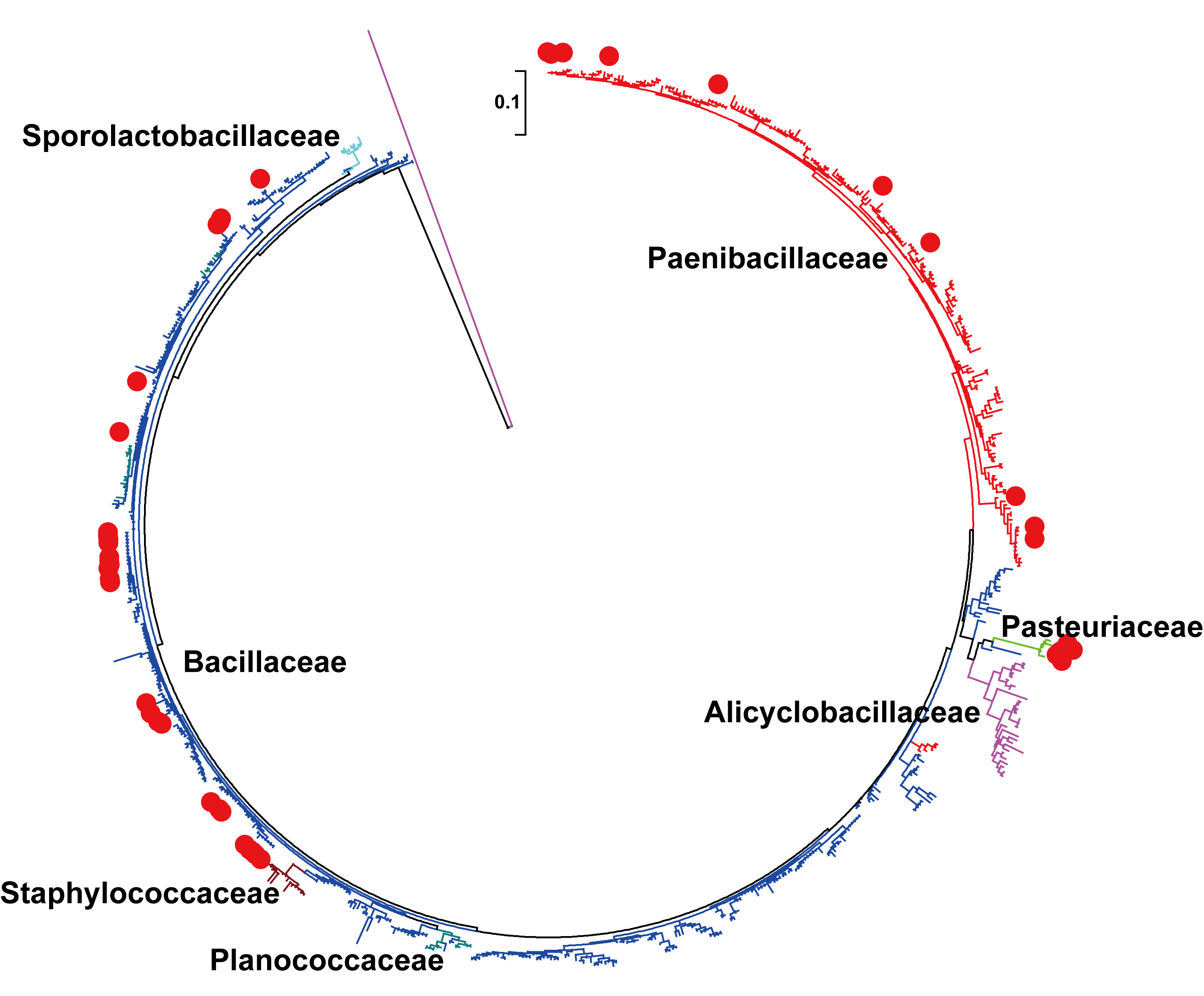
**

**Figure S1** **16S rRNA gene phylogeny of spore-forming Bacilli with full names and bootstrap support** Maximum likelihood tree based on 16S rRNA gene sequences from type strains of each species of spore-forming Bacilli download from LPSN. The nematicidal species are indicated with red dots. Seven families are indicated with the following color: Paenibacillaceae (red), Pasteuriaceae (green), Alicyclobacillaceae (pink), Bacillaceae (blue), Staphylococcaceae (darkblue), Planococcaceae (saddlebrown), Sporolactobacillaceae (lightgreen).


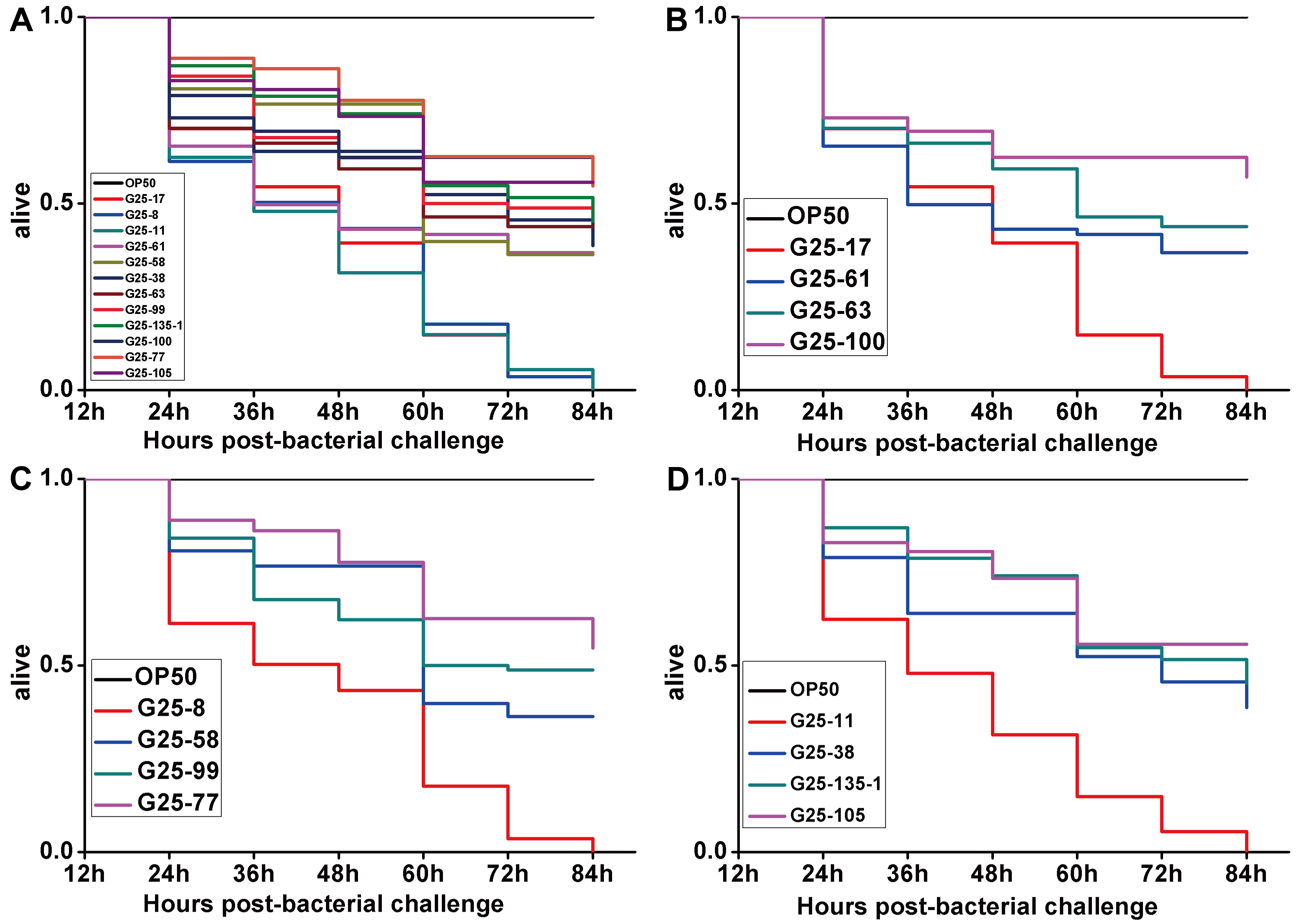


**Figure S2 Survival tests of nematodes on NGM plates with three strains from each category of UH, H, M and L.** *B. thuringiensis* G25-17, G25-8 and G25-11 of UH, *B. subtilis* G25-61, *B. firmus* G25-58 and *B. pumilus* G25-38 of H, *B. pumilus* G25-63, *B. licheniformis* G25-99 and *B. endophyticus* G25-135-1 of M and *B. licheniformis* G25-100, *B. cereus* G25-77 and *B. weihenstephanensis* G25-105 of L category have been performed in the tests. (**A**) The survival of nematodes fed all the twelve strains. (**B**)- (**D**) Decomposed schematic diagrams of survival of nematodes for clear comparisons between different categories. The survival of nematodes fed each *Bacillus* strain is the same of the corresponding record in (A). Three strains used for comparisons between the different categories were combined randomly.


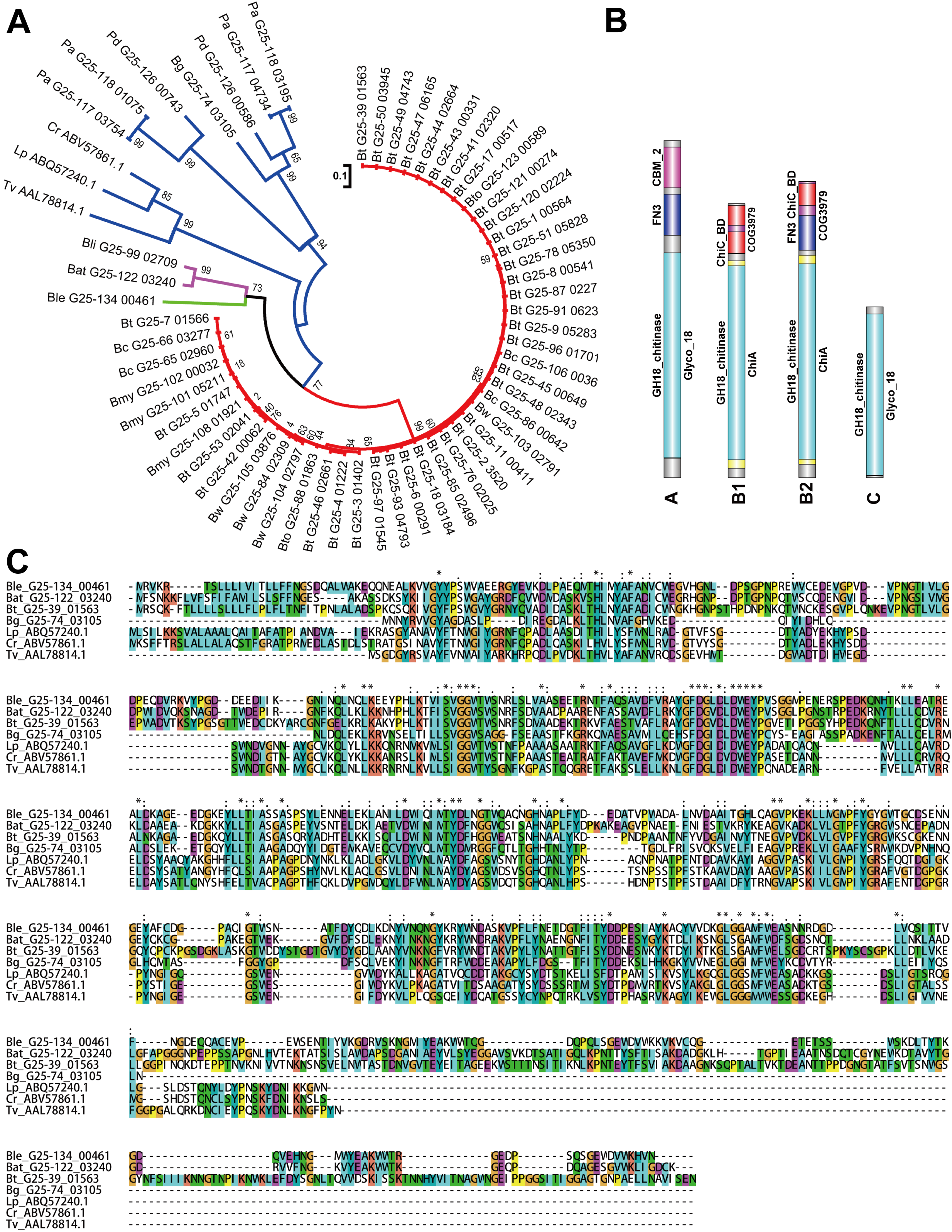


**Figure S3 Analysis of putative virulence chitinases from spore-forming Bacilli** (**A**) Phylogenetic tree of virulence chitinases from spore-forming Bacilli and fungi. The colors of branches mean different groups: red, homologs from *B. cereus* group; olive drab, homologs from *B. lehensis*; purple, homologs from *B. licheniformis* and *B. atrophaeus*, blue, homologs in *Paenibacillus* and fungi. Red, oliver drab, purple and blue represent group A, B1, B2 and C, respectively. (**B**) The schematic diagram of domains of virulence chitinases. The domains of homologs of virulence chitinases are shown in Table S9. (**C**) The sequence alignment of virulence chitinases from spore-forming Bacilli and fungi. The 51 conserved sites, which include 10 active sites (Y43, F69, D164, D167, E171, M237, Y239, D240, Y293 and W378 in GH18_chitinase domain of chitinase Lpchi1) are indicated by stars.

**Supplementary Table S1-S9**

**Table S1** Survey of spore-forming Bacilli with nematicidal activity

| class | order | family | genus | species***** | group***** | reference |
| --- | --- | --- | --- | --- | --- | --- |
| Bacilli | Bacillales | Bacillaceae | *Bacillus* | *B. idriensis* |  | [^1^](#_ENREF_1) |
|  |  |  |  | *B. altitudinis* |  | [^1^](#_ENREF_1) |
|  |  |  |  | *B. aryabhattai* |  | [^1^](#_ENREF_1) |
|  |  |  |  | *B. cereus* | *B. anthracis* | [^1^](#_ENREF_1) |
|  |  |  |  |  | *B. toyonensis* | new |
|  |  |  |  |  | *B. mycoides* | [^2-4^](#_ENREF_2) |
|  |  |  |  |  | *B. cereus* |  |
|  |  |  |  |  | *B. thuringiensis* |  |
|  |  |  |  |  | *B. weihenstephanensis* |  |
|  |  |  |  | *B. drentensis* |  | [^1^](#_ENREF_1) |
|  |  |  |  | *B. endophyticus* |  | [^13^](#_ENREF_13) |
|  |  |  |  | *B. megaterium* |  |  |
|  |  |  |  | *B. pumilus* |  |  |
|  |  |  |  | *B. stratosphericus* |  | new |
|  |  |  |  | *B. subtilis* | *B. amyloliquefaciens* |  |
|  |  |  |  |  | *B. atrophaeus* | [^13^](#_ENREF_13) |
|  |  |  |  |  | *B. licheniformis* | [^11^](#_ENREF_11) |
|  |  |  |  |  | *B. mojavensis* | [^13^](#_ENREF_13) |
|  |  |  |  |  | *B. sonorensis* | [^1^](#_ENREF_1) |
|  |  |  |  |  | *B. subtilis* |  |
|  |  |  |  |  | *B. vallismortis* | [^13^](#_ENREF_13) |
|  |  |  |  | *B. methylotrophicus* |  | [^5^](#_ENREF_5) |
|  |  |  |  | *B. pocheonensis* |  | [^5^](#_ENREF_5) |
|  |  |  |  | *B. tequilensis* |  | [^5^](#_ENREF_5) |
|  |  |  |  | *B. lehensis* |  | [^18^](#_ENREF_18) |
|  |  |  |  | *B. circulans* |  |  |
|  |  |  |  | *B. firmus* |  |  |
|  |  |  |  | *B. galactosidilyticus* |  | new |
|  |  |  |  | *B. muralis* |  | new |
|  |  |  |  | *B. nematocida* |  |  |
|  |  |  |  | *B. humi* |  | [^1^](#_ENREF_1) |
|  |  |  | *Lysinibacillus* | *L. fusiformis* |  | [^1^](#_ENREF_1) |
|  |  |  |  | *L. sphaericus* |  | [^5^](#_ENREF_5) |
|  |  |  |  | *L. mangiferahumi sp. nov.* |  | [^23^](#_ENREF_23) |
|  |  |  | *Fictibacillus* | *F. arsenicus* |  | new |
|  |  |  |  | *F. phosphorivorans* |  | new |
|  |  | Paenbacillaceae | *Brevibacillus* | *B. agri* |  | new |
|  |  |  |  | *B. brevis* |  | [^24^](#_ENREF_24) |
|  |  |  |  | *B. laterosporus* |  | [^25^](#_ENREF_25) |
|  |  |  |  | *B. limnophilus* |  | [^13^](#_ENREF_13) |
|  |  |  | *Paenibacillus* | *P. alvei* |  | new |
|  |  |  |  | *P. dendritiformis* |  | new |
|  |  |  |  | *P. larvae* |  | new |
|  |  |  |  | *P. lentimorbus* |  |  |
|  |  |  |  | *P. polymyxa* |  |  |
|  |  |  |  | *P. kribbensis* |  | [^13^](#_ENREF_13) |
|  |  |  |  | *P. brasilensis* |  | [^13^](#_ENREF_13) |
|  |  |  |  | *P. nematophilus* |  | [^29-31^](#_ENREF_29) |
|  |  |  |  | *P. pabuli* |  | [^32^](#_ENREF_32) |
|  |  |  |  | *P. macerans* |  |  |
|  |  | Pasteuriaceae | *Pasteuria* | *P. nishizawae* |  |  |
|  |  |  |  | *P. hartismerei* |  |  |
|  |  |  |  | *P. penetrans* |  |  |
|  |  |  |  | *P. ramose* |  | [^37^](#_ENREF_37) |
|  |  |  |  | *P. thornei* |  | [^36^](#_ENREF_36) |
|  |  |  |  | *Candidatus* P. aldrichii |  |  |
|  |  |  |  | *Candidatus* P. usgae |  |  |

***** black: representative of known nematicidal spore-forming Bacilli species with nematicidal activity which were published previously by the related authors.

red: representative of new nematicidal spore-forming Bacilli species identified only in this study.

References

1 Li, M. A strategy to discover potential nematicidal fumigants based on toxic volatiles from nematicidal bacteria. *Afr J Microbiol Res* **6** (2012).

2 Rae, R., Iatsenko, I., Witte, H. & Sommer, R. J. A subset of naturally isolated *Bacillus* strains show extreme virulence to the free-living nematodes *Caenorhabditis elegans* and *Pristionchus pacificus*. *Environ Microbiol* **12**, 3007-3021 (2010).

3 Rae, R., Witte, H., Rödelsperger, C. & Sommer, R. J. The importance of being regular: *Caenorhabditis elegans* and *Pristionchus pacificus* defecation mutants are hypersusceptible to bacterial pathogens. *Int J Parasitol* **42**, 747-753 (2012).

4 Insunza, V., Alström, S. & Eriksson, K. Root bacteria from nematicidal plants and their biocontrol potential against trichodorid nematodes in potato. *Plant Soil* **241**, 271-278 (2002).

5 Ma, L. *et al.* Phylogenetic diversity of bacterial endophytes of *Panax notoginseng* with antagonistic characteristics towards pathogens of root-rot disease complex. *Antonie van Leeuwenhoek* **103**, 299-312 (2013).

6 Almaghrabi, O. A., Massoud, S. I. & Abdelmoneim, T. S. Influence of inoculation with plant growth promoting rhizobacteria (PGPR) on tomato plant growth and nematode reproduction under greenhouse conditions. *Saudi J Biol Sci* **20**, 57-61 (2013).

7 Sinott, M. C. *et al.* *Bacillus* spp. toxicity against *Haemonchus contortus* larvae in sheep fecal cultures. *Exp Parasitol* **132**, 103-108 (2012).

8 Seo, B. J. Bacterial mixture from greenhouse soil as a biocontrol agent against root-knot nematode, *Meloidogyne incognita*, on oriental melon. *J Microbiol Biotechnol* **22**, 114-117 (2012).

9 Oliveira, D. F. *et al.* Selection of rhizobacteria able to produce metabolites active against *Meloidogyne exigua*. *Eur J Plant Pathol* **119**, 477-479 (2007).

10 Tian, H. & Riggs, R. D. Effects of rhizobacteria on soybean cyst nematode, *Heterodera glycines*. *J Nematol* **32**, 377-388 (2000).

11 Siddiqui, Z. & Mahmood, I. Role of bacteria in the management of plant parasitic nematodes: a review. *Bioresour Technol* **69**, 167-179 (1999).

12 Linares, I. H., Arellano, M. E. L., de Gives, P. M., Hernández, E. L. & de la Parra, A. B. Lethal activity of two *Bacillus thuringiensis* strains against *Haemonchus contortus* histotropic larvae. *Animal Biodiversity and Emerging Diseases: Ann N Y Acad Sci* **1149**, 164-166 (2008).

13 Köberl, M. *et al.* *Bacillus* and *Streptomyces* were selected as broad-spectrum antagonists against soilborne pathogens from arid areas in Egypt. *FEMS Microbiol Lett* **342**, 168-178 (2013).

14 Neipp, P. W. & Becker, J. O. Evaluation of biocontrol activity of rhizobacteria from *Beta vulgaris* against *Heterodera schachtii*. *J Nematol* **31**, 54-61 (1999).

15 El-Hadad, M. *et al.* The nematicidal effect of some bacterial biofertilizers on *Meloidogyne incognita* in sandy soil. *Braz J Microbiol* **42**, 105-113 (2011).

16 Akhtar, M. S. & Siddiqui, Z. A. *Glomus intraradices*, *Pseudomonas alcaligenes*, and *Bacillus pumilus*: effective agents for the control of root-rot disease complex of chickpea (*Cicer arietinum* L.). *J Gen Plant Pathol* **74**, 53-60 (2007).

17 Xia, Y. *et al.* The purL gene of *Bacillus subtilis* is associated with nematicidal activity. *FEMS Microbiol Lett* **322**, 99-107 (2011).

18 Joshi, S. & Satyanarayana, T. Characteristics and applications of a recombinant alkaline serine protease from a novel bacterium *Bacillus lehensis*. *Bioresour Technol* **131**, 76-85 (2013).

19 Terefe, M., Tefera, T. & Sakhuja, P. K. Effect of a formulation of *Bacillus firmus* on root-knot nematode *Meloidogyne incognita* infestation and the growth of tomato plants in the greenhouse and nursery. *J Invertebr Pathol* **100**, 94-99 (2009).

20 Rumbaugh, K. P. Fatal attraction bacterial bait lures worms to their death. *Proc Natl Acad Sci U S A* **107**, 16411-16412 (2010).

21 Niu, Q. *et al.* A Trojan horse mechanism of bacterial pathogenesis against nematodes. *Proc Natl Acad Sci U S A* **107**, 16631-16636 (2010).

22 Huang, X. W., Niu, Q. H., Zhou, W. & Zhang, K. Q. *Bacillus nematocida* sp. nov., a novel bacterial strain with nematotoxic activity isolated from soil in Yunnan, China. *Syst Appl Microbiol* **28**, 323-327 (2005).

23 Yang, L. L. *et al.* *Lysinibacillus mangiferahumi* sp. nov., a new bacterium producing nematicidal volatiles. *Antonie van Leeuwenhoek* **102**, 53-59 (2012).

24 Li, B., Xie, G. L., Soad, A. & Coosemans, J. Suppression of *Meloidogyne javanica* by antagonistic and plant growth-promoting rhizobacteria. *J Zhejiang Univ Sci B* **6**, 496-501 (2005).

25 Tian, B., Huang, W., Huang, J., Jiang, X. & Qin, L. Investigation of protease-mediated cuticle-degradation of nematodes by using an improved immunofluorescence-localization method. *J Invertebr Pathol* **101**, 143-146 (2009).

26 Son, S. H., Khan, Z., Kim, S. G. & Kim, Y. H. Plant growth-promoting rhizobacteria, *Paenibacillus polymyxa* and *Paenibacillus lentimorbus* suppress disease complex caused by root-knot nematode and fusarium wilt fungus. *J Appl Microbiol* **107**, 524-532 (2009).

27 Akhtar, M. S. & Panwar, J. Efficacy of root-associated fungi and PGPR on the growth of *Pisum sativum*(cv. Arkil) and reproduction of the root-knot nematode *Meloidogyne incognita*. *J Basic Microbiol* **53**, 318-326 (2013).

28 Khan, Z. *et al.* A plant growth promoting rhizobacterium, *Paenibacillus polymyxa* strain GBR-1, suppresses root-knot nematode. *Bioresour Technol* **99**, 3016-3023 (2008).

29 Enright, M. R. Characterization of endospore-forming bacteria associated with entomopathogenic nematodes, *Heterorhabditis* spp., and description of *Paenibacillus nematophilus* sp. nov. *Int J Syst Evol Microbiol* **53**, 435-441 (2003).

30 Enright, M. R. & Griffin, C. T. Specificity of association between *Paenibacillus* spp. and the entomopathogenic nematodes, *Heterorhabditis* spp. *Microb Ecol* **48**, 414-423 (2004).

31 Enright, M. R. & Griffin, C. T. Effects of *Paenibacillus nematophilus* on the entomopathogenic nematode *Heterorhabditis megidis*. *J Invertebr Pathol* **88**, 40-48 (2005).

32 Kloepper, J. W., Rodríguez-Kábana, R., McInroy, J. A. & Young, R. W. Rhizosphere bacteria antagonistic to soybean cyst (*Heterodera glycines*) and root-knot (*Meloidogyne incognita*) nematodes: identification by fatty acid analysis and frequency of biological control activity. *Plant Soil* **139**, 75-84 (1992).

33 Noel, G. R. Emended description of *Pasteuria nishizawae*. *Int J Syst Evol Microbiol* **55**, 1681-1685 (2005).

34 Mohan, S., Mauchline, T. H., Rowe, J., Hirsch, P. R. & Davies, K. G. *Pasteuria* endospores from *Heterodera cajani* (Nematoda: Heteroderidae) exhibit inverted attachment and altered germination in cross-infection studies with *Globodera pallida* (Nematoda: Heteroderidae). *FEMS Microbiol Ecol* **79**, 675-684 (2012).

35 Bishop, A. H., Gowen, S. R., Pembroke, B. & Trotter, J. R. Morphological and molecular characteristics of a new species of *Pasteuria* parasitic on *Meloidogyne ardenensis*. *J Invertebr Pathol* **96**, 28-33 (2007).

36 Starr, M. P. & Sayre, R. M. *Pasteuria thornei* sp. nov. and *Pasteuria penetrans* *sensu stricto* emend., mycelial and endospore-forming bacteria parasitic, respectively, on plant-parasitic nematodes of the genera *Pratylenchus* and *Meloidogyne*. *Ann Inst Pasteur Microbiol* **139**, 11-31 (1988).

37 Schmidt, L. M., Mouton, L., Nong, G., Ebert, D. & Preston, J. F. Genetic and immunological comparison of the cladoceran parasite *Pasteuria ramosa* with the nematode parasite *Pasteuria penetrans*. *Appl Environ Microbiol* **74**, 259-264 (2007).

38 Giblin-Davis, R. M. *et al.* '*Candidatus* Pasteuria aldrichii', an obligate endoparasite of the bacterivorous nematode *Bursilla*. *Int J Syst Evol Microbiol* **61**, 2073-2080 (2011).

39 Giblin-Davis, R. M. *et al.* '*Candidatus* pasteuria usgae' sp. nov., an obligate endoparasite of the phytoparasitic nematode *Belonolaimus longicaudatus*. *Int J Syst Evol Microbiol* **53**, 197-200 (2003).

40 Mohan, S., Mauchline, T. H., Rowe, J., Hirsch, P. R. & Davies, K. G. *Pasteuria* endospores from *Heterodera cajani* (Nematoda: Heteroderidae) exhibit inverted attachment and altered germination in cross-infection studies with *Globodera pallida* (Nematoda: Heteroderidae). *FEMS Microbiol Ecol* **79**, 675-684 (2012).

**Table S2** Nematicidal activities of the 120 spore-forming Bacilli

| strains | taxa | nematodes mortality at diluted culture of c/10 | nematodes mortality at diluted culture of c/100 |
| --- | --- | --- | --- |
| G25-32 | *B. amyloliquefaciens* | 0.618 | 0.625 |
| G25-110 | *B. amyloliquefaciens* | 0.367 | 0.016 |
| G25-109 | *B. aryabhattai* | 0.425 | 0.1569 |
| G25-122 | *B. atrophaeus* | 0.5938 | 0.6983 |
| G25-86 | *B. cereus* | 0.8495 | 0.688 |
| G25-106 | *B. cereus* | 0.359 | 0.199 |
| G25-65 | *B. cereus* | 0.729 | 0.376 |
| G25-66 | *B. cereus* | 0.666 | 0.412 |
| G25-77 | *B. cereus* | 0.1055 | 0.1837 |
| G25-135-1 | *B. endophyticus* | 0.2962 | 0.1485 |
| G25-55 | *B. firmus* | 0.722 | 0.231 |
| G25-56 | *B. firmus* | 0.702 | 0.23 |
| G25-27 | *B. firmus* | 0.561 | 0.419 |
| G25-69 | *B. firmus* | 0.458 | 0.105 |
| G25-58 | *B. firmus* | 0.449 | 0.106 |
| G25-70 | *B. firmus* | 0.44 | 0.124 |
| G25-72 | *B. firmus* | 0.392 | 0.101 |
| G25-67 | *B. firmus* | 0.378 | 0.007 |
| G25-73 | *B. firmus* | 0.294 | 0.094 |
| G25-26 | *B. firmus* | 0.429 | 0.233 |
| G25-74 | *B. galactosidilyticus* | 0.276 | 0.267 |
| G25-134 | *B. lehensis* | 0.4619 | 0.338 |
| G25-99 | *B. licheniformis* | 0.301 | 0.123 |
| G25-100 | *B. licheniformis* | 0.1755 | 0.205 |
| G25-136 | *B. licheniformis* | 0.6079 | 0.551 |
| G25-30 | *B. megaterium* | 0.704 | 0.588 |
| G25-68 | *B. muralis* | 0.366 | 0.147 |
| G25-102 | *B. mycoides* | 0.425 | 0.021 |
| G25-108 | *B. mycoides* | 0.308 | 0.1389 |
| G25-101 | *B. mycoides* | 0.243 | 0.0809 |
| G25-35 | *B. pumilus* | 0.687 | 0.549 |
| G25-37 | *B. pumilus* | 0.465 | 0.46 |
| G25-38 | *B. pumilus* | 0.432 | 0.45 |
| G25-63 | *B. pumilus* | 0.275 | 0.061 |
| G25-64 | *B. pumilus* | 0.188 | 0.132 |
| G25-36 | *B. pumilus* | 0.503 | 0.506 |
| G25-119 | *B. stratosphericus* | 0.183 | 0.0369 |
| G25-132-1 | *B. stratosphericus* | 0.3581 | 0.1652 |
| G25-61 | *B. subtilis* | 0.409 | 0.523 |
| G25-40 | *B. subtilis* | 0.663 | 0.542 |
| G25-31 | *B. subtilis* | 0.56 | 0.669 |
| G25-60 | *B. subtilis* | 0.357 | 0.132 |
| G25-90 | *B. subtilis* | 0.4259 | 0.2444 |
| G25-135 | *B. subtilis* | 0.588 | 0.2901 |
| G25-83 | *B. subtilis* | 0.8495 | 0.4435 |
| G25-81 | *B. thuringiensis* | 0.8045 | 0.3572 |
| G25-45 | *B. thuringiensis* | 0.848 | 0.434 |
| G25-87 | *B. thuringiensis* | 0.8325 | 0.4045 |
| G25-91 | *B. thuringiensis* | 0.7965 | 0.167 |
| G25-49 | *B. thuringiensis* | 0.778 | 0.249 |
| G25-92 | *B. thuringiensis* | 0.7705 | 0.327 |
| G25-80 | *B. thuringiensis* | 0.748 | 0.4605 |
| G25-47 | *B. thuringiensis* | 0.747 | 0.319 |
| G25-78 | *B. thuringiensis* | 0.7295 | 0.4895 |
| G25-93 | *B. thuringiensis* | 0.7055 | 0.323 |
| G25-48 | *B. thuringiensis* | 0.693 | 0.296 |
| G25-50 | *B. thuringiensis* | 0.662 | 0.469 |
| G25-89 | *B. thuringiensis* | 0.467 | 0.149 |
| G25-39 | *B. thuringiensis* | 0.664 | 0.471 |
| G25-51 | *B. thuringiensis* | 0.85 | 0.823 |
| G25-53 | *B. thuringiensis* | 0.85 | 0.735 |
| G25-46 | *B. thuringiensis* | 0.85 | 0.558 |
| G25-41 | *B. thuringiensis* | 0.85 | 0.532 |
| G25-76 | *B. thuringiensis* | 0.8495 | 0.20825 |
| G25-42 | *B. thuringiensis* | 0.848 | 0.46 |
| G25-97 | *B. thuringiensis* | 0.848 | 0.372 |
| G25-52 | *B. thuringiensis* | 0.845 | 0.745 |
| G25-43 | *B. thuringiensis* | 0.839 | 0.402 |
| G25-44 | *B. thuringiensis* | 0.828 | 0.439 |
| G25-85 | *B. thuringiensis* | 0.7665 | 0.6015 |
| G25-95 | *B. thuringiensis* | 0.6225 | 0.435 |
| G25-94 | *B. thuringiensis* | 0.558 | 0.3295 |
| G25-96 | *B. thuringiensis* | 0.242 | 0.0455 |
| G25-121 | *B. thuringiensis* | 0.172 | 0.043 |
| G25-120 | *B. thuringiensis* | 0.397 | 0.0014 |
| G25-9 | *B. thuringiensis* C15 | 0.847 | 0.802 |
| G25-11 | *B. thuringiensis* CT-43 | 0.842 | 0.344 |
| G25-1 | *B. thuringiensis* HD-1 | 0.56 | 0.312 |
| G25-2 | *B. thuringiensis* HD-2 | 0.55 | 0.462 |
| G25-98 | *B. thuringiensis* | 0.168 | 0.088 |
| G25-13 | *B. thuringiensis* T08003 | 0.833 | 0.288 |
| G25-17 | *B. thuringiensis* YBT-1518 | 0.841 | 0.46 |
| G25-8 | *B. thuringiensis* YBT-1520 | 0.529 | 0.318 |
| G25-18 | *B. thuringiensis* YBT-1532 | 0.836 | 0.342 |
| G25-3 | *B. thuringiensis* | 0.489 | 0.486 |
| G25-5 | *B. thuringiensis* | 0.625 | 0.391 |
| G25-6 | *B. thuringiensis* | 0.536 | 0.313 |
| G25-7 | *B. thuringiensis* | 0.524 | 0.375 |
| G25-4 | *B. thuringiensis* | 0.579 | 0.394 |
| G25-82 | *B. toyonensis* | 0.745 | 0.687 |
| G25-88 | *B. toyonensis* | 0.6455 | 0.3585 |
| G25-123 | *B. toyonensis* | 0.5251 | 0.5763 |
| G25-127 | *B. vallismortis* | 0.4427 | 0.4075 |
| G25-84 | *B. weihenstephanensis* | 0.769 | 0.65 |
| G25-105 | *B. weihenstephanensis* | 0.102 | 0.075 |
| G25-103 | *B. weihenstephanensis* | 0.431 | 0.026 |
| G25-104 | *B. weihenstephanensis* | 0.318 | 0.0379 |
| G25-57 | *B. agri* | 0.431 | 0.175 |
| G25-137 | *B. brevis* | 0.586 | 0.4968 |
| G25-125 | *B. brevis* | 0.5714 | 0.4118 |
| G25-131 | *B. laterosporus* | 0.6147 | 0.5454 |
| G25-130 | *B. laterosporus* | 0.6147 | 0.4277 |
| G25-128 | *B. laterosporus* | 0.5737 | 0.3525 |
| G25-129 | *B. laterosporus* | 0.4813 | 0.3908 |
| G25-54 | *F. arsenicus* | 0.721 | 0.537 |
| G25-29 | *F. phosphorivorans* | 0.359 | 0.336 |
| G25-113 | *L. fusiformis* | 0.557 | 0.036 |
| G25-115 | *L. sphaericus* | 0.572 | 0.162 |
| G25-116 | *L. sphaericus* | 0.502 | 0.151 |
| G25-114 | *L. sphaericus* | 0.374 | 0.098 |
| G25-112 | *L. sphaericus* | 0.246 | 0.0629 |
| G25-62 | *L. sphaericus* | 0.624 | 0.585 |
| G25-34 | *L. sphaericus* | 0.698 | 0.452 |
| G25-33 | *L. sphaericus* | 0.641 | 0.588 |
| G25-111 | *L. sphaericus* | 0.18 | 0.026 |
| G25-117 | *P. alvei* | 0.6095 | 0.3262 |
| G25-118 | *P. alvei* | 0.086 | 0.027 |
| G25-126 | *P. dendritiformis* | 0.5632 | 0.2392 |
| G25-75 | *P. larvae* | 0.506 | 0.239 |
| G25-124 | *P. polymyxa* | 0.6147 | 0.5861 |
| OP50 | *E. coli* | 0.03 | 0.031 |

Note: Nematicidal activity of diluted culture by 10 times (c/10) and 100 times (c/100) of 120 spore-forming Bacilli to nematodes. The concentration of origional culture is defined as c. The mortality of nematodes was the average ratio of the number of dead nematodes to the number of total nematodes of three wells of the 96-well plates in each diluted culture. The nematicidal activity of 100 times diluted culture is almost lower than that of corresponding 10 times diluted culture of each strain; however, the nematicidal activity of the latter is litter higher than the former sometimes, which may be due to systematic errors. At least 100 worms were observed for each diluted culture in one biological repeat. Three independent biological repeats were performed. Data are shown as means of three biological repeats.

**Table S3** Sequencing and assembly information of the 115 nematicidal spore-forming Bacilli genomes

| Strain information | Library information | | | | Assembly information | | | | | coverage folds |
| --- | --- | --- | --- | --- | --- | --- | --- | --- | --- | --- |
| strain name | insert size (bp) | read length (bp) | reads number (bp) | total length of reads | Number | Total size (bp) | Longest (bp) | N50 (bp) | L50 |
| G25-5 | 500 | 90;90 | 11,166,668 | 1,005,000,120 | 74 | 5876813 | 1471384 | 584728 | 4 | 171 |
| G25-7 | 500 | 90;90 | 11,222,224 | 1,010,000,160 | 425 | 5855775 | 686181 | 352879 | 6 | 172 |
| G25-26 | 380 | 150;150 | 3,680,971 | 552,145,650 | 41 | 5008741 | 2255139 | 857583 | 2 | 110 |
| G25-27 | 380 | 125;125 | 9,159,972 | 1,144,996,500 | 45 | 5091653 | 915692 | 423826 | 5 | 225 |
| G25-29 | 380 | 150;150 | 5,228,677 | 784,301,550 | 552 | 4214535 | 1884853 | 874335 | 2 | 186 |
| G25-30 | 380 | 150;150 | 4,430,206 | 664,530,900 | 111 | 5508188 | 2935418 | 2935418 | 1 | 121 |
| G25-31 | 380 | 150;150 | 3,817,541 | 572,631,150 | 74 | 4081226 | 1031596 | 541410 | 3 | 140 |
| G25-32 | 380 | 125;125 | 4,671,866 | 583,983,250 | 212 | 4159672 | 588076 | 259438 | 6 | 140 |
| G25-33 | 380 | 150;150 | 4,290,880 | 643,632,000 | 193 | 5111132 | 368712 | 110597 | 13 | 126 |
| G25-34 | 380 | 150;150 | 4,313,564 | 647,034,600 | 225 | 5159804 | 368427 | 110598 | 13 | 125 |
| G25-35 | 380 | 150;150 | 4,533,560 | 680,034,000 | 45 | 3753812 | 678742 | 323629 | 5 | 181 |
| G25-36 | 380 | 150;150 | 5,458,924 | 818,838,600 | 57 | 3751295 | 966644 | 627943 | 3 | 218 |
| G25-37 | 380 | 150;150 | 3,721,410 | 558,211,500 | 25 | 3749784 | 997587 | 991005 | 2 | 149 |
| G25-38 | 380 | 150;150 | 3,489,168 | 523,375,200 | 24 | 3746527 | 997533 | 986918 | 2 | 140 |
| G25-39 | 380 | 150;150 | 5,237,192 | 785,578,800 | 126 | 5985612 | 1159351 | 348287 | 5 | 131 |
| G25-40 | 380 | 150;150 | 4,331,559 | 649,733,850 | 77 | 4077250 | 1062459 | 1036581 | 2 | 159 |
| G25-41 | 700 | 250;250 | 1,631,228 | 407,807,000 | 548 | 6493441 | 377701 | 74503 | 20 | 63 |
| G25-42 | 700 | 250;250 | 1,796,282 | 449,070,500 | 813 | 5775750 | 165216 | 32375 | 54 | 78 |
| G25-43 | 700 | 250;250 | 1,515,310 | 378,827,500 | 1026 | 6517562 | 149588 | 34504 | 48 | 58 |
| G25-44 | 700 | 250;250 | 1,516,276 | 379,069,000 | 1457 | 6674543 | 242699 | 38951 | 47 | 57 |
| G25-45 | 380 | 150;150 | 4,650,103 | 697,515,450 | 752 | 6083624 | 321730 | 73416 | 25 | 115 |
| G25-46 | 700 | 250;250 | 1,782,584 | 445,646,000 | 275 | 6167248 | 574157 | 141475 | 11 | 72 |
| G25-47 | 380 | 150;150 | 4,434,820 | 665,223,000 | 1530 | 6713544 | 297806 | 54993 | 33 | 99 |
| G25-48 | 380 | 150;150 | 4,086,651 | 612,997,650 | 198 | 5733820 | 557259 | 135611 | 9 | 107 |
| G25-49 | 380 | 150;150 | 4,553,793 | 683,068,950 | 619 | 6620921 | 305142 | 56858 | 30 | 103 |
| G25-50 | 380 | 150;150 | 4,166,904 | 625,035,600 | 438 | 6592562 | 261296 | 72644 | 26 | 95 |
| G25-51 | 700 | 250;250 | 16,553,448 | 4,138,362,000 | 2526 | 6599731 | 246697 | 29555 | 58 | 627 |
| G25-52 | 700 | 250;250 | 1,593,424 | 398,356,000 | 806 | 5857862 | 192592 | 31824 | 50 | 68 |
| G25-53 | 700 | 250;250 | 1,373,738 | 343,434,500 | 3126 | 6347428 | 93598 | 19349 | 91 | 54 |
| G25-54 | 380 | 150;150 | 3,784,390 | 567,658,500 | 41 | 4072794 | 688866 | 270247 | 5 | 139 |
| G25-55 | 380 | 150;150 | 4,636,131 | 695,419,650 | 354 | 4868151 | 189771 | 58968 | 25 | 143 |
| G25-56 | 380 | 125;125 | 7,496,822 | 937,102,750 | 292 | 4752619 | 240314 | 62341 | 24 | 197 |
| G25-57 | 380 | 150;150 | 6,240,396 | 936,059,400 | 303 | 5639320 | 448636 | 86127 | 17 | 166 |
| G25-58 | 380 | 150;150 | 4,072,830 | 610,924,500 | 374 | 4842093 | 189771 | 62343 | 25 | 126 |
| G25-60 | 380 | 150;150 | 3,696,364 | 554,454,600 | 148 | 4225778 | 285866 | 88580 | 16 | 131 |
| G25-61 | 380 | 150;150 | 4,355,906 | 653,385,900 | 57 | 4094840 | 1064182 | 1048732 | 2 | 160 |
| G25-62 | 380 | 150;150 | 4,254,154 | 638,123,100 | 187 | 5143436 | 368593 | 105748 | 14 | 124 |
| G25-63 | 380 | 125;125 | 5,882,900 | 735,362,500 | 27 | 3751317 | 997863 | 990952 | 2 | 196 |
| G25-64 | 380 | 150;150 | 4,556,246 | 683,436,900 | 23 | 3746631 | 997245 | 986918 | 2 | 182 |
| G25-65 | 380 | 125;125 | 5,870,054 | 733,756,750 | 1733 | 6153500 | 481572 | 121539 | 14 | 119 |
| G25-66 | 380 | 150;150 | 4,669,425 | 700,413,750 | 750 | 6125584 | 481396 | 115372 | 14 | 114 |
| G25-67 | 380 | 150;150 | 4,140,695 | 621,104,250 | 290 | 4871558 | 149849 | 63888 | 25 | 127 |
| G25-68 | 380 | 125;125 | 5,665,179 | 708,147,375 | 75 | 5111352 | 636075 | 241902 | 6 | 139 |
| G25-69 | 380 | 125;125 | 5,216,962 | 652,120,250 | 56 | 5652225 | 1653995 | 447519 | 4 | 115 |
| G25-70 | 380 | 150;150 | 6,201,343 | 930,201,450 | 335 | 5055998 | 169553 | 56794 | 26 | 184 |
| G25-72 | 380 | 150;150 | 3,874,527 | 581,179,050 | 340 | 5063783 | 249039 | 56497 | 28 | 115 |
| G25-73 | 380 | 150;150 | 3,647,519 | 547,127,850 | 225 | 5006710 | 280710 | 67179 | 22 | 109 |
| G25-74 | 380 | 125;125 | 4,540,435 | 567,554,375 | 111 | 4841616 | 539227 | 155609 | 10 | 117 |
| G25-75 | 380 | 125;125 | 4,171,870 | 521,483,750 | 380 | 4556100 | 239999 | 42724 | 29 | 114 |
| G25-76 | 500 | 90;90 | 11,188,890 | 1,007,000,100 | 109 | 6365001 | 531489 | 147655 | 13 | 158 |
| G25-77 | 380 | 150;150 | 4,101,028 | 615,154,200 | 62 | 6025663 | 1032628 | 408244 | 5 | 102 |
| G25-78 | 500 | 90;90 | 11,222,224 | 1,010,000,160 | 363 | 6167090 | 133364 | 47011 | 41 | 164 |
| G25-80 | 500 | 90;90 | 11,144,446 | 1,003,000,140 | 63 | 5863107 | 1302920 | 393658 | 5 | 171 |
| G25-81 | 500 | 90;90 | 11,211,112 | 1,009,000,080 | 59 | 5764804 | 1542652 | 407695 | 4 | 175 |
| G25-82 | 380 | 150;150 | 4,401,593 | 660,238,950 | 76 | 6350931 | 1542427 | 393658 | 5 | 104 |
| G25-83 | 500 | 90;90 | 11,177,778 | 1,006,000,020 | 12 | 4102241 | 1260886 | 1048675 | 2 | 245 |
| G25-84 | 500 | 90;90 | 11,211,112 | 1,009,000,080 | 99 | 5501598 | 852770 | 352524 | 5 | 183 |
| G25-85 | 500 | 90;90 | 11,211,112 | 1,009,000,080 | 150 | 6318045 | 562617 | 147509 | 13 | 160 |
| G25-86 | 380 | 150;150 | 4,622,285 | 693,342,750 | 292 | 6602976 | 405672 | 98281 | 22 | 105 |
| G25-87 | 500 | 90;90 | 11,211,112 | 1,009,000,080 | 371 | 6180493 | 133070 | 45172 | 41 | 163 |
| G25-88 | 500 | 90;90 | 11,177,778 | 1,006,000,020 | 72 | 5925308 | 792698 | 231883 | 7 | 170 |
| G25-89 | 500 | 90;90 | 11,155,556 | 1,004,000,040 | 92 | 5916315 | 794514 | 393166 | 6 | 170 |
| G25-90 | 380 | 150;150 | 4,237,538 | 635,630,700 | 41 | 4123202 | 1050332 | 1041814 | 2 | 154 |
| G25-91 | 300 | 100;100 | 12,752,272 | 1,275,227,200 | 398 | 6088785 | 133266 | 44932 | 42 | 209 |
| G25-92 | 500 | 90;90 | 11,111,112 | 1,000,000,080 | 771 | 6482114 | 1543108 | 408244 | 4 | 154 |
| G25-93 | 380 | 150;150 | 4,473,662 | 671,049,300 | 320 | 6848754 | 532168 | 76963 | 25 | 98 |
| G25-94 | 380 | 150;150 | 4,799,319 | 719,897,850 | 51 | 6234512 | 1320184 | 639394 | 4 | 115 |
| G25-95 | 380 | 150;150 | 4,491,258 | 673,688,700 | 55 | 6232897 | 1135860 | 639394 | 4 | 108 |
| G25-96 | 380 | 150;150 | 4,422,698 | 663,404,700 | 667 | 6440658 | 224315 | 85519 | 24 | 103 |
| G25-97 | 380 | 150;150 | 4,421,204 | 663,180,600 | 297 | 6906356 | 530863 | 74636 | 25 | 96 |
| G25-98 | 380 | 150;150 | 4,509,004 | 676,350,600 | 112 | 5776135 | 1064503 | 599598 | 4 | 117 |
| G25-99 | 380 | 125;125 | 5,630,861 | 703,857,625 | 32 | 4238093 | 600358 | 496207 | 4 | 166 |
| G25-100 | 380 | 125;125 | 5,405,743 | 675,717,875 | 49 | 4426772 | 901987 | 704055 | 3 | 153 |
| G25-101 | 380 | 125;125 | 5,125,547 | 640,693,375 | 1246 | 6144521 | 481560 | 114949 | 15 | 104 |
| G25-102 | 380 | 150;150 | 4,256,942 | 638,541,300 | 241 | 6179126 | 481537 | 114874 | 15 | 103 |
| G25-103 | 380 | 150;150 | 4,242,059 | 636,308,850 | 149 | 5889643 | 898105 | 272577 | 7 | 108 |
| G25-104 | 380 | 125;125 | 5,970,424 | 746,303,000 | 52 | 5618310 | 1940029 | 1119513 | 2 | 133 |
| G25-105 | 380 | 125;125 | 5,883,404 | 735,425,500 | 95 | 6011681 | 861813 | 259496 | 6 | 122 |
| G25-106 | 380 | 150;150 | 4,069,327 | 610,399,050 | 67 | 5941956 | 1129194 | 357710 | 5 | 103 |
| G25-108 | 380 | 150;150 | 3,983,244 | 597,486,600 | 68 | 5389160 | 740533 | 224238 | 7 | 111 |
| G25-109 | 380 | 125;125 | 8,224,945 | 1,028,118,125 | 89 | 5497026 | 2794963 | 2794963 | 1 | 187 |
| G25-110 | 380 | 150;150 | 4,298,703 | 644,805,450 | 47 | 3913688 | 768994 | 342967 | 4 | 165 |
| G25-111 | 380 | 150;150 | 4,133,162 | 619,974,300 | 134 | 5135358 | 688415 | 258329 | 6 | 121 |
| G25-112 | 380 | 150;150 | 4,027,380 | 604,107,000 | 736 | 5384769 | 583900 | 208847 | 9 | 112 |
| G25-113 | 380 | 150;150 | 5,437,896 | 815,684,400 | 95 | 4633851 | 688415 | 219306 | 7 | 176 |
| G25-114 | 380 | 125;125 | 10,070,866 | 1,258,858,250 | 129 | 4765138 | 691069 | 254591 | 6 | 264 |
| G25-115 | 380 | 125;125 | 4,458,903 | 557,362,875 | 77 | 4809759 | 688413 | 258331 | 6 | 116 |
| G25-116 | 380 | 125;125 | 8,357,977 | 1,044,747,125 | 96 | 5056377 | 688414 | 255742 | 7 | 207 |
| G25-117 | 380 | 150;150 | 4,101,961 | 615,294,150 | 226 | 6417512 | 575856 | 203078 | 10 | 96 |
| G25-118 | 380 | 125;125 | 3,538,902 | 442,362,750 | 187 | 6398614 | 576435 | 227215 | 10 | 69 |
| G25-119 | 380 | 150;150 | 3,727,952 | 559,192,800 | 105 | 3585744 | 497888 | 140516 | 7 | 156 |
| G25-120 | 380 | 150;150 | 4,673,196 | 700,979,400 | 88 | 6383838 | 1237035 | 477529 | 5 | 110 |
| G25-121 | 380 | 150;150 | 4,098,476 | 614,771,400 | 107 | 6349156 | 1237023 | 401421 | 5 | 97 |
| G25-122 | 380 | 150;150 | 4,793,676 | 719,051,400 | 23 | 4189173 | 1033939 | 946454 | 3 | 172 |
| G25-123 | 380 | 150;150 | 4,101,961 | 615,294,150 | 80 | 6064145 | 1746454 | 656264 | 3 | 101 |
| G25-124 | 380 | 125;125 | 7,804,586 | 975,573,250 | 184 | 6018908 | 670121 | 329017 | 6 | 162 |
| G25-125 | 380 | 150;150 | 3,867,053 | 580,057,950 | 71 | 6415970 | 1278946 | 354914 | 6 | 90 |
| G25-126 | 380 | 150;150 | 4,101,961 | 615,294,150 | 656 | 6176031 | 165829 | 50144 | 37 | 100 |
| G25-127 | 380 | 150;150 | 3,837,591 | 575,638,650 | 109 | 4209411 | 762796 | 194531 | 6 | 137 |
| G25-128 | 380 | 150;150 | 4,012,774 | 601,916,100 | 169 | 5706636 | 515512 | 161840 | 11 | 105 |
| G25-129 | 380 | 150;150 | 4,353,426 | 653,013,900 | 190 | 5337940 | 508625 | 102116 | 14 | 122 |
| G25-130 | 380 | 150;150 | 4,232,161 | 634,824,150 | 172 | 5369898 | 243980 | 135193 | 14 | 118 |
| G25-131 | 380 | 150;150 | 3,998,950 | 599,842,500 | 752 | 5538611 | 319422 | 119379 | 16 | 108 |
| G25-132-1 | 380 | 125;125 | 5,317,125 | 664,640,625 | 581 | 3738302 | 1832280 | 938324 | 2 | 178 |
| G25-134 | 380 | 150;150 | 4,509,320 | 676,398,000 | 31 | 3975488 | 1203326 | 890587 | 2 | 170 |
| G25-135 | 380 | 150;150 | 3,824,489 | 573,673,350 | 65 | 4935707 | 1085498 | 1028919 | 3 | 116 |
| G25-135-1 | 380 | 125;125 | 5,807,895 | 725,986,875 | 87 | 5801680 | 1269235 | 338822 | 5 | 125 |
| G25-136 | 380 | 150;150 | 4,004,297 | 600,644,550 | 54 | 4514863 | 1024377 | 674344 | 3 | 133 |
| G25-137 | 380 | 150;150 | 3,506,987 | 526,048,050 | 116 | 6340578 | 669258 | 228916 | 9 | 83 |

**Table S4** The 120 spore-forming Bacilli in this study

| strains | original strain names | source | Genome size (bp) | % mol GC | No of encoded proteins | No of scaffolds (chromosome / plasmid) -status | NCBI Project ID | references to strain source |
| --- | --- | --- | --- | --- | --- | --- | --- | --- |
| Bacillales | | | | | | | | |
| Bacillaceae (family) | | | | | | | | |
| *Bacillus* (genus) | | | | | | | | |
| Bc group (group) | | | | | | | | |
| *B. thuringiensis* | | | | | | | | |
| G25-1 | HD-1, Bacillus Genetic Stock Center (BGSC) | isolated from the dead pink bollworm turned black in Texas, USA | 6766594 | 34.92 | 6583 | 14(1/13)-F | CP004870-CP004883 | [^1^](#_ENREF_1) |
| G25-2 | T01001 (HD-2), BGSC | isolated from the Mediterranean flour moth, Ephestia kuhniella | 6323123 | 34.8 | 6423 | 246-D | ACNA00000000 | [^2^](#_ENREF_2) |
| G25-3 | 4B2, BGSC | isolated from *Malacosoma distria* in the US | 5704381 | 35.26 | 5787 | 69-D | LPZU00000000 |  |
| G25-4 | T70001, BGSC | not known | 6022089 | 35.05 | 5971 | 96-D | LPZV00000000 |  |
| G25-5 | 4BL1, BGSC | soil | 5877908 | 35.26 | 5805 | 41-D | LDJQ00000000 |  |
| G25-6 | 4BZ2, BGSC | isolated from black soil in China | 6247040 | 34.95 | 6392 | 127-D | LPZW00000000 |  |
| G25-7 | 4CD1, BGSC | soil | 5865121 | 35.23 | 5932 | 79-D | LDKE00000000 |  |
| G25-8 | YBT-1520, Huazhong Agricultural University (HZAU) | soil, China | 6580536 | 34.91 | 6295 | 12(1/11)-F | CP004858-CP004869 | [^3-6^](#_ENREF_3) |
| G25-9 | C15, HZAU | soil, China | 6248802 | 35.26 | 6386 | 322-D | LPZX00000000 |  |
| G25-11 | CT-43, HZAU | soil, China | 6151150 | 35.12 | 5998 | 11(1/10)-F | CP001907-CP001917 | [^7^](#_ENREF_7) |
| G25-13 | T08003, BGSC | not known | 6200456 | 35.29 | 6350 | 408-D | LPZY00000000 |  |
| G25-17 | YBT-1518, HZAU | soil, China | 6672921 | 35.29 | 6394 | 7(1/6)-F | CP005935-CP005940,CP002486 | [^8^](#_ENREF_8) |
| G25-18 | YBT-1532, HZAU | soil, China | 5839535 | 34.85 | 6002 | 354-D | LPZZ00000000 |  |
| G25-39 | CGMCC1.932, China General Microbiological Culture Collection Center (CGMCC) | prevent and control nematode of peanut, China | 5985484 | 34.86 | 5945 | 53-D | LDFI00000000 |  |
| G25-41 | CGMCC1.934, CGMCC | not known | 6516411 | 35.17 | 6591 | 340-D | LDFJ00000000 |  |
| G25-42 | B-18244, Agricultural Research Service Culture Collection (NRRL) | not known | 5793908 | 35.31 | 6052 | 408-D | LDER00000000 |  |
| G25-43 | B-18245, NRRL | not known | 6549982 | 35.14 | 6636 | 576-D | LDFL00000000 |  |
| G25-44 | B-18246, NRRL | not known | 6761087 | 35.35 | 6788 | 854-D | LDEQ00000000 |  |
| G25-45 | B-18247, NRRL | not known | 6084545 | 35.15 | 6123 | 234-D | LDFM00000000 |  |
| G25-46 | B-18679, NRRL | not known | 6178760 | 35.27 | 6301 | 176-D | LDFN00000000 |  |
| G25-47 | B-18680, NRRL | not known | 6715840 | 35.24 | 6756 | 274-D | LDFO00000000 |  |
| G25-48 | B-18681, NRRL | not known | 5735183 | 35.18 | 5713 | 102-D | LDFP00000000 |  |
| G25-49 | B-18682, NRRL | not known | 6623642 | 35.12 | 6842 | 320-D | LDFQ00000000 |  |
| G25-50 | B-18683, NRRL | not known | 6595079 | 35.23 | 6709 | 265-D | LDFR00000000 |  |
| G25-51 | B-18684, NRRL | not known | 6652178 | 35.09 | 6776 | 680-D | LDFS00000000 |  |
| G25-52 | B-18685, NRRL | not known | 5886148 | 35.34 | 6167 | 391-D | LDFT00000000 |  |
| G25-53 | B-18686, NRRL | not known | 6786941 | 35.21 | 6646 | 2390-D | LDFU00000000 |  |
| G25-76 | GS16, from Ralf J. Sommer, Sommerlab of Max-Planck Institute for Developmental Biology | soil, Germany | 6367219 | 35 | 6335 | 91-D | LDGN00000000 | [^9^](#_ENREF_9) |
| G25-78 | GS98, from Ralf J. Sommer (Sommerlab) | soil, Germany | 6170380 | 35.25 | 6375 | 263-D | LDGP00000000 | [^9^](#_ENREF_9) |
| G25-80 | GS127, from Ralf J. Sommer (Sommerlab) | soil, Germany | 5863811 | 35.23 | 5814 | 41-D | LDGQ00000000 | [^9^](#_ENREF_9) |
| G25-81 | GS130, from Ralf J. Sommer (Sommerlab) | soil, Germany | 5765702 | 35.29 | 5695 | 38-D | LDGR00000000 | [^9^](#_ENREF_9) |
| G25-85 | D60, from Ralf J. Sommer (Sommerlab) | horse dung | 6320356 | 35.04 | 6282 | 100-D | LDGU00000000 | [^9^](#_ENREF_9) |
| G25-87 | D149, from Ralf J. Sommer (Sommerlab) | horse dung | 6182425 | 35.24 | 6375 | 261-D | LDGW00000000 | [^9^](#_ENREF_9) |
| G25-89 | DB31, from Ralf J. Sommer (Sommerlab) | dung beetles | 5916898 | 35.19 | 5859 | 42-D | LDGY00000000 | [^9^](#_ENREF_9) |
| G25-91 | DB106, from Ralf J. Sommer (Sommerlab) | dung beetles | 6089428 | 35.2 | 6242 | 297-D | LDJT00000000 | [^9^](#_ENREF_9) |
| G25-92 | DB158, from Ralf J. Sommer (Sommerlab) | dung beetles | 6565196 | 38.68 | 6333 | 692-D | LDHA00000000 | [^9^](#_ENREF_9) |
| G25-93 | IBL-00122, from Phyllis A.W. Martin, Invasive  Insect Biocontrol and Behavior Laboratory (IIBBL) | soil | 6849847 | 35.05 | 6866 | 207-D | LDHB00000000 | [^10^](#_ENREF_10) |
| G25-94 | IBL-00169, from Phyllis A.W. Martin (IIBBL) | soil | 6234579 | 34.9 | 6205 | 37-D | LDHC00000000 |  |
| G25-95 | IBL-00423, from Phyllis A.W. Martin (IIBBL) | soil | 6233164 | 34.89 | 6206 | 36-D | LDHD00000000 |  |
| G25-96 | IBL-03087, from Phyllis A.W. Martin (IIBBL) | soil | 6441491 | 34.91 | 6575 | 207-D | LDHE00000000 |  |
| G25-97 | IBL-03090, from Phyllis A.W. Martin (IIBBL) | soil | 6907172 | 35 | 6918 | 208-D | LDHF00000000 | [^10^](#_ENREF_10) |
| G25-98 | 4BM1, BGSC | isolated from soil in Spain | 5776367 | 34.97 | 5618 | 62-D | LDHG00000000 |  |
| G25-120 | 33A4, BGSC | not known | 6383818 | 34.81 | 6295 | 58-D | LDIA00000000 |  |
| G25-121 | 40A7, BGSC | not known | 6349221 | 34.8 | 6238 | 60-D | LDIB00000000 |  |
| *B. cereus* | | | | | | | | |
| G25-65 | 29A1, BGSC | not known | 6154277 | 35.21 | 6338 | 111-D | LDGF00000000 |  |
| G25-66 | 29A2, BGSC | not known | 6126351 | 35.2 | 6316 | 110-D | LDGG00000000 |  |
| G25-77 | GS21, from Ralf J. Sommer (Sommerlab) | soil, Germany | 6026025 | 35.23 | 6007 | 42-D | LDGO00000000 | [^9^](#_ENREF_9) |
| G25-86 | D112, from Ralf J. Sommer (Sommerlab) | horse dung | 6603078 | 35.26 | 6700 | 200-D | LDGV00000000 | [^9^](#_ENREF_9) |
| G25-106 | 6A48, BGSC | isolated from kurkuma root, Thailand | 5941878 | 34.96 | 5823 | 44-D | LDHN00000000 |  |
| *B. weihenstephanensis* | | | | | | | | |
| G25-84 | D5, from Ralf J. Sommer (Sommerlab) | horse dung | 5502465 | 35.42 | 5566 | 47-D | LDGT00000000 | [^9^](#_ENREF_9) |
| G25-103 | 6A24, BGSC | isolated from soil, Germany; psychrotolerant | 5896147 | 35.15 | 5921 | 106-D | LDHK00000000 |  |
| G25-104 | 6A46, BGSC | isolated from pasteurized milk, Germany | 5618515 | 35.47 | 5541 | 37-D | LDHL00000000 |  |
| G25-105 | 6A47, BGSC | isolated from pasteurized milk, Germany | 6011619 | 35.37 | 5909 | 74-D | LDHM00000000 |  |
| ***B. toyonensis*** | | | | | | | | |
| **G25-82** | GS158, from Ralf J. Sommer (Sommerlab) | soil, Germany | 6350992 | 35.11 | 6351 | 39-D | LDGS00000000 | [^9^](#_ENREF_9) |
| **G25-88** | DB16, from Ralf J. Sommer (Sommerlab) | dung beetles | 5926648 | 35.19 | 5871 | 62-D | LDGX00000000 | [^9^](#_ENREF_9) |
| **G25-123** | 16A2, BGSC | isolated from dead mosquito larvae isolated in Rangoon, Burma | 6064135 | 35.05 | 5950 | 50-D | LDID00000000 |  |
| *B. mycoides* | | | | | | | | |
| G25-101 | 6A11, BGSC | from non-potable water tank in vicinity of livestock | 6146209 | 35.2 | 6333 | 117-D | LDOO00000000 |  |
| G25-102 | 6A12, BGSC | from soil sample, United Kingdom | 6179205 | 35.25 | 6349 | 113-D | LDHJ00000000 |  |
| G25-108 | 6A68, BGSC | isolated from soil, Scotland | 5389352 | 35.32 | 5389 | 50-D | LDHO00000000 |  |
| Bs group | | | | | | | | |
| *B. amyloliquefaciens* | | | | | | | | |
| G25-32 | CGMCC1.936, CGMCC | prevent and control nematode of peanut, China | 4162272 | 46.04 | 4045 | 47-D | LDFB00000000 |  |
| G25-110 | 10A6, BGSC | isolated from plant pathogen infested soil | 3913845 | 46.46 | 3733 | 28-D | LDHQ00000000 |  |
| *B. subtilis* | | | | | | | | |
| G25-83 | GS178, from Ralf J. Sommer (Sommerlab) | soil, Germany | 4149389 | 43.54 | 4240 | 20-D | JNCJ00000000 | [^9^](#_ENREF_9) |
| G25-31 | CGMCC1.933, CGMCC | prevent and control nematode of peanut, China | 4081556 | 43.73 | 4012 | 27-D | LDFA00000000 |  |
| G25-40 | CGMCC1.934, CGMCC | prevent and control nematode of peanut, China | 4077468 | 43.72 | 4012 | 22-D | LDFK00000000 |  |
| G25-60 | ACCC10632, Agricultural Culture Collection of China (ACCC) | prevent and control nematode of peanut, China | 4226892 | 45.94 | 4426 | 37-D | LDGA00000000 |  |
| G25-61 | ACCC11088, ACCC | prevent and control nematode of peanut, China | 4094982 | 43.74 | 4017 | 20-D | LDGB00000000 |  |
| G25-90 | DB35, from Ralf J. Sommer (Sommerlab) | dung beetles | 4123283 | 43.46 | 4240 | 23-D | LDGZ00000000 | [^9^](#_ENREF_9) |
| G25-135 | ACCC05696, ACCC | soil | 4936195 | 43.6 | 4937 | 20-D | LDIN00000000 |  |
| *B. licheniformis* | | | | | | | | |
| G25-99 | 5A36, BGSC | not known | 4238094 | 46.18 | 4302 | 25-D | LDHH00000000 |  |
| G25-100 | 5A37, BGSC | isolated from the surface of the marine alga *Palmaria palmata* | 4426892 | 45.94 | 4357 | 37-D | LDHI00000000 |  |
| G25-136 | CGMCC1.10580, CGMCC | microbial inoculant | 4515151 | 45.58 | 4422 | 22-D | LDIP00000000 |  |
| *B. atrophaeus* | | | | | | | | |
| G25-122 | 11A3, BGSC | not known | 4189175 | 43.26 | 4102 | 20-D | LDIC00000000 |  |
| *B. vallismortis* | | | | | | | | |
| G25-127 | 28A4, BGSC | isolated from a sand dune with mesquite tree in Death Valley National Monumentt, CA (USA) | 4210876 | 43.73 | 4298 | 55-D | LDIH00000000 |  |
| other *Bacillus* sp. | | | | | | | | |
| *B. pumilus* | | | | | | | | |
| G25-35 | CGMCC1.938, CGMCC | prevent and control nematode of peanut, China | 3753934 | 41.63 | 3732 | 22-D | LDFE00000000 |  |
| G25-36 | CGMCC1.940, CGMCC | prevent and control nematode of peanut, China | 3751598 | 41.55 | 3734 | 23-D | LDFF00000000 |  |
| G25-37 | CGMCC1.937, CGMCC | prevent and control nematode of peanut, China | 3749721 | 41.7 | 3716 | 12-D | LDFG00000000 |  |
| G25-38 | CGMCC1.941, CGMCC | prevent and control nematode of peanut, China | 3746512 | 41.71 | 3707 | 12-D | LDFH00000000 |  |
| G25-63 | ACCC11083, ACCC | prevent and control nematode of peanut, China | 3751433 | 41.71 | 3716 | 12-D | LDGD00000000 |  |
| G25-64 | ACCC11084, ACCC | prevent and control nematode of peanut, China | 3746618 | 41.71 | 3708 | 12-D | LDGE00000000 |  |
| *B. firmus* | | | | | | | | |
| G25-26 | ACCC01149, ACCC | soil | 5009386 | 42.01 | 4924 | 17-D | LDEW00000000 |  |
| G25-27 | ACCC01126, ACCC | soil | 5092171 | 41.98 | 4970 | 34-D | LDEX00000000 |  |
| G25-55 | NCIMB8162, NCIMB Culture Collection | not known | 4868879 | 41.56 | 4929 | 189-D | LDFW00000000 |  |
| G25-56 | NCIMB9366, NCIMB Culture Collection | not known | 4753450 | 41.64 | 4792 | 164-D | LDFX00000000 |  |
| G25-58 | NCIMB701147, NCIMB Culture Collection | not known | 4847659 | 41.61 | 4873 | 204-D | LDFZ00000000 |  |
| G25-67 | BCRC11938, Bioresource Collection and Research Center (BCRC) | not known | 4872036 | 41.58 | 4917 | 173-D | LDGH00000000 |  |
| G25-69 | BCRC11940, BCRC | not known | 5652295 | 41.37 | 5610 | 33-D | LDGK00000000 |  |
| G25-70 | BCRC11941, BCRC | not known | 5056070 | 41.67 | 5162 | 197-D | LDGJ00000000 |  |
| G25-72 | BCRC11944, BCRC | not known | 5063730 | 41.69 | 5217 | 207-D | LDGL00000000 |  |
| G25-73 | BCRC11946, BCRC | not known | 5006727 | 41.85 | 5099 | 158-D | LDGM00000000 |  |
| *B. megaterium* | | | | | | | | |
| G25-30 | CGMCC1.914, CGMCC | prevent and control nematode of peanut, China | 5509502 | 37.8 | 5665 | 42-D | LDEZ00000000 |  |
| *B. aryabhattai* | | | | | | | | |
| G25-109 | 7A16, BGSC | not known | 5497028 | 37.85 | 5592 | 35-D | LDHP00000000 |  |
| *B. lehensis* | | | | | | | | |
| G25-134 | ACCC05680, ACCC | soil | 3975583 | 39.91 | 4031 | 14-D | LDIM00000000 |  |
| ***B. stratosphericus*** | | | | | | | | |
| **G25-119** | 33A3, BGSC | isolated from dead mosquito larvae obtained from Rangoon, Burma | 3586955 | 41.57 | 3579 | 48-D | LDHZ00000000 |  |
| **G25-132-1** | ACCC06489, ACCC | Pit mud (lower) | 3738947 | 41.57 | 3780 | 16-D | LDJZ00000000 |  |
| *B. endophyticus* | | | | | | | | |
| G25-135-1 | ACCC02072, ACCC | soil | 5801706 | 36.52 | 5810 | 65-D | LDIO00000000 |  |
| ***B. muralis*** | | | | | | | | |
| **G25-68** | BCRC11939, BCRC | not known | 5112474 | 42.18 | 5028 | 53-D | LDGI00000000 |  |
| ***B. galactosidilyticus*** | | | | | | | | |
| **G25-74** | BCRC12020, BCRC | not known | 4841551 | 37.58 | 4486 | 62-D | LDJR00000000 |  |
| *Fictibacillus* (genus) | | | | | | | | |
| ***F. phosphorivorans*** | | | | | | | | |
| **G25-29** | CGMCC1.1217, CGMCC | prevent and control nematode of peanut, China | 4214891 | 39.29 | 4297 | 16-D | LDEY00000000 |  |
| ***F. arsenicus*** | | | | | | | | |
| **G25-54** | NCIMB1047, NCIMB Culture Collection | a submerged sand bank | 4072794 | 39.65 | 4113 | 31-D | LDFV00000000 |  |
| *Lysinibacillus* (genus) | | | | | | | | |
| *L. sphaericus* | | | | | | | | |
| G25-33 | CGMCC1.929, CGMCC | prevent and control nematode of peanut, China | 5111484 | 36.88 | 4770 | 95-D | LDFC00000000 |  |
| G25-34 | CGMCC1.930, CGMCC | prevent and control nematode of peanut, China | 5161194 | 36.81 | 4898 | 98-D | LDFD00000000 |  |
| G25-62 | ACCC11081, ACCC | prevent and control nematode of peanut, China | 5144260 | 36.84 | 4836 | 96-D | LDGC00000000 |  |
| G25-111 | 13A1, BGSC | isolated from larvae of *Culex fatigans* in Indonesia | 5137477 | 37.38 | 5021 | 71-D | LDHR00000000 |  |
| G25-112 | 13A102, BGSC | isolated from temporary pool, Ghana | 5394109 | 36.66 | 5453 | 116-D | LDHS00000000 |  |
| G25-114 | 13A79, BGSC | isolated from diseased *Culex fatigans* larvae in Mumbai, India | 4765476 | 37.13 | 4658 | 42-D | LDHU00000000 |  |
| G25-115 | 13A82, BGSC | isolated from newly emerged adult *Simulium damnosum* from Kaduna River, Northern Nigeria | 4809780 | 37.09 | 4670 | 42-D | LDHV00000000 |  |
| G25-116 | 13A91, BGSC | isolated from soil, Scotland | 5056643 | 36.83 | 4963 | 47-D | LDHW00000000 |  |
| *L. fusiformis* | | | | | | | | |
| G25-113 | 13A108, BGSC | isolated in Alaska, USA | 4634076 | 37.21 | 4469 | 52-D | LDHT00000000 |  |
| Paenbacillaceae (family) | | | | | | | | |
| *Paenibacillus* (genus) | | | | | | | | |
| ***P. larvae*** | | | | | | | | |
| **G25-75** | BCRC12136, BCRC | not known | 4556540 | 44.07 | 4738 | 256-D | LDJS00000000 |  |
| ***P. alvei*** | | | | | | | | |
| **G25-117** | 33A1, BGSC | isolated from dead mosquito larvae obtained from Delhi, India | 6419747 | 46.42 | 5747 | 94-D | LDHX00000000 |  |
| **G25-118** | 33A2, BGSC | isolated from dead mosquito larvae obtained from Delhi, India | 6399994 | 46.43 | 5702 | 107-D | LDHY00000000 |  |
| ***P. dendritiformis*** | | | | | | | | |
| **G25-126** | 26A5, BGSC | not known | 6183834 | 54.53 | 5549 | 298-D | LDIG00000000 |  |
| *P. polymyxa* | | | | | | | | |
| G25-124 | 25A2^T^, BGSC | not known | 6021278 | 44.99 | 5494 | 79-D | LDIE00000000 |  |
| *Brevibacillus* (genus) | | | | | | | | |
| *B. laterosporus* | | | | | | | | |
| G25-128 | 40A4, BGSC | not known | 5709498 | 40.11 | 5307 | 95-D | LDII00000000 |  |
| G25-129 | 40A8, BGSC | not known | 5339979 | 40.36 | 4759 | 98-D | LDIJ00000000 |  |
| G25-130 | 40A9, BGSC | not known | 5372514 | 40.25 | 4749 | 90-D | LDIK00000000 |  |
| G25-131 | 40A10, BGSC | not known | 5540684 | 40.27 | 5076 | 100-D | LDIL00000000 |  |
| *B. brevis* | | | | | | | | |
| G25-125 | 26A3, BGSC | not known | 6415924 | 47.31 | 6009 | 42-D | LDIF00000000 |  |
| G25-137 | CGMCC1.931, CGMCC | prevent and control nematode of peanut, China | 6341629 | 47.12 | 6028 | 61-D | LDJY00000000 |  |
| ***B. agri*** | | | | | | | | |
| **G25-57** | NCIMB13289, NCIMB Culture Collection | methane fermentor at 45°C | 5640734 | 53.47 | 5463 | 155-D | LDFY00000000 |  |

1. Strains in bold are nematicidal species that are determined nematicidal newly in this study (Table S1).

2. Some *B. thuringiensis* strains have other names as following: G25-1 (*B. thuringiensis* serovar *kurstaki* str. HD-1), G25-2 (*B. thuringiensis* serovar *thuringiensis* str. T01001), G25-3 (*B. thuringiensis* serovar *finitimus* str. 4B2), G25-4 (*B. thuringiensis* serovar *sinensis* str. T70001), G25-5 (*B. thuringiensis* serovar *muju* str. 4BL1), G25-6 (*B. thuringiensis* serovar *zhaodongensis* str. 4BZ1), G25-7 (*B. thuringiensis* serovar *graciosensis* 4CD1), G25-8 (*B. thuringiensis* serovar *kurstaki* str. YBT-1520), G25-9 (*B. thuringiensis* C15), G25-11 (*B. thuringiensis* serovar *chinensis* CT-43), G25-13 (*B. thuringiensis* T08003), G25-17 (*B. thuringiensis* YBT-1518), G25-18 (*B. thuringiensis* YBT-1532). *B. thuringiensis* G25-8, G25-9, G25-11, G25-17 and G25-18 are from our team.

3. D and F represent status of genomes are draft and finished, respectively.

References

1 Johnson, S. L. *et al.* Complete genome sequences for 35 biothreat assay-relevant *Bacillus* species. *Genome Announc* **3** (2015).

2 Zwick, M. E. *et al.* Genomic characterization of the *Bacillus cereus sensu lato* species: Backdrop to the evolution of *Bacillus anthracis*. *Genome Res* **22**, 1512-1524 (2012).

3 Liu, X.. *et al.* Distribution of 2-kb miniplasmid pBMB2062 from *Bacillus thuringiensis kurstaki* YBT-1520 strain in *Bacillus* species. *Ann Microbiol* **63**, 1639-1644 (2013).

4 Zhang, Q., Sun, M., Xu, Z. & Yu, Z. Cloning and characterization of pBMB9741, a native plasmid of *Bacillus thuringiensis* subsp. *kurstaki* strain YBT-1520. *Curr Microbiol* **55**, 302-307 (2007).

5 Chao, L. *et al.* Complete nucleotide sequence of pBMB67, a 67-kb plasmid from *Bacillus thuringiensis* strain YBT-1520. *Plasmid* **57**, 44-54 (2007).

6 Liu, X. *et al.* Genetic characterization of two putative toxin-antitoxin systems on cryptic plasmids from *Bacillus thuringiensis* strain YBT-1520. *J Microbiol Biotechnol* **18**, 1630-1633 (2008).

7 He, J. *et al.* Complete genome sequence of *Bacillus thuringiensis* subsp. *chinensis* strain CT-43. *J Bacteriol* **193**, 3407-3408 (2011).

8 Wang, P. *et al.* Complete genome sequence of *Bacillus thuringiensis* YBT-1518, a typical strain with high toxicity to nematodes. *J Biotechnol* **171**, 1-2 (2014).

9 Rae, R., Iatsenko, I., Witte, H. & Sommer, R. J. A subset of naturally isolated *Bacillus* strains show extreme virulence to the free-living nematodes *Caenorhabditis elegans* and *Pristionchus pacificus*. *Environ Microbiol* **12**, 3007-3021 (2010).

10 Blackburn, M. B., Martin, P. A., Kuhar, D., Farrar, R. R., Jr. & Gundersen-Rindal, D. E. The occurrence of photorhabdus-like toxin complexes in *Bacillus thuringiensis*. *PloS One* **6**, e18122 (2011).

**Table S5** Survey of known virulence factors

| proteins | organism | gene ID | nematode target | effects on nematodes | references |
| --- | --- | --- | --- | --- | --- |
| **Proteases** | | | | | |
| **Fungal proteases** | | | | | |
| **Serine proteases** | | | | | |
| alkaline serine protease Hasp | *Hirsutella rhossiliensis* | ABD96101.1 | *Panagrellus redivivus* | Hasp killed the juveniles of the soybean-cyst nematode (*Heterodera glycines*5 and degraded proteins of the nematode cuticle | [^1^](#_ENREF_1) |
| alkaline serine protease VCP1 | *Pochonia chlamydosporia* | CAD20578.1 | *Meloidogyne incognita* | The purified enzyme hydrolysed proteins in situ from the outer layer of the egg shell of the host nematode *M. incognita* and exposed its chitin layer |  |
| alkaline serine protease P32 | *P. rubescens* | ADK74313.2 | *Globodera pallida* | The purified protease was able to degrade certain cyst nematode proteins, involving nematode egg penetration by *Verticillium suchlasporium* |  |
| alkaline serine protease Ver112 | *Lecanicillium psalliotae* | AAU01968.1 | *P. redivivus* | The puriﬁed protease degraded nematode cuticle with 81% of a nematode being degraded after treating with Ver112 for 12 h |  |
| cuticle-degrading serine protease Ac1 | *Arthrobotrys conoides* | AAX54903.1 | *P. redivivus* | Ac1 can degrade a broad range of substrates including casein, gelatin, bovine serum albumin, collagen, and nematode cuticles, can immobilize the free-living nematode *P. redivivus* and the pine wood nematode *Bursaphelenchus xylophilus* | [^8^](#_ENREF_8) |
| cuticle-degrading serine protease PII | *A. oligospora* | CAA63841.1 | *P. redivivus* | PⅡ immobilized *P. redivivus* in bioassays and hydrolysed proteins of the purified cuticle. The enzyme hydrolysed several protein substrates including casein, bovine serum albumin and gelatin, but not native collagen |  |
| cuticle-degrading serine protease PrC | *Clonostachys rosea* | ACS66684.1 | *P. redivivus* | The puriﬁed protease could degrade a broad range of substrates including casein, gelatin and nematode cuticle |  |
| cuticle-degrading serine protease Ds1 | *Dactylella shizishanna* | ABL74286.1 | *P. redivivus* | The puriﬁed protease could degrade puriﬁed cuticle of *Penagrellus redivivus* and a broad range of protein substrates | [^13^](#_ENREF_13) |
| cuticle-degrading serine protease Dv1 | *D. varietas* | ABF72192.1 | *P. redivivus*, *Caenorhabditis elegans* | This protease could immobilize the free-living nematodes *P. redivivus* and *C. elegans* and hydrolyze the purified cuticle of *P. redivivus* | [^14^](#_ENREF_14) |
| cuticle-degrading serine protease Mc1 | *Monacrosporium cystosporum* | AAX54901.1 | *P. redivivus*, *B. xylophilus* | Mc1 could degrade a broad range of substrates including casein, gelatin, BSA (bovine serum albumin), and nematode cuticle, could immobilize the free-living nematode *P. redivivus* and the pine wood nematode *B. xylophilus* | [^15^](#_ENREF_15) |
| cuticle-degrading serine protease Mlx | *M. microscaphoides* | AAW21809.2 | *P. redivivus* | This protease could immobilize the nematode *Penagrellus redivivus* in vitro and degrade its purified cuticle | [^16^](#_ENREF_16) |
| neutral serine protease Aoz1 | *A. oligospora* | AAM93666.1 | *P. redivivus* | Degrade nematode cuticle and immobilize *P. redivivus* in vitro | [^17^](#_ENREF_17) |
| Protease*****(1) | *Dactylellina cionopaga* | AEP02888.1 |  |  | unpublished |
| serine protease Csp1 | *Ophiocordyceps sinensis* | ABX79998.1 | *Hepialus* sp. | Degraded the cuticle proteins of larval *Hepialus* sp. in vitro | [^18^](#_ENREF_18) |
| serine protease Csp2 | *O. sinensis* | ABX79999.1 | *Hepialus* sp. | Degraded the cuticle proteins of larval *Hepialus* sp. in vitro | [^18^](#_ENREF_18) |
| serine protease precursor | *Purpureocillium lilacinum* | ADA70794.1 | *M. incognita* | Overexpressed enhanced the virulence of *Paecilomyces lilacinus* against *M. incognita* eggs | [^19^](#_ENREF_19) |
| subtilisin-like serine protease precursor***** (2 | *P. lilacinum* | ABO32256.1 |  |  | unpublished |
| **Bacterial proteases** | | | | | |
| alkaline serine protease Bace16 | *Bacillus nematocida* B16 | AAV30845.1 | *P. redivivus* | The purified protease can hydrolyze several native proteinaceous substrates, including collagen and nematode cuticle | [^20^](#_ENREF_20) |
| neutral protease Bae16 | *B. nematocida* B16 | AAV30844.1 | *P. redivivus* | The puriﬁed protease could destroy the nematode cuticle and its hydrolytic substrates included gelatin and collagen. | [^21^](#_ENREF_21) |
| Metalloproteinase Bmp1 | *B. thuringiensis* YBT-1518 | AFZ77001.1 | *C. elegans* | Puriﬁed Bmp1 protein showed toxicity against *C. elegans*, enhanced the toxicity of Cry5Ba and degrades intestine tissues | [^22^](#_ENREF_22) |
| Metalloproteinase ColB | *B .thuringiensis* | AHA71938.1 | *C. elegans* | Metalloproteinase ColB destroy the intestine thereby facilitates the adaptation and colonization of *B. thuringiensis* in *C. elegans*. | [^23^](#_ENREF_23) |
| cuticle-degrading protease precursor Apr219 | *Bacillus* sp. RH219 | ABI93801.1 | *P. redivivus* | Nematicidal and cuticle-degrading activities for *Bacillus* sp. RH219 were mostly due to the extracellular serine alkaline protease Apr219 | [^24^](#_ENREF_24) |
| extracellular neutral protease precursor Npr219 | *Bacillus* sp. RH219 | ABI93803.1 | *P. redivivus* | The addition of neutral protease Npr219 increased the mortality of Apr219 | [^24^](#_ENREF_24) |
| extracellular alkaline serine protease BLG4 | *Brevibacillus laterosporus* | AAU81559.2 | *P. redivivus* | The protease hydrolyzed relatively broad substrates including collagen and the cuticle of nematodes | [^25^](#_ENREF_25) |
| extracellular neutral protease precursor NPE-4 | *B. laterosporus* | ABI93802.1 | *P. redivivus* | NPE-4 could degrade proteins from the inner layer of purified cuticles from nematode *P. redivivus* in vitro. | [^26^](#_ENREF_26) |
| Metalloprotease AprA | *Pseudomonas protegens* | AAT71322.1 | *M. incognita* | AprA inhibited egg hatching and induced mortality of *M. incognita* juveniles | [^27^](#_ENREF_27) |
| metalloproteinase serralysin | *Serratia marcescens* | AFX62372.1 | *B. xylophilus* | Serralysin showed nematicidal activity to *B. xylophilus* | [^28^](#_ENREF_28) |
| serine protease precursor | *S. marcescens* | AFX62373.1 | *B. xylophilus* | Serine protease precursor (AFX62373.1) sowed nematicidal activity to *B. xylophilus* | [^28^](#_ENREF_28) |
| **Trypsin protease** | | | | | |
| trypsin-like protease PRA1 | *Trichoderma harzianum* | CAC80694.2 | *M. incognita* | Pure PRA1 preparations reduced The number of hatched eggs of the root-knot nematode *M. incognita* significantly | [^29^](#_ENREF_29) |
| **Chitinases** | | | | | |
| basic chitinase Chi2 | *V. lecanii* | AAV98691.1 |  |  | [^30^](#_ENREF_30) |
| Chitinase CrChi1 | *Clonostachys rosea* | ABV57861.1 |  | Crchi1 expression was suppressed by glucose but strongly stimulated by chitin or solubilized components of the cell wall from *Rhizoctonia solani* | [^31^](#_ENREF_31) |
| Chitinase Lpchi1 | *L. psalliotae* | ABQ57240.1 | *M. incognita* | The purified chitinase LPCHI1 was found degrading chitinous components of eggs of the root-knot nematode *M. incognita* and significantly influence its development. | [^32^](#_ENREF_32) |
| Chitinase PcChi44 | *Metacordyceps chlamydosporia* | ABW96521.1 | *M. incognita* | The chitinase PCCHI44 could damage eggs of both the root-knot nematode *M. incognita* and the insect *Bombyx mori* | [^33^](#_ENREF_33) |
| chitinase precursor PLC | *P. lilacinum* | ABP37997.1 |  | *P. lilacinum* is nematode egg-parasitic fungus | [^34^](#_ENREF_34) |
| extracellular chitinases Chi46 | *P. variotii* | AAL78814.1(  43 kDa endochitinase of *Hypocrea virens*) | *M. incognita* | *P. variotii* DG-3 is a chitinase producer and a nematode egg-parasitic fungus, the purified Chi46 showed chitinase activity bands with 0.01% glycol chitin as asubstrate | [^35^](#_ENREF_35) |
| **Other proteins** | | | | | |
| Serrawettin W2***** (3) | *S. marcescens* |  | *B. xylophilus* |  | unpublished |
| Nel | *B .thuringiensis* | AHZ54746.1 | *C. elegans* | Nel exhibited inhibition activity to nematode, and the toxicity of Nel to nematodes targets the intestine, showing that Nel triggered heat shock pathway and necrosis pathway in nematodes. | [^36^](#_ENREF_36) |
| nematicidal protein AidA | *Burkholderia cenocepacia* J2315 | YP_002153683.1 | *C. elegans* | Expression of aidA is regulated by the cep quorum sensing system. AidA is involved in killing of *C. elegans* | [^37^](#_ENREF_37) |
| sporamin A precursor | *Ipomoea batatas* | AAA33391.1 | *H. schachtii* Schm. | Sporamin-mediated resistance to beet cyst nematodes (*H. schachtii* Schm. is dependent on trypsin inhibitory activity in sugar beet(*Beta vulgaris* L. hairy roots |  |
| Lectin | *Remusatia vivipara* | ACH41914.1 | *M. incognita* | Purified RVL exerts potent nematicidal activity on *M. incognita* | [^40^](#_ENREF_40) |
| Amidophosphoribosyltransferase PurL | *B. subtilis* OKB105 | NP_388531.2(*B. subtilis* subsp. *subtilis* 168) | *Aphelenchoides besseyi*, *Ditylenchus destructor*, *B. xylophilus* and  *M. javanica* | The *purL* gene regulated the production of purine biosynthesis intermediates which affected the nematicidal activity of strain  OKB105 | [^41^](#_ENREF_41) |
| oryzastatin | *Oryza sativa* | AAA33912.1 | *G. pallida*, *Radopholus similis* | Transgenic resistance to the nematode by cystatin or oryzastatin |  |
| calcium-transporting ATPase | *Cryptococcus neoformans* var. *neoformans* JEC21 | XP_572412.1 | *C. elegans* | Eca1 is likely involved in maintaining ER function, thus contributing to stress tolerance and virulence acting in parallel with Ca^2+^-calcineurin signaling | [^44^](#_ENREF_44) |
| protein kinase kin1 | *C. neoformans* var. *neoformans* JEC21 | XP_567871.1 | *C. elegans* | The *C. neoformans* Kin1 kinase homologue is required for full virulence | [^45^](#_ENREF_45) |
| root-knot nematode resistance protein Mi-1 | *Solanum lycopersicum* | NP_001234063.1 | *Meloidogyne* spp. | Conferred Heat-Stable Resistance to Root-Knot Nematodes (is a Homolog of Mi-9) | [^46^](#_ENREF_46) |
| **peptides** | | | | | |
| shiga toxin 1 variant A subunit | *Escherichia coli* O157:H7 | BAC78639.1 | *C. elegans* | The Shiga-like toxin 1 (Stx1) of *E. coli* O157:H7 is a virulence factor in *C. elegans* and is required for full toxicity | [^47^](#_ENREF_47) |
| cycloviolacin H3 | *Viola hederacea* |  | *H. contortus* and *Trichostrongylus colubriformis* | Cycloviolacin H3 showed signiﬁcant activity in inhibiting development of nematode larvae and motility of adult worms | [^48^](#_ENREF_48) |
| cycloviolacin O1 | *V. odorata* |  | *H. contortus* and *Trichostrongylus colubriformis* | Cycloviolacin O1 showed signiﬁcant activity in inhibiting development of nematode larvae and motility of adult worms | [^48^](#_ENREF_48) |
| Cycloviolacin O2 | *V. odorata*, *V. biflora*, *V. philippica* |  | *H. contortus* and *Trichostrongylus colubriformis* | Cycloviolacin O2 showed signiﬁcant activity in inhibiting development of nematode larvae and motility of adult worms |  |
| cycloviolacin O3 | *V. odorata* |  | *H. contortus* and *Trichostrongylus colubriformis* | Cycloviolacin O3 showed signiﬁcant activity in inhibiting development of nematode larvae and motility of adult worms |  |
| cycloviolacin O8 | *V. odorata*, *V. adunca* |  | *H. contortus* and *Trichostrongylus colubriformis* | Cycloviolacin O8 showed signiﬁcant activity in inhibiting development of nematode larvae and motility of adult worms |  |
| cycloviolacin O12 | *V. tricolor*, *V. arvensis*, *V. baoshanensis*, *V. yedoensis*, *V. tianshanica*, *V. abyssinica*, *V. philippica* |  | *H. contortus* and *Trichostrongylus colubriformis* | Cycloviolacin O12 showed signiﬁcant activity in inhibiting development of nematode larvae and motility of adult worms | [^48^](#_ENREF_48) |
| cycloviolacin O13 | *V. odorata* |  | *H. contortus* and *Trichostrongylus colubriformis* | Cycloviolacin O13 showed signiﬁcant activity in inhibiting development of nematode larvae and motility of adult worms |  |
| cycloviolacin O14 | *V. odorata* |  | *Haemonchus contortus* and *Trichostrongylus colubriformis* | Cycloviolacin O14 showed signiﬁcant activity in inhibiting development of nematode larvae and motility of adult worms | [^48-50^](#_ENREF_48) |
| cycloviolacin O15 | *V. odorata* |  | *H. contortus* and *Trichostrongylus colubriformis* | Cycloviolacin O15 showed signiﬁcant activity in inhibiting development of nematode larvae and motility of adult worms |  |
| cycloviolacin O16 | *V. odorata* |  | *H. contortus* and *Trichostrongylus colubriformis* | Cycloviolacin O16 showed signiﬁcant activity in inhibiting development of nematode larvae and motility of adult worms |  |
| cycloviolacin O24 | *V. odorata* |  | *H. contortus* and *Trichostrongylus colubriformis* | Cycloviolacin O24 showed signiﬁcant activity in inhibiting development of nematode larvae and motility of adult worms | [^48^](#_ENREF_48) |
| cycloviolacin Y1 | *V. yedoensis* |  | *H. contortus* and *Trichostrongylus colubriformis* | Cycloviolacin Y1 showed signiﬁcant activity in inhibiting development of nematode larvae and motility of adult worms | [^48^](#_ENREF_48) |
| cycloviolacin Y4 | *V. yedoensis* |  | *H. contortus* and *Trichostrongylus colubriformis* | Cycloviolacin Y4 showed signiﬁcant activity in inhibiting development of nematode larvae and motility of adult worms | [^48^](#_ENREF_48) |
| cycloviolacin Y5 | *V. baoshanensis*, *V. yedoensis* |  | *H. contortus* and *Trichostrongylus colubriformis* | Cycloviolacin Y5 showed signiﬁcant activity in inhibiting development of nematode larvae and motility of adult worms | [^48^](#_ENREF_48) |
| Kalata B1 | *V. odorata*, *Oldenlandia affinis*, *V. baoshanensis*, *V. yedoensis*, *V. philippica* |  | *H. contortus* and *Trichostrongylus colubriformis* | Kalata B1 showed signiﬁcant activity in inhibiting development of nematode larvae and motility of adult worms |  |
| kalata B2 | *O. affinis* |  | *H. contortus* and *Trichostrongylus colubriformis* | Kalata B2 showed signiﬁcant activity in inhibiting development of nematode larvae and motility of adult worms |  |
| kalata B6 | *O. affinis* |  | *H. contortus* and *T. colubriformis* | Kalata B6 showed signiﬁcant activity in inhibiting development of nematode larvae and motility of adult worms |  |
| kalata B7 | *O. affinis* |  | *H. contortus* and *Trichostrongylus colubriformis* | Kalata B7 showed signiﬁcant activity in inhibiting development of nematode larvae and motility of adult worms |  |
| kalata S | *V. odorata*, *O. affinis, V. tricolor*, *V. arvensis V. Baoshanensis*, *V. Yedoensis*, *V. biflora*, *V. philippica* |  | *H. contortus* and *Trichostrongylus colubriformis* | Kalata S showed signiﬁcant activity in inhibiting development of nematode larvae and motility of adult worms | [^48^](#_ENREF_48) |
| piceain1 | *Picea sitchensis* |  | *C. elegans* | Piceain1 exerted strong nematicidal activities against *C. elegans* |  |
| piceain2 | *P. sitchensis* |  | *C. elegans* | Piceain2 exerted strong nematicidal activities against *C. elegans* |  |
| vhl-1 | *V. hederacea* |  | *H. contortus* and *Trichostrongylus colubriformis* | Vhl-1 showed signiﬁcant activity in inhibiting development of nematode larvae and motility of adult worms |  |
| **Compounds synthetic clusters** | | | | | |
| **phenazine** | | | | | |
| *phzIRABCDEFGO*  (PhzE_ADP21173.1) | *P. chlororaphis* GP72 | ADP21167.1- ADP21176.1 | *C. elegans* | Phenazines are required for fast killing of *C. elegans* |  |
| **Thuringiensin** | | | | | |
| *thuABCDEFG1234*(thu2) | *B. thuringiensis* CT-43 | CT43_P127037-CT43_P127041 | *M. incognita*, *H. glycines,* *C. elegans* and *P. pacificus* | Thuringiensin killed *M. incognita*, *H. glycines, C. elegans* and *P. pacificus* | [^56-59^](#_ENREF_56) |

***** (1) “Cloning and characterization of genes involved in preying and infection of nematode by *Dactylellina cionopaga*”

Yu, H. Y., Duan, J. X., Wang B. and Liu, X. Z.

**(2) “The basic serine protease from the opportunistic pathogen *Paecilomyces lilacinus*: implications for the microbial control of crop pests from an enzymological perspective.”**
Dong L.Q., Yang J. K. , Zhang K. Q.

(3) “**Serrawettin and extracellular proteases are involved in the nematicidal ability of bacteria associated to pinewood nematode.”**
Paiva G., Proenca D.N., Francisco R., Verissimo P., Santos S.S., Fonseca L., Abrantes I.M.O., Morais P.V.

References

1 Wang, B., Liu, X., Wu, W., Liu, X. & Li, S. Purification, characterization, and gene cloning of an alkaline serine protease from a highly virulent strain of the nematode-endoparasitic fungus *Hirsutella rhossiliensis*. *Microbiol Res* **164**, 665-673 (2009).

2 Morton, C. O., Hirsch, P. R., Peberdy, J. P. & Kerry, B. R. Cloning of and genetic variation in protease VCP1 from the nematophagous fungus *Pochonia chlamydosporia*. *Mycol Res* **107**, 38-46 (2003).

3 Segers, R., Butt, T. M., Kerry, B. R. & Peberdy, J. F. The nematophagous fungus *Verticillium chlamydosporium* produces a chymoelastase-like protease which hydrolyses host nematode proteins in situ. *Microbiology* **140**, 2715-2723 (1994).

4 Lopez-Llor, L. V. Purification and properties of extracellular proteases produced by the nematophagous fungus *Verticillium suchlasporium*. *Can J Microbiol* **36**, 530-537 (1990).

5 Larriba, E., Martín-Nieto, J. & Lopez-Llorca, L. V. Gene cloning, molecular modeling, and phylogenetics of serine protease P32 and serine carboxypeptidase SCP1 from nematophagous fungi *Pochonia rubescens* and *Pochonia chlamydosporia*. *Can J Microbiol* **58**, 815-827 (2012).

6 Yang, J. *et al.* Isolation and characterization of a serine protease from the nematophagous fungus, *Lecanicillium psalliotae*, displaying nematicidal activity. *Biotechnol Lett* **27**, 1123-1128 (2005).

7 Yang, J. *et al.* Characterization of an extracellular serine protease gene from the nematophagous fungus *Lecanicillium psalliotae*. *Biotechnol Lett* **27**, 1329-1334 (2005).

8 Yang, J. *et al.* Cloning and characterization of an extracellular serine protease from the nematode-trapping fungus *Arthrobotrys conoides*. *Arch Microbiol* **188**, 167-174 (2007).

9 Tunlid, A., Rosen, S., Ek, B. & Rask, L. Purification and characterization of an extracellular serine protease from the nematode-trapping fungus *Arthrobotrys oligospora*. *Microbiology* **140 ( Pt 7)**, 1687-1695 (1994).

10 Ahman, J., Ek, B., Rask, L. & Tunlid, A. Sequence analysis and regulation of a gene encoding a cuticle-degrading serine protease from the nematophagous fungus *Arthrobotrys oligospora*. *Microbiology* **142 ( Pt 7)**, 1605-1616 (1996).

11 Liang, L. *et al.* Cloning and homology modeling of a serine protease gene (*PrC*) from the nematophagous fungus *Clonostachys rosea*. *Ann Microbiol* **61**, 511-516 (2010).

12 Li, J., Yang, J., Huang, X. & Zhang, K.-Q. Purification and characterization of an extracellular serine protease from *Clonostachys rosea* and its potential as a pathogenic factor. *Process Biochem* **41**, 925-929 (2006).

13 Wang, R. B., Yang, J. K., Lin, C., Zhang, Y. & Zhang, K. Q. Purification and characterization of an extracellular serine protease from the nematode-trapping fungus *Dactylella shizishanna*. *Lett Appl Microbiol* **0**, 060423083226010 (2006).

14 Yang, J. *et al.* Purification and cloning of a novel serine protease from the nematode-trapping fungus *Dactylellina varietas* and its potential roles in infection against nematodes. *Appl Microbiol Biotechnol* **75**, 557-565 (2007).

15 Yang, J. K. *et al.* Purification and cloning of an extracellular serine protease from the nematode-trapping fungus *Monacrosporium cystosporium*. *J Microbiol Biotechnol* **18**, 852-858 (2008).

16 Wang, M., Yang, J. & Zhang, K.-Q. Characterization of an extracellular protease and its cDNA from the nematode-trapping fungus *Monacrosporium microscaphoides*. *Can J Microbiol* **52**, 130-139 (2006).

17 Minglian, Z., Minghe, M. & Keqin, Z. Characterization of a neutral serine protease and its full-length cDNA from the nematode-trapping fungus *Arthrobotrys oligospora*. *Mycologia* **96**, 16-22 (2004).

18 Zhang, Y., Liu, X. & Wang, M. Cloning, expression, and characterization of two novel cuticle-degrading serine proteases from the entomopathogenic fungus *Cordyceps sinensis*. *Res Microbiol* **159**, 462-469 (2008).

19 Wang, J., Wang, J., Liu, F. & Pan, C. Enhancing the virulence of *Paecilomyces lilacinus* against *Meloidogyne incognita* eggs by overexpression of a serine protease. *Biotechnol Lett* **32**, 1159-1166 (2010).

20 Niu, Q. *et al.* *Bacillus* sp. B16 kills nematodes with a serine protease identified as a pathogenic factor. *Appl Microbiol Biotechnol* **69**, 722-730 (2006).

21 Niu, Q. *et al.* A neutral protease from *Bacillus nematocida*, another potential virulence factor in the infection against nematodes. *Arch Microbiol* **185**, 439-448 (2006).

22 Luo, X. *et al.* *Bacillus thuringiensis* metalloproteinase Bmp1 functions as a nematicidal virulence factor. *Appl Environ Microbiol* **79**, 460-468 (2012).

23 Peng, D. *et al.* A novel metalloproteinase virulence factor is involved in *Bacillus thuringiensis* pathogenesis in nematodes and insects. *Environ Microbiol* (2016).

24 Lian, L. H. *et al.* Proteases from *Bacillus*: a new insight into the mechanism of action for rhizobacterial suppression of nematode populations. *Lett Appl Microbiol* **45**, 262-269 (2007).

25 Huang, X. *et al.* An extracellular protease from *Brevibacillus laterosporus* G4 without parasporal crystals can serve as a pathogenic factor in infection of nematodes. *Res Microbiol* **156**, 719-727 (2005).

26 Tian, B. *et al.* Role of an extracellular neutral protease in infection against nematodes by *Brevibacillus laterosporus* strain G4. *Appl Microbiol Biotechnol* **74**, 372-380 (2006).

27 Siddiqui, I. A., Haas, D. & Heeb, S. Extracellular protease of *Pseudomonas fluorescens* CHA0, a biocontrol factor with activity against the root-knot nematode *Meloidogyne incognita*. *Appl Environ Microbiol* **71**, 5646-5649 (2005).

28 Paiva, G. *et al.* Nematicidal bacteria associated to pinewood nematode produce extracellular proteases. *PloS One* **8**, e79705 (2013).

29 Suarez, B., Rey, M., Castillo, P., Monte, E. & Llobell, A. Isolation and characterization of PRA1, a trypsin-like protease from the biocontrol agent *Trichoderma harzianum* CECT 2413 displaying nematicidal activity. *Appl Microbiol Biotechnol* **65** (2004).

30 Lu, Z.-X., Laroche, A. & Huang, H. C. Isolation and characterization of chitinases from *Verticillium lecanii*. *Can J Microbiol* **51**, 1045-1055 (2005).

31 Gan, Z., Yang, J., Tao, N., Yu, Z. & Zhang, K. Q. Cloning and expression analysis of a chitinase gene *Crchi1* from the mycoparasitic fungus *Clonostachys rosea* (syn. *Gliocladium roseum*). *J Microbiol* **45**, 422-430 (2007).

32 Gan, Z. *et al.* Cloning of the gene *Lecanicillium psalliotae* chitinase Lpchi1 and identification of its potential role in the biocontrol of root-knot nematode *Meloidogyne incognita*. *Appl Microbiol Biotechnol* **76**, 1309-1317 (2007).

33 Mi, Q. *et al.* Cloning and overexpression of *Pochonia chlamydosporia* chitinase gene *pcchi44*, a potential virulence factor in infection against nematodes. *Process Biochem* **45**, 810-814 (2010).

34 Dong, L. Q., Yang, J. K. & Zhang, K. Q. Cloning and phylogenetic analysis of the chitinase gene from the facultative pathogen *Paecilomyces lilacinus*. *J Microbiol Biotechnol* **103**, 2476-2488 (2007).

35 Nguyen, V.-N. *et al.* Purification and characterization of chitinases from *Paecilomyces variotii* DG-3 parasitizing on *Meloidogyne incognita* eggs. *J Ind Microbiol Biotechnol* **36**, 195-203 (2008).

36 Ruan, L. *et al.* A two-domain protein triggers heat shock pathway and necrosis pathway both in model plant and nematode. *Environ Microbiol* **17**, 4547-4565 (2015).

37 Holden, M. T. *et al.* The genome of *Burkholderia cenocepacia* J2315, an epidemic pathogen of cystic fibrosis patients. *J Bacteriol* **191**, 261-277 (2009).

38 Cai, D. *et al.* Sporamin-mediated resistance to beet cyst nematodes (*Heterodera schachtii* Schm.) is dependent on trypsin inhibitory activity in sugar beet (*Beta vulgaris* L.) hairy roots. *Plant Mol Biol* **51**, 839-849 (2003).

39 Hattori, T., Nakagawa, T., Maeshima, M., Nakamura, K. & Asahi, T. Molecular cloning and nucleotide sequence of cDNA for sporamin, the major soluble protein of sweet potato tuberous roots. *Plant Mol Biol* **5**, 313-320 (1985).

40 Bhat, G. G. *et al.* Purification, characterization and molecular cloning of a monocot mannose-binding lectin from *Remusatia vivipara* with nematicidal activity. *Glycoconj J* **27**, 309-320 (2010).

41 Xia, Y. *et al.* The purL gene of *Bacillus subtilis* is associated with nematicidal activity. *FEMS Microbiol Lett* **322**, 99-107 (2011).

42 Atkinson, H. J., Grimwood, S., Johnston, K. & Green, J. Prototype demonstration of transgenic resistance to the nematode *Radopholus similis* conferred on banana by a cystatin. *Transgenic Res* **13**, 135-142 (2004).

43 Urwin, P. E., Atkinson, H. J., Waller, D. A. & McPherson, M. J. Engineered oryzacystatin-I expressed in transgenic hairy roots confers resistance to *Globodera pallida*. *Plant J* **8**, 121-131 (1995).

44 Fan, W., Idnurm, A., Breger, J., Mylonakis, E. & Heitman, J. Eca1, a sarcoplasmic/endoplasmic reticulum Ca^2+^-ATPase, is involved in stress tolerance and virulence in *Cryptococcus neoformans*. *Infect Immun* **75**, 3394-3405 (2007).

45 Mylonakis, E. *et al.* *Cryptococcus neoformans* Kin1 protein kinase homologue, identified through a *Caenorhabditis elegans* screen, promotes virulence in mammals. *Mol Microbiol* **54**, 407-419 (2004).

46 Jablonska, B. *et al.* The *Mi-9* gene from *Solanum arcanum* conferring heat-stable resistance to root-knot nematodes is a homolog of *Mi-1*. *Plant Physiol* **143**, 1044-1054 (2006).

47 Chou, T. C. *et al.* Enterohaemorrhagic *Escherichia coli* O157:H7 Shiga-like toxin 1 is required for full pathogenicity and activation of the p38 mitogen-activated protein kinase pathway in *Caenorhabditis elegans*. *Cell Microbiol* **15**, 82-97 (2013).

48 Ireland, D. C., Clark, R. J., Daly, N. L. & Craik, D. J. Isolation, sequencing, and structure-activity relationships of cyclotides. *J Nat Prod* **73**, 1610-1622 (2010).

49 Colgrave, M. L., Kotze, A. C., Ireland, D. C., Wang, C. K. & Craik, D. J. The anthelmintic activity of the cyclotides: natural variants with enhanced activity. *Chembiochem* **9**, 1939-1945 (2008).

50 Colgrave, M. L. *et al.* Anthelmintic activity of cyclotides: in vitro studies with canine and human hookworms. *Acta Trop* **109**, 163-166 (2009).

51 Huang, Y. H., Colgrave, M. L., Clark, R. J., Kotze, A. C. & Craik, D. J. Lysine-scanning mutagenesis reveals an amendable face of the cyclotide kalata B1 for the optimization of nematocidal activity. *J Biol Chem* **285**, 10797-10805 (2010).

52 Colgrave, M. L. *et al.* Cyclotides: natural, circular plant peptides that possess significant activity against gastrointestinal nematode parasites of Sheep. *Biochemistry* **47**, 5581-5589 (2008).

53 Liu, R. *et al.* Two antimicrobial and nematicidal peptides derived from sequences encoded *Picea sitchensis*. *J Pept Sci* **17**, 627-631 (2011).

54 Huang, L. *et al.* Enhanced production of 2-hydroxyphenazine in *Pseudomonas chlororaphis* GP72. *Appl Microbiol Biotechnol* **89**, 169-177 (2011).

55 Mahajan-Miklos, S., Tan, M.-W., Rahme, L. G. & Ausubel, F. M. Molecular mechanisms of bacterial virulence elucidated using a *Pseudomonas aeruginosa*–*Caenorhabditis elegans* pathogenesis model. *Cell* **96**, 47-56 (1999).

56 Noel, G. R. Evaluation of thuringiensin for control of *Heterodera glycines* on soybean. *J Nematol* **22**, 763-766 (1990).

57 Liu, X. Y. *et al.* Genome-wide screening reveals the genetic determinants of an antibiotic insecticide in *Bacillus thuringiensis*. *J Biol Chem* **285**, 39191-39200 (2010).

58 He, J. *et al.* Complete genome sequence of *Bacillus thuringiensis* subsp. *chinensis* strain CT-43. *J Bacteriol* **193**, 3407-3408 (2011).

59 Iatsenko, I., Nikolov, A. & Sommer, R. J. Identification of distinct *Bacillus thuringiensis* 4A4 nematicidal factors using the model nematodes *Pristionchus pacificus* and *Caenorhabditis elegans*. *Toxins* **6**, 2050-2063 (2014).

**Table S6** Putative virulence factors in the 120 nematicidal spore-forming Bacilli genomes

| strains | Bace16 | PurL | Eca1 | chitinase | BLG4 | Enp | Bmp1 | Bae16 | Thu | Nel | ColB | Cry5B | Cry5C | Cry5E | Cry12A | Cry13A | Cry14A | Cry21A | Cry21B | Cry6A | Cry6B | Cry55A |
| --- | --- | --- | --- | --- | --- | --- | --- | --- | --- | --- | --- | --- | --- | --- | --- | --- | --- | --- | --- | --- | --- | --- |
| Bt_G25-1 | 0 | 1 | 2 | 1 | 3 | 1 | 4 | 1 | 0 | 0 | 1 | 0 | 0 | 0 | 0 | 0 | 0 | 0 | 0 | 0 | 0 | 0 |
| Bt_G25-2 | 0 | 1 | 2 | 1 | 4 | 1 | 4 | 1 | 1 | 0 | 1 | 0 | 0 | 0 | 0 | 0 | 0 | 0 | 0 | 0 | 0 | 0 |
| Bt_G25-3 | 0 | 1 | 2 | 1 | 2 | 1 | 2 | 1 | 0 | 0 | 1 | 0 | 0 | 0 | 0 | 0 | 0 | 0 | 0 | 0 | 0 | 0 |
| Bt_G25-4 | 0 | 1 | 2 | 1 | 3 | 1 | 3 | 1 | 0 | 1 | 1 | 0 | 0 | 0 | 0 | 0 | 0 | 0 | 0 | 0 | 0 | 0 |
| Bt_G25-5 | 0 | 1 | 2 | 1 | 2 | 1 | 2 | 1 | 0 | 1 | 1 | 0 | 0 | 0 | 0 | 0 | 0 | 0 | 0 | 0 | 0 | 0 |
| Bt_G25-6 | 0 | 1 | 2 | 1 | 3 | 1 | 2 | 1 | 0 | 0 | 1 | 0 | 0 | 0 | 0 | 0 | 0 | 0 | 0 | 0 | 0 | 0 |
| Bt_G25-7 | 0 | 1 | 2 | 1 | 4 | 2 | 2 | 1 | 0 | 0 | 1 | 0 | 0 | 0 | 0 | 0 | 0 | 1 | 2 | 0 | 0 | 0 |
| Bt_G25-8 | 0 | 1 | 2 | 1 | 3 | 1 | 4 | 1 | 0 | 1 | 1 | 0 | 0 | 0 | 0 | 0 | 0 | 0 | 0 | 0 | 0 | 0 |
| Bt_G25-9 | 0 | 1 | 2 | 1 | 5 | 1 | 4 | 1 | 0 | 0 | 1 | 1 | 0 | 0 | 0 | 0 | 0 | 3 | 1 | 0 | 0 | 0 |
| Bt_G25-11 | 0 | 1 | 2 | 1 | 3 | 1 | 4 | 1 | 1 | 0 | 1 | 0 | 0 | 0 | 0 | 0 | 0 | 0 | 0 | 0 | 0 | 0 |
| Bt_G25-13 | 0 | 1 | 2 | 0 | 2 | 1 | 1 | 1 | 0 | 0 | 1 | 0 | 0 | 0 | 0 | 0 | 0 | 0 | 0 | 0 | 0 | 0 |
| Bt_G25-17 | 0 | 1 | 2 | 1 | 5 | 1 | 4 | 1 | 0 | 0 | 1 | 0 | 0 | 0 | 0 | 0 | 0 | 0 | 0 | 2 | 0 | 1 |
| Bt_G25-18 | 0 | 1 | 1 | 1 | 2 | 1 | 0 | 1 | 1 | 0 | 1 | 0 | 0 | 0 | 0 | 0 | 0 | 0 | 0 | 0 | 0 | 0 |
| Bt_G25-39 | 0 | 1 | 2 | 1 | 3 | 1 | 4 | 1 | 0 | 0 | 1 | 0 | 0 | 0 | 0 | 0 | 0 | 0 | 0 | 0 | 0 | 0 |
| Bt_G25-41 | 0 | 1 | 2 | 1 | 4 | 1 | 3 | 1 | 0 | 0 | 1 | 1 | 0 | 1 | 0 | 0 | 0 | 1 | 0 | 0 | 0 | 0 |
| Bt_G25-42 | 0 | 1 | 2 | 1 | 2 | 2 | 1 | 0 | 0 | 0 | 1 | 0 | 0 | 0 | 1 | 0 | 0 | 0 | 0 | 0 | 0 | 0 |
| Bt_G25-43 | 0 | 1 | 2 | 1 | 5 | 1 | 4 | 1 | 0 | 0 | 1 | 1 | 0 | 0 | 0 | 0 | 0 | 0 | 0 | 2 | 0 | 1 |
| Bt_G25-44 | 0 | 1 | 2 | 1 | 4 | 2 | 3 | 0 | 0 | 0 | 1 | 0 | 0 | 0 | 2 | 2 | 0 | 1 | 0 | 0 | 0 | 0 |
| Bt_G25-45 | 0 | 1 | 2 | 1 | 4 | 1 | 3 | 1 | 0 | 0 | 1 | 0 | 0 | 0 | 0 | 0 | 0 | 0 | 1 | 0 | 4 | 0 |
| Bt_G25-46 | 0 | 1 | 2 | 1 | 3 | 1 | 3 | 1 | 0 | 0 | 1 | 0 | 0 | 0 | 0 | 0 | 1 | 1 | 0 | 0 | 0 | 0 |
| Bt_G25-47 | 0 | 1 | 2 | 1 | 5 | 1 | 4 | 1 | 0 | 0 | 1 | 0 | 0 | 0 | 0 | 0 | 2 | 1 | 3 | 0 | 0 | 0 |
| Bt_G25-48 | 0 | 1 | 2 | 1 | 4 | 1 | 4 | 1 | 0 | 0 | 1 | 0 | 0 | 0 | 0 | 0 | 1 | 1 | 1 | 0 | 0 | 0 |
| Bt_G25-49 | 0 | 1 | 2 | 1 | 4 | 1 | 4 | 1 | 0 | 0 | 1 | 0 | 1 | 1 | 0 | 0 | 0 | 1 | 0 | 0 | 0 | 0 |
| Bt_G25-50 | 0 | 1 | 2 | 1 | 4 | 1 | 4 | 1 | 0 | 0 | 1 | 1 | 0 | 1 | 0 | 0 | 0 | 1 | 0 | 0 | 0 | 0 |
| Bt_G25-51 | 0 | 1 | 1 | 1 | 4 | 1 | 4 | 1 | 0 | 0 | 1 | 1 | 1 | 1 | 0 | 0 | 0 | 3 | 1 | 0 | 0 | 0 |
| Bt_G25-52 | 0 | 1 | 2 | 0 | 2 | 2 | 1 | 0 | 0 | 0 | 0 | 0 | 0 | 0 | 0 | 0 | 0 | 3 | 0 | 0 | 0 | 0 |
| Bt_G25-53 | 0 | 1 | 2 | 1 | 3 | 2 | 2 | 0 | 0 | 0 | 1 | 3 | 0 | 0 | 0 | 0 | 0 | 0 | 1 | 2 | 0 | 1 |
| Bt_G25-76 | 0 | 1 | 2 | 1 | 4 | 1 | 4 | 1 | 0 | 0 | 1 | 0 | 0 | 0 | 0 | 0 | 0 | 0 | 0 | 0 | 0 | 0 |
| Bt_G25-78 | 0 | 1 | 2 | 1 | 5 | 1 | 4 | 1 | 0 | 0 | 1 | 0 | 0 | 0 | 0 | 0 | 0 | 0 | 4 | 0 | 0 | 0 |
| Bt_G25-80 | 0 | 1 | 2 | 0 | 2 | 2 | 5 | 1 | 0 | 1 | 1 | 0 | 0 | 0 | 0 | 0 | 0 | 0 | 0 | 1 | 0 | 0 |
| Bt_G25-81 | 0 | 1 | 2 | 0 | 2 | 2 | 4 | 1 | 0 | 1 | 1 | 0 | 0 | 0 | 0 | 0 | 0 | 0 | 0 | 0 | 0 | 0 |
| Bt_G25-85 | 0 | 1 | 2 | 1 | 4 | 1 | 4 | 1 | 1 | 0 | 1 | 0 | 0 | 0 | 0 | 0 | 0 | 0 | 0 | 0 | 0 | 0 |
| Bt_G25-87 | 0 | 1 | 2 | 1 | 5 | 1 | 4 | 1 | 0 | 0 | 1 | 0 | 0 | 0 | 0 | 0 | 0 | 0 | 4 | 0 | 0 | 0 |
| Bt_G25-89 | 0 | 1 | 2 | 0 | 2 | 2 | 5 | 1 | 0 | 1 | 1 | 0 | 0 | 0 | 0 | 0 | 0 | 0 | 0 | 1 | 0 | 0 |
| Bt_G25-91 | 0 | 1 | 2 | 1 | 5 | 1 | 4 | 1 | 0 | 0 | 1 | 0 | 1 | 0 | 0 | 0 | 0 | 3 | 1 | 0 | 0 | 0 |
| Bt_G25-92 | 0 | 1 | 2 | 0 | 2 | 2 | 5 | 1 | 0 | 1 | 1 | 0 | 0 | 0 | 0 | 0 | 0 | 0 | 0 | 0 | 0 | 0 |
| Bt_G25-93 | 0 | 1 | 2 | 1 | 3 | 1 | 2 | 1 | 0 | 0 | 1 | 0 | 0 | 0 | 0 | 0 | 0 | 0 | 0 | 0 | 0 | 0 |
| Bt_G25-94 | 0 | 1 | 2 | 0 | 3 | 2 | 4 | 1 | 0 | 0 | 1 | 0 | 0 | 0 | 0 | 0 | 0 | 0 | 0 | 0 | 0 | 0 |
| Bt_G25-95 | 0 | 1 | 2 | 0 | 3 | 2 | 4 | 1 | 0 | 0 | 1 | 0 | 0 | 0 | 0 | 0 | 0 | 0 | 0 | 0 | 0 | 0 |
| Bt_G25-96 | 0 | 1 | 2 | 1 | 2 | 1 | 4 | 1 | 0 | 0 | 1 | 0 | 0 | 0 | 0 | 0 | 0 | 0 | 0 | 0 | 0 | 0 |
| Bt_G25-97 | 0 | 1 | 2 | 1 | 3 | 1 | 2 | 1 | 0 | 0 | 1 | 0 | 0 | 0 | 0 | 0 | 0 | 0 | 0 | 0 | 0 | 0 |
| Bt_G25-98 | 0 | 1 | 2 | 0 | 3 | 2 | 4 | 1 | 0 | 0 | 1 | 0 | 0 | 0 | 0 | 0 | 0 | 0 | 0 | 0 | 0 | 0 |
| Bt_G25-120 | 0 | 1 | 2 | 1 | 2 | 1 | 4 | 1 | 0 | 0 | 1 | 0 | 0 | 0 | 0 | 0 | 0 | 0 | 0 | 0 | 0 | 0 |
| Bt_G25-121 | 0 | 1 | 2 | 1 | 2 | 1 | 4 | 1 | 0 | 0 | 1 | 0 | 0 | 0 | 0 | 0 | 0 | 0 | 0 | 0 | 0 | 0 |
| Bc_G25-65 | 0 | 1 | 2 | 1 | 2 | 1 | 3 | 1 | 0 | 0 | 1 | 0 | 0 | 0 | 0 | 0 | 0 | 0 | 0 | 0 | 0 | 0 |
| Bc_G25-66 | 0 | 1 | 2 | 1 | 2 | 1 | 3 | 1 | 0 | 0 | 1 | 0 | 0 | 0 | 0 | 0 | 0 | 0 | 0 | 0 | 0 | 0 |
| Bc_G25-77 | 0 | 1 | 2 | 0 | 2 | 2 | 4 | 1 | 0 | 1 | 1 | 0 | 0 | 0 | 0 | 0 | 0 | 0 | 0 | 1 | 0 | 0 |
| Bc_G25-86 | 0 | 1 | 2 | 1 | 4 | 1 | 2 | 1 | 0 | 0 | 1 | 0 | 0 | 0 | 0 | 0 | 0 | 0 | 0 | 0 | 0 | 0 |
| Bc_G25-106 | 0 | 1 | 2 | 1 | 3 | 1 | 3 | 1 | 0 | 0 | 1 | 0 | 0 | 0 | 0 | 0 | 0 | 0 | 0 | 0 | 0 | 0 |
| Bw_G25-84 | 0 | 1 | 2 | 1 | 1 | 1 | 1 | 1 | 0 | 0 | 1 | 0 | 0 | 0 | 0 | 0 | 0 | 0 | 0 | 0 | 0 | 0 |
| Bw_G25-103 | 0 | 1 | 2 | 1 | 2 | 1 | 4 | 1 | 0 | 0 | 1 | 0 | 0 | 0 | 0 | 0 | 0 | 0 | 0 | 0 | 0 | 0 |
| Bw_G25-104 | 0 | 1 | 2 | 1 | 1 | 1 | 2 | 1 | 0 | 1 | 1 | 0 | 0 | 0 | 0 | 0 | 0 | 0 | 0 | 0 | 0 | 0 |
| Bw_G25-105 | 0 | 1 | 2 | 1 | 1 | 1 | 2 | 1 | 0 | 0 | 1 | 0 | 0 | 0 | 0 | 0 | 0 | 0 | 0 | 0 | 0 | 0 |
| Bto_G25-82 | 0 | 1 | 2 | 0 | 2 | 2 | 5 | 1 | 0 | 1 | 1 | 0 | 0 | 0 | 0 | 0 | 0 | 0 | 0 | 1 | 0 | 0 |
| Bto_G25-88 | 0 | 1 | 2 | 1 | 3 | 1 | 3 | 1 | 0 | 0 | 1 | 0 | 0 | 0 | 0 | 0 | 0 | 0 | 0 | 0 | 0 | 0 |
| Bto_G25-123 | 0 | 1 | 2 | 1 | 2 | 1 | 4 | 1 | 0 | 0 | 1 | 0 | 0 | 0 | 0 | 0 | 0 | 0 | 0 | 0 | 0 | 0 |
| Bmy_G25-101 | 0 | 1 | 2 | 1 | 2 | 1 | 3 | 1 | 0 | 0 | 1 | 0 | 0 | 0 | 0 | 0 | 0 | 0 | 0 | 0 | 0 | 0 |
| Bmy_G25-102 | 0 | 1 | 2 | 1 | 2 | 1 | 3 | 1 | 0 | 0 | 1 | 0 | 0 | 0 | 0 | 0 | 0 | 0 | 0 | 0 | 0 | 0 |
| Bmy_G25-108 | 0 | 1 | 2 | 1 | 2 | 1 | 2 | 1 | 0 | 0 | 1 | 0 | 0 | 0 | 0 | 0 | 0 | 0 | 0 | 0 | 0 | 0 |
| Bam_G25-32 | 1 | 1 | 1 | 0 | 3 | 0 | 0 | 1 | 0 | 0 | 0 | 0 | 0 | 0 | 0 | 0 | 0 | 0 | 0 | 0 | 0 | 0 |
| Bam_G25-110 | 1 | 1 | 1 | 0 | 3 | 0 | 0 | 1 | 0 | 0 | 0 | 0 | 0 | 0 | 0 | 0 | 0 | 0 | 0 | 0 | 0 | 0 |
| Bs_G25-83 | 1 | 1 | 1 | 0 | 2 | 1 | 0 | 1 | 0 | 0 | 0 | 0 | 0 | 0 | 0 | 0 | 0 | 0 | 0 | 0 | 0 | 0 |
| Bs_G25-31 | 1 | 1 | 1 | 0 | 2 | 1 | 0 | 1 | 0 | 0 | 0 | 0 | 0 | 0 | 0 | 0 | 0 | 0 | 0 | 0 | 0 | 0 |
| Bs_G25-40 | 1 | 1 | 1 | 0 | 2 | 1 | 0 | 1 | 0 | 0 | 0 | 0 | 0 | 0 | 0 | 0 | 0 | 0 | 0 | 0 | 0 | 0 |
| Bs_G25-60 | 1 | 1 | 1 | 0 | 2 | 0 | 0 | 1 | 0 | 0 | 0 | 0 | 0 | 0 | 0 | 0 | 0 | 0 | 0 | 0 | 0 | 0 |
| Bs_G25-61 | 1 | 1 | 1 | 0 | 2 | 1 | 0 | 1 | 0 | 0 | 0 | 0 | 0 | 0 | 0 | 0 | 0 | 0 | 0 | 0 | 0 | 0 |
| Bs_G25-90 | 1 | 1 | 1 | 0 | 2 | 1 | 0 | 1 | 0 | 0 | 0 | 0 | 0 | 0 | 0 | 0 | 0 | 0 | 0 | 0 | 0 | 0 |
| Bs_G25-135 | 1 | 1 | 1 | 0 | 3 | 1 | 0 | 1 | 0 | 0 | 0 | 0 | 0 | 0 | 0 | 0 | 0 | 0 | 0 | 0 | 0 | 0 |
| Bli_G25-99 | 1 | 1 | 1 | 1 | 3 | 0 | 0 | 0 | 0 | 0 | 0 | 0 | 0 | 0 | 0 | 0 | 0 | 0 | 0 | 0 | 0 | 0 |
| Bli_G25-100 | 1 | 1 | 1 | 1 | 3 | 0 | 0 | 0 | 0 | 0 | 0 | 0 | 0 | 0 | 0 | 0 | 0 | 0 | 0 | 0 | 0 | 0 |
| Bli_G25-136 | 1 | 1 | 1 | 0 | 3 | 0 | 0 | 0 | 0 | 0 | 0 | 0 | 0 | 0 | 0 | 0 | 0 | 0 | 0 | 0 | 0 | 0 |
| Bat_G25-122 | 1 | 1 | 1 | 1 | 2 | 1 | 0 | 0 | 0 | 0 | 0 | 0 | 0 | 0 | 0 | 0 | 0 | 0 | 0 | 0 | 0 | 0 |
| Bv_G25-127 | 1 | 1 | 1 | 0 | 2 | 0 | 0 | 1 | 0 | 0 | 0 | 0 | 0 | 0 | 0 | 0 | 0 | 0 | 0 | 0 | 0 | 0 |
| Bp_G25-35 | 0 | 1 | 1 | 0 | 5 | 0 | 0 | 0 | 0 | 0 | 0 | 0 | 0 | 0 | 0 | 0 | 0 | 0 | 0 | 0 | 0 | 0 |
| Bp_G25-36 | 0 | 1 | 1 | 0 | 4 | 0 | 0 | 0 | 0 | 0 | 0 | 0 | 0 | 0 | 0 | 0 | 0 | 0 | 0 | 0 | 0 | 0 |
| Bp_G25-37 | 0 | 1 | 1 | 0 | 6 | 0 | 0 | 0 | 0 | 0 | 0 | 0 | 0 | 0 | 0 | 0 | 0 | 0 | 0 | 0 | 0 | 0 |
| Bp_G25-38 | 0 | 1 | 1 | 0 | 6 | 0 | 0 | 0 | 0 | 0 | 0 | 0 | 0 | 0 | 0 | 0 | 0 | 0 | 0 | 0 | 0 | 0 |
| Bp_G25-63 | 0 | 1 | 1 | 0 | 6 | 0 | 0 | 0 | 0 | 0 | 0 | 0 | 0 | 0 | 0 | 0 | 0 | 0 | 0 | 0 | 0 | 0 |
| Bp_G25-64 | 0 | 1 | 1 | 0 | 6 | 0 | 0 | 0 | 0 | 0 | 0 | 0 | 0 | 0 | 0 | 0 | 0 | 0 | 0 | 0 | 0 | 0 |
| Bf_G25-26 | 0 | 1 | 1 | 0 | 6 | 3 | 0 | 0 | 0 | 0 | 0 | 0 | 0 | 0 | 0 | 0 | 0 | 0 | 0 | 0 | 0 | 0 |
| Bf_G25-27 | 0 | 1 | 1 | 0 | 5 | 3 | 0 | 0 | 0 | 0 | 0 | 0 | 0 | 0 | 0 | 0 | 0 | 0 | 0 | 0 | 0 | 0 |
| Bf_G25-55 | 0 | 1 | 1 | 0 | 3 | 1 | 0 | 0 | 0 | 0 | 0 | 0 | 0 | 0 | 0 | 0 | 0 | 0 | 0 | 0 | 0 | 0 |
| Bf_G25-56 | 0 | 1 | 1 | 0 | 3 | 1 | 0 | 0 | 0 | 0 | 0 | 0 | 0 | 0 | 0 | 0 | 0 | 0 | 0 | 0 | 0 | 0 |
| Bf_G25-58 | 0 | 1 | 1 | 0 | 3 | 1 | 0 | 0 | 0 | 0 | 0 | 0 | 0 | 0 | 0 | 0 | 0 | 0 | 0 | 0 | 0 | 0 |
| Bf_G25-67 | 0 | 1 | 1 | 0 | 3 | 1 | 0 | 0 | 0 | 0 | 0 | 0 | 0 | 0 | 0 | 0 | 0 | 0 | 0 | 0 | 0 | 0 |
| Bf_G25-69 | 0 | 1 | 1 | 0 | 4 | 1 | 0 | 0 | 0 | 0 | 0 | 0 | 0 | 0 | 0 | 0 | 0 | 0 | 0 | 0 | 0 | 0 |
| Bf_G25-70 | 0 | 1 | 1 | 0 | 4 | 1 | 0 | 0 | 0 | 0 | 0 | 0 | 0 | 0 | 0 | 0 | 0 | 0 | 0 | 0 | 0 | 0 |
| Bf_G25-72 | 0 | 1 | 1 | 0 | 4 | 1 | 0 | 0 | 0 | 0 | 0 | 0 | 0 | 0 | 0 | 0 | 0 | 0 | 0 | 0 | 0 | 0 |
| Bf_G25-73 | 0 | 1 | 1 | 0 | 3 | 1 | 0 | 0 | 0 | 0 | 0 | 0 | 0 | 0 | 0 | 0 | 0 | 0 | 0 | 0 | 0 | 0 |
| Bm_G25-30 | 0 | 1 | 1 | 0 | 1 | 0 | 0 | 1 | 0 | 0 | 0 | 0 | 0 | 0 | 0 | 0 | 0 | 0 | 0 | 0 | 0 | 0 |
| Bar_G25-109 | 0 | 1 | 1 | 0 | 1 | 0 | 0 | 1 | 0 | 0 | 0 | 0 | 0 | 0 | 0 | 0 | 0 | 0 | 0 | 0 | 0 | 0 |
| Ble_G25-134 | 0 | 1 | 1 | 1 | 2 | 0 | 0 | 0 | 0 | 0 | 0 | 0 | 0 | 0 | 0 | 0 | 0 | 0 | 0 | 0 | 0 | 0 |
| Bst_G25-119 | 0 | 1 | 1 | 0 | 5 | 0 | 0 | 0 | 0 | 0 | 0 | 0 | 0 | 0 | 0 | 0 | 0 | 0 | 0 | 0 | 0 | 0 |
| Bst_G25-132-1 | 1 | 1 | 1 | 0 | 4 | 0 | 0 | 0 | 0 | 0 | 0 | 0 | 0 | 0 | 0 | 0 | 0 | 0 | 0 | 0 | 0 | 0 |
| Be_G25-135-1 | 1 | 1 | 1 | 0 | 1 | 0 | 0 | 0 | 0 | 0 | 0 | 0 | 0 | 0 | 0 | 0 | 0 | 0 | 0 | 0 | 0 | 0 |
| Bmu_G25-68 | 0 | 1 | 1 | 0 | 1 | 1 | 0 | 1 | 0 | 0 | 0 | 0 | 0 | 0 | 0 | 0 | 0 | 0 | 0 | 0 | 0 | 0 |
| Bg_G25-74 | 0 | 1 | 1 | 1 | 1 | 0 | 0 | 0 | 0 | 0 | 0 | 0 | 0 | 0 | 0 | 0 | 0 | 0 | 0 | 0 | 0 | 0 |
| Fbp_G25-29 | 0 | 1 | 1 | 0 | 3 | 4 | 0 | 0 | 0 | 0 | 0 | 0 | 0 | 0 | 0 | 0 | 0 | 0 | 0 | 0 | 0 | 0 |
| Fba_G25-54 | 0 | 1 | 1 | 0 | 6 | 2 | 0 | 0 | 0 | 0 | 0 | 0 | 0 | 0 | 0 | 0 | 0 | 0 | 0 | 0 | 0 | 0 |
| Lbs_G25-33 | 0 | 1 | 1 | 0 | 1 | 0 | 0 | 0 | 0 | 0 | 0 | 0 | 0 | 0 | 0 | 0 | 0 | 0 | 0 | 0 | 0 | 0 |
| Lbs_G25-34 | 0 | 1 | 1 | 0 | 1 | 0 | 0 | 0 | 0 | 0 | 0 | 0 | 0 | 0 | 0 | 0 | 0 | 0 | 0 | 0 | 0 | 0 |
| Lbs_G25-62 | 0 | 1 | 1 | 0 | 1 | 0 | 0 | 0 | 0 | 0 | 0 | 0 | 0 | 0 | 0 | 0 | 0 | 0 | 0 | 0 | 0 | 0 |
| Lbs_G25-111 | 0 | 1 | 1 | 0 | 1 | 0 | 0 | 0 | 0 | 0 | 0 | 0 | 0 | 0 | 0 | 0 | 0 | 0 | 0 | 0 | 0 | 0 |
| Lbs_G25-112 | 0 | 1 | 1 | 0 | 1 | 0 | 0 | 0 | 0 | 0 | 0 | 0 | 0 | 0 | 0 | 0 | 0 | 0 | 0 | 0 | 0 | 0 |
| Lbs_G25-114 | 0 | 1 | 1 | 0 | 1 | 0 | 0 | 0 | 0 | 0 | 0 | 0 | 0 | 0 | 0 | 0 | 0 | 0 | 0 | 0 | 0 | 0 |
| Lbs_G25-115 | 0 | 1 | 1 | 0 | 1 | 0 | 0 | 0 | 0 | 0 | 0 | 0 | 0 | 0 | 0 | 0 | 0 | 0 | 0 | 0 | 0 | 0 |
| Lbs_G25-116 | 0 | 1 | 1 | 0 | 1 | 0 | 0 | 0 | 0 | 0 | 0 | 0 | 0 | 0 | 0 | 0 | 0 | 0 | 0 | 0 | 0 | 0 |
| Lbf_G25-113 | 0 | 1 | 1 | 0 | 1 | 0 | 0 | 0 | 0 | 0 | 0 | 0 | 0 | 0 | 0 | 0 | 0 | 0 | 0 | 0 | 0 | 0 |
| Pbl_G25-75 | 0 | 1 | 1 | 0 | 2 | 0 | 0 | 3 | 0 | 0 | 0 | 0 | 0 | 0 | 0 | 0 | 0 | 0 | 0 | 0 | 0 | 0 |
| Pba_G25-117 | 0 | 1 | 1 | 2 | 4 | 0 | 0 | 0 | 0 | 0 | 0 | 0 | 0 | 0 | 0 | 0 | 0 | 0 | 0 | 0 | 0 | 0 |
| Pba_G25-118 | 0 | 1 | 1 | 2 | 4 | 0 | 0 | 0 | 0 | 0 | 0 | 0 | 0 | 0 | 0 | 0 | 0 | 0 | 0 | 0 | 0 | 0 |
| Pbd_G25-126 | 0 | 1 | 2 | 2 | 2 | 0 | 0 | 0 | 0 | 0 | 0 | 0 | 0 | 0 | 0 | 0 | 0 | 0 | 0 | 0 | 0 | 0 |
| Pbp_G25-124 | 0 | 1 | 1 | 0 | 2 | 1 | 0 | 0 | 0 | 0 | 0 | 0 | 0 | 0 | 0 | 0 | 0 | 0 | 0 | 0 | 0 | 0 |
| Bbl_G25-128 | 0 | 1 | 1 | 0 | 3 | 1 | 0 | 0 | 0 | 0 | 0 | 0 | 0 | 0 | 0 | 0 | 0 | 0 | 0 | 0 | 0 | 0 |
| Bbl_G25-129 | 0 | 1 | 1 | 0 | 3 | 1 | 0 | 0 | 0 | 0 | 0 | 0 | 0 | 0 | 0 | 0 | 0 | 0 | 0 | 0 | 0 | 0 |
| Bbl_G25-130 | 0 | 1 | 1 | 0 | 3 | 1 | 0 | 0 | 0 | 0 | 0 | 0 | 0 | 0 | 0 | 0 | 0 | 0 | 0 | 0 | 0 | 0 |
| Bbl_G25-131 | 0 | 1 | 1 | 0 | 3 | 1 | 0 | 0 | 0 | 0 | 0 | 0 | 0 | 0 | 0 | 0 | 0 | 0 | 0 | 0 | 0 | 0 |
| Bbb_G25-125 | 0 | 1 | 1 | 0 | 4 | 1 | 0 | 0 | 0 | 0 | 0 | 0 | 0 | 0 | 0 | 0 | 0 | 0 | 0 | 0 | 0 | 0 |
| Bbb_G25-137 | 0 | 1 | 1 | 0 | 3 | 1 | 0 | 0 | 0 | 0 | 0 | 0 | 0 | 0 | 0 | 0 | 0 | 0 | 0 | 0 | 0 | 0 |
| Bba_G25-57 | 0 | 1 | 1 | 0 | 3 | 0 | 0 | 1 | 0 | 0 | 0 | 0 | 0 | 0 | 0 | 0 | 0 | 0 | 0 | 0 | 0 | 0 |

Note: Strain names were abbreviated as follows: Bt for *B. thuringiensis*, Bc for *B. cereus*, Bw for *B. weihenstephanensis*, Bto for *B. toyonensis*, Bmy for *B. mycoides*, Bam for *B. amyloliquefaciens*, Bs for *B. subtilis*, Bli for *B. licheniformis*, Bat for *B. atrophaeus*, Bv for *B. vallismortis*, Bp for *B. pumilus*, Bf for *B. firmus*, Bm for *B. megaterium*, Bar for *B. aryabhattai*, Bl for *B. lehensis*, Bst for *B. stratosphericus*, Be for *B. endophyticus*, Bmu for *B. muralis*, Bg for *B. galactosidilyticus*, Fbp for *F. phosphorivorans*, Fba for *F. arsenicus*, Lbs for *L. sphaericus*, Lbf for *L. fusiformis*, Pbl for *P. larvae*, Pba for *P. alvei*, Pbd for *P. dendritiformis*, Pbp for *P. polymyxa*, Bbl for *B. laterosporus*, Bbb for *B. brevis* and Bba for *B. agri*.

**Table S7** Number of different putative virulence factors among different spore-forming Bacilli species

| nematicidal factors | Number of putative virulence factors per species | | | | | | | | | | | | | | | | | | | | | | | | | | | | | |
| --- | --- | --- | --- | --- | --- | --- | --- | --- | --- | --- | --- | --- | --- | --- | --- | --- | --- | --- | --- | --- | --- | --- | --- | --- | --- | --- | --- | --- | --- | --- |
|  | *B. cereus* group | | | | | *B. subtilis* group | | | | |  |  |  |  |  |  |  |  |  |  |  |  |  |  |  |  |  |  |  |  |
|  | *Bt* | *Bc* | *Bw* | *Bto* | *Bmy* | *Bam* | *Bs* | *Bli* | *Bat* | *Bv* | *Bp* | *Bf* | *Bm* | *Bar* | *Ble* | *Bst* | *Be* | *Bmu* | *Bg* | *Fp* | *Fa* | *Lbs* | *Lbf* | *Pbl* | *Pba* | *Pbd* | *Pbp* | *Bbl* | *Bbb* | *Bba* |
| **All putative virulence factors** | | | | | | | | | | | | | | | | | | | | | | | | | | | | | | |
|  | 708 | 65 | 43 | 42 | 35 | 14 | 49 | 20 | 7 | 6 | 45 | 72 | 4 | 4 | 5 | 14 | 4 | 5 | 4 | 9 | 10 | 24 | 3 | 7 | 16 | 7 | 5 | 24 | 13 | 6 |
| **Proteases** | | | | | | | | | | | | | | | | | | | | | | | | | | | | | | |
| Bace16 | 0 | 0 | 0 | 0 | 0 | 2 | 7 | 3 | 1 | 1 | 0 | 0 | 0 | 0 | 0 | 1 | 1 | 0 | 0 | 0 | 0 | 0 | 0 | 0 | 0 | 0 | 0 | 0 | 0 | 0 |
| Bae16 | 40 | 5 | 4 | 3 | 3 | 2 | 7 | 0 | 0 | 1 | 0 | 0 | 1 | 1 | 0 | 0 | 0 | 1 | 0 | 0 | 0 | 0 | 0 | 3 | 0 | 0 | 0 | 0 | 0 | 1 |
| Bmp1 | 147 | 15 | 9 | 12 | 8 | 0 | 0 | 0 | 0 | 0 | 0 | 0 | 0 | 0 | 0 | 0 | 0 | 0 | 0 | 0 | 0 | 0 | 0 | 0 | 0 | 0 | 0 | 0 | 0 | 0 |
| ColB | 43 | 5 | 4 | 3 | 3 | 0 | 0 | 0 | 0 | 0 | 0 | 0 | 0 | 0 | 0 | 0 | 0 | 0 | 0 | 0 | 0 | 0 | 0 | 0 | 0 | 0 | 0 | 0 | 0 | 0 |
| Enp | 56 | 6 | 4 | 4 | 3 | 0 | 6 | 0 | 1 | 0 | 0 | 14 | 0 | 0 | 0 | 0 | 0 | 1 | 0 | 4 | 2 | 0 | 0 | 0 | 0 | 0 | 1 | 4 | 2 | 0 |
| BLG4 | 144 | 13 | 5 | 7 | 6 | 6 | 15 | 9 | 2 | 2 | 33 | 38 | 1 | 1 | 2 | 9 | 1 | 1 | 1 | 3 | 6 | 8 | 1 | 2 | 8 | 2 | 2 | 12 | 7 | 3 |
| **Chitinases** | | | | | | | | | | | | | | | | | | | | | | | | | | | | | | |
| Chitinase | 35 | 4 | 4 | 2 | 3 | 0 | 0 | 2 | 1 | 0 | 0 | 0 | 0 | 0 | 1 | 0 | 0 | 0 | 1 | 0 | 0 | 0 | 0 | 0 | 4 | 2 | 0 | 0 | 0 | 0 |
| **Cry proteins** | | | | | | | | | | | | | | | | | | | | | | | | | | | | | | |
| Cry5B | 8 | 0 | 0 | 0 | 0 | 0 | 0 | 0 | 0 | 0 | 0 | 0 | 0 | 0 | 0 | 0 | 0 | 0 | 0 | 0 | 0 | 0 | 0 | 0 | 0 | 0 | 0 | 0 | 0 | 0 |
| Cry5C | 3 | 0 | 0 | 0 | 0 | 0 | 0 | 0 | 0 | 0 | 0 | 0 | 0 | 0 | 0 | 0 | 0 | 0 | 0 | 0 | 0 | 0 | 0 | 0 | 0 | 0 | 0 | 0 | 0 | 0 |
| Cry5E | 4 | 0 | 0 | 0 | 0 | 0 | 0 | 0 | 0 | 0 | 0 | 0 | 0 | 0 | 0 | 0 | 0 | 0 | 0 | 0 | 0 | 0 | 0 | 0 | 0 | 0 | 0 | 0 | 0 | 0 |
| Cry6A | 8 | 1 | 0 | 1 | 0 | 0 | 0 | 0 | 0 | 0 | 0 | 0 | 0 | 0 | 0 | 0 | 0 | 0 | 0 | 0 | 0 | 0 | 0 | 0 | 0 | 0 | 0 | 0 | 0 | 0 |
| Cry6B | 4 | 0 | 0 | 0 | 0 | 0 | 0 | 0 | 0 | 0 | 0 | 0 | 0 | 0 | 0 | 0 | 0 | 0 | 0 | 0 | 0 | 0 | 0 | 0 | 0 | 0 | 0 | 0 | 0 | 0 |
| Cry12A | 3 | 0 | 0 | 0 | 0 | 0 | 0 | 0 | 0 | 0 | 0 | 0 | 0 | 0 | 0 | 0 | 0 | 0 | 0 | 0 | 0 | 0 | 0 | 0 | 0 | 0 | 0 | 0 | 0 | 0 |
| Cry13A | 2 | 0 | 0 | 0 | 0 | 0 | 0 | 0 | 0 | 0 | 0 | 0 | 0 | 0 | 0 | 0 | 0 | 0 | 0 | 0 | 0 | 0 | 0 | 0 | 0 | 0 | 0 | 0 | 0 | 0 |
| Cry14A | 4 | 0 | 0 | 0 | 0 | 0 | 0 | 0 | 0 | 0 | 0 | 0 | 0 | 0 | 0 | 0 | 0 | 0 | 0 | 0 | 0 | 0 | 0 | 0 | 0 | 0 | 0 | 0 | 0 | 0 |
| Cry21A | 20 | 0 | 0 | 0 | 0 | 0 | 0 | 0 | 0 | 0 | 0 | 0 | 0 | 0 | 0 | 0 | 0 | 0 | 0 | 0 | 0 | 0 | 0 | 0 | 0 | 0 | 0 | 0 | 0 | 0 |
| Cry21B | 19 | 0 | 0 | 0 | 0 | 0 | 0 | 0 | 0 | 0 | 0 | 0 | 0 | 0 | 0 | 0 | 0 | 0 | 0 | 0 | 0 | 0 | 0 | 0 | 0 | 0 | 0 | 0 | 0 | 0 |
| Cry55A | 3 | 0 | 0 | 0 | 0 | 0 | 0 | 0 | 0 | 0 | 0 | 0 | 0 | 0 | 0 | 0 | 0 | 0 | 0 | 0 | 0 | 0 | 0 | 0 | 0 | 0 | 0 | 0 | 0 | 0 |
| **Other proteins** | | | | | | | | | | | | | | | | | | | | | | | | | | | | | | |
| PurL | 44 | 5 | 4 | 3 | 3 | 2 | 7 | 3 | 1 | 1 | 6 | 10 | 1 | 1 | 1 | 2 | 1 | 1 | 1 | 1 | 1 | 8 | 1 | 1 | 2 | 1 | 1 | 4 | 2 | 1 |
| Eca1 | 86 | 10 | 8 | 6 | 6 | 2 | 7 | 3 | 1 | 1 | 6 | 10 | 1 | 1 | 1 | 2 | 1 | 1 | 1 | 1 | 1 | 8 | 1 | 1 | 2 | 2 | 1 | 4 | 2 | 1 |
| Nel | 7 | 1 | 1 | 1 | 0 | 0 | 0 | 0 | 0 | 0 | 0 | 0 | 0 | 0 | 0 | 0 | 0 | 0 | 0 | 0 | 0 | 0 | 0 | 0 | 0 | 0 | 0 | 0 | 0 | 0 |
| **Compounds** | | | | | | | | | | | | | | | | | | | | | | | | | | | | | | |
| thuringiensin | 4 | 0 | 0 | 0 | 0 | 0 | 0 | 0 | 0 | 0 | 0 | 0 | 0 | 0 | 0 | 0 | 0 | 0 | 0 | 0 | 0 | 0 | 0 | 0 | 0 | 0 | 0 | 0 | 0 | 0 |

Note: Strain names were abbreviated as follows: Bt for *B. thuringiensis*, Bc for *B. cereus*, Bw for *B. weihenstephanensis*, Bto for *B. toyonensis*, Bmy for *B. mycoides*, Bam for *B. amyloliquefaciens*, Bs for *B. subtilis*, Bli for *B. licheniformis*, Bat for *B. atrophaeus*, Bv for *B. vallismortis*, Bp for *B. pumilus*, Bf for *B. firmus*, Bm for *B. megaterium*, Bar for *B. aryabhattai*, Bl for *B. lehensis*, Bst for *B. stratosphericus*, Be for *B. endophyticus*, Bmu for *B. muralis*, Bg for *B. galactosidilyticus*, Fbp for *F. phosphorivorans*, Fba for *F. arsenicus*, Lbs for *L. sphaericus*, Lbf for *L. fusiformis*, Pbl for *P. larvae*, Pba for *P. alvei*, Pbd for *P. dendritiformis*, Pbp for *P. polymyxa*, Bbl for *B. laterosporus*, Bbb for *B. brevis* and Bba for *B. agri*.

**Table S8** Relationship between mechanisms of putative virulence factors and capacities of nematicidal spore-forming Bacilli

| **Pore-forming mechanism of Cry** | | | | | | | | | | | | | | | | | | | | | | | |
| --- | --- | --- | --- | --- | --- | --- | --- | --- | --- | --- | --- | --- | --- | --- | --- | --- | --- | --- | --- | --- | --- | --- | --- |
| strains | c/10 | Bace16 | PurL | Eca1 | chitinase | BLG4 | Enp | Bmp1 | Bae16 | Thu | Nel | ColB | Cry5B | Cry5C | Cry5E | Cry12A | Cry13A | Cry14A | Cry21A | Cry21B | Cry6A | Cry6B | Cry55A |
| Bt_G25-7 | 0.524 | 0 | 1 | 2 | 1 | 4 | 2 | 2 | 1 | 0 | 0 | 1 | 0 | 0 | 0 | 0 | 0 | 0 | 1 | 2 | 0 | 0 | 0 |
| Bt_G25-9 | 0.847 | 0 | 1 | 2 | 1 | 5 | 1 | 4 | 1 | 0 | 0 | 1 | 1 | 0 | 0 | 0 | 0 | 0 | 3 | 1 | 0 | 0 | 0 |
| Bt_G25-17 | 0.841 | 0 | 1 | 2 | 1 | 5 | 1 | 4 | 1 | 0 | 0 | 1 | 0 | 0 | 0 | 0 | 0 | 0 | 0 | 0 | 2 | 0 | 1 |
| Bt_G25-41 | 0.85 | 0 | 1 | 2 | 1 | 4 | 1 | 3 | 1 | 0 | 0 | 1 | 1 | 0 | 1 | 0 | 0 | 0 | 1 | 0 | 0 | 0 | 0 |
| Bt_G25-43 | 0.839 | 0 | 1 | 2 | 1 | 5 | 1 | 4 | 1 | 0 | 0 | 1 | 1 | 0 | 0 | 0 | 0 | 0 | 0 | 0 | 2 | 0 | 1 |
| Bt_G25-45 | 0.848 | 0 | 1 | 2 | 1 | 4 | 1 | 3 | 1 | 0 | 0 | 1 | 0 | 0 | 0 | 0 | 0 | 0 | 0 | 1 | 0 | 4 | 0 |
| Bt_G25-46 | 0.85 | 0 | 1 | 2 | 1 | 3 | 1 | 3 | 1 | 0 | 0 | 1 | 0 | 0 | 0 | 0 | 0 | 1 | 1 | 0 | 0 | 0 | 0 |
| Bt_G25-47 | 0.747 | 0 | 1 | 2 | 1 | 5 | 1 | 4 | 1 | 0 | 0 | 1 | 0 | 0 | 0 | 0 | 0 | 2 | 1 | 3 | 0 | 0 | 0 |
| Bt_G25-48 | 0.693 | 0 | 1 | 2 | 1 | 4 | 1 | 4 | 1 | 0 | 0 | 1 | 0 | 0 | 0 | 0 | 0 | 1 | 1 | 1 | 0 | 0 | 0 |
| Bt_G25-49 | 0.778 | 0 | 1 | 2 | 1 | 4 | 1 | 4 | 1 | 0 | 0 | 1 | 0 | 1 | 1 | 0 | 0 | 0 | 1 | 0 | 0 | 0 | 0 |
| Bt_G25-50 | 0.662 | 0 | 1 | 2 | 1 | 4 | 1 | 4 | 1 | 0 | 0 | 1 | 1 | 0 | 1 | 0 | 0 | 0 | 1 | 0 | 0 | 0 | 0 |
| Bt_G25-51 | 0.85 | 0 | 1 | 1 | 1 | 4 | 1 | 4 | 1 | 0 | 0 | 1 | 1 | 1 | 1 | 0 | 0 | 0 | 3 | 1 | 0 | 0 | 0 |
| Bt_G25-78 | 0.73 | 0 | 1 | 2 | 1 | 5 | 1 | 4 | 1 | 0 | 0 | 1 | 0 | 0 | 0 | 0 | 0 | 0 | 0 | 4 | 0 | 0 | 0 |
| Bt_G25-87 | 0.833 | 0 | 1 | 2 | 1 | 5 | 1 | 4 | 1 | 0 | 0 | 1 | 0 | 0 | 0 | 0 | 0 | 0 | 0 | 4 | 0 | 0 | 0 |
| Bt_G25-91 | 0.797 | 0 | 1 | 2 | 1 | 5 | 1 | 4 | 1 | 0 | 0 | 1 | 0 | 1 | 0 | 0 | 0 | 0 | 3 | 1 | 0 | 0 | 0 |
| Bt_G25-1 | 0.56 | 0 | 1 | 2 | 1 | 3 | 1 | 4 | 1 | 0 | 0 | 1 | 0 | 0 | 0 | 0 | 0 | 0 | 0 | 0 | 0 | 0 | 0 |
| Bt_G25-3 | 0.489 | 0 | 1 | 2 | 1 | 2 | 1 | 2 | 1 | 0 | 0 | 1 | 0 | 0 | 0 | 0 | 0 | 0 | 0 | 0 | 0 | 0 | 0 |
| Bt_G25-6 | 0.536 | 0 | 1 | 2 | 1 | 3 | 1 | 2 | 1 | 0 | 0 | 1 | 0 | 0 | 0 | 0 | 0 | 0 | 0 | 0 | 0 | 0 | 0 |
| Bt_G25-39 | 0.664 | 0 | 1 | 2 | 1 | 3 | 1 | 4 | 1 | 0 | 0 | 1 | 0 | 0 | 0 | 0 | 0 | 0 | 0 | 0 | 0 | 0 | 0 |
| Bt_G25-96 | 0.242 | 0 | 1 | 2 | 1 | 2 | 1 | 4 | 1 | 0 | 0 | 1 | 0 | 0 | 0 | 0 | 0 | 0 | 0 | 0 | 0 | 0 | 0 |
| Bt_G25-120 | 0.397 | 0 | 1 | 2 | 1 | 2 | 1 | 4 | 1 | 0 | 0 | 1 | 0 | 0 | 0 | 0 | 0 | 0 | 0 | 0 | 0 | 0 | 0 |
| Bt_G25-121 | 0.172 | 0 | 1 | 2 | 1 | 2 | 1 | 4 | 1 | 0 | 0 | 1 | 0 | 0 | 0 | 0 | 0 | 0 | 0 | 0 | 0 | 0 | 0 |
| Note: Nematicidal Cry represents a whole pore-forming mechanism; factors except Cry are present or absent at the same column with a difference of number. This mechanism is shown as Fig. 3A. | | | | | | | | | | | | | | | | | | | | | | | |
| **Inhibition-like mechanism of thuringiensin** | | | | | | | | | | | | | | | | | | | | | | | |
| strains | c/10 | Bace16 | PurL | Eca1 | chitinase | BLG4 | Enp | Bmp1 | Bae16 | Thu | Nel | ColB | Cry5B | Cry5C | Cry5E | Cry12A | Cry13A | Cry14A | Cry21A | Cry21B | Cry6A | Cry6B | Cry55A |
| Bt_G25-2 | 0.55 | 0 | 1 | 2 | 1 | 4 | 1 | 4 | 1 | 1 | 0 | 1 | 0 | 0 | 0 | 0 | 0 | 0 | 0 | 0 | 0 | 0 | 0 |
| Bt_G25-11 | 0.842 | 0 | 1 | 2 | 1 | 3 | 1 | 4 | 1 | 1 | 0 | 1 | 0 | 0 | 0 | 0 | 0 | 0 | 0 | 0 | 0 | 0 | 0 |
| Bt_G25-18 | 0.836 | 0 | 1 | 1 | 1 | 2 | 1 | 0 | 1 | 1 | 0 | 1 | 0 | 0 | 0 | 0 | 0 | 0 | 0 | 0 | 0 | 0 | 0 |
| Bt_G25-85 | 0.767 | 0 | 1 | 2 | 1 | 4 | 1 | 4 | 1 | 1 | 0 | 1 | 0 | 0 | 0 | 0 | 0 | 0 | 0 | 0 | 0 | 0 | 0 |
| Bt_G25-1 | 0.56 | 0 | 1 | 2 | 1 | 3 | 1 | 4 | 1 | 0 | 0 | 1 | 0 | 0 | 0 | 0 | 0 | 0 | 0 | 0 | 0 | 0 | 0 |
| Bt_G25-3 | 0.489 | 0 | 1 | 2 | 1 | 2 | 1 | 2 | 1 | 0 | 0 | 1 | 0 | 0 | 0 | 0 | 0 | 0 | 0 | 0 | 0 | 0 | 0 |
| Bt_G25-6 | 0.536 | 0 | 1 | 2 | 1 | 3 | 1 | 2 | 1 | 0 | 0 | 1 | 0 | 0 | 0 | 0 | 0 | 0 | 0 | 0 | 0 | 0 | 0 |
| Bt_G25-96 | 0.242 | 0 | 1 | 2 | 1 | 2 | 1 | 4 | 1 | 0 | 0 | 1 | 0 | 0 | 0 | 0 | 0 | 0 | 0 | 0 | 0 | 0 | 0 |
| Bt_G25-120 | 0.397 | 0 | 1 | 2 | 1 | 2 | 1 | 4 | 1 | 0 | 0 | 1 | 0 | 0 | 0 | 0 | 0 | 0 | 0 | 0 | 0 | 0 | 0 |
| Bt_G25-121 | 0.172 | 0 | 1 | 2 | 1 | 2 | 1 | 4 | 1 | 0 | 0 | 1 | 0 | 0 | 0 | 0 | 0 | 0 | 0 | 0 | 0 | 0 | 0 |
| Note: Nematicidal thuringiensin kill nematodes with similar blocking mechanism in insects; factors except thuringiensin are present or absent at the same column with a difference of number. This putative mechanism is shown as Fig. 3B. | | | | | | | | | | | | | | | | | | | | | | | |
| **Putative degradation mechanisms** | | | | | | | | | | | | | | | | | | | | | | | |
| Type 1 of putative degradation mechanisms (intestine) | | | | | | | | | | | | | | | | | | | | | | | |
| strains | c/10 | Bace16 | PurL | Eca1 | chitinase | BLG4 | Enp | Bmp1 | Bae16 | Thu | Nel | ColB | Cry5B | Cry5C | Cry5E | Cry12A | Cry13A | Cry14A | Cry21A | Cry21B | Cry6A | Cry6B | Cry55A |
| Bam_G25-32 | 0.618 | 1 | 1 | 1 | 0 | 3 | 0 | 0 | 1 | 0 | 0 | 0 | 0 | 0 | 0 | 0 | 0 | 0 | 0 | 0 | 0 | 0 | 0 |
| Bam_G25-110 | 0.367 | 1 | 1 | 1 | 0 | 3 | 0 | 0 | 1 | 0 | 0 | 0 | 0 | 0 | 0 | 0 | 0 | 0 | 0 | 0 | 0 | 0 | 0 |
| Bs_G25-60 | 0.357 | 1 | 1 | 1 | 0 | 2 | 0 | 0 | 1 | 0 | 0 | 0 | 0 | 0 | 0 | 0 | 0 | 0 | 0 | 0 | 0 | 0 | 0 |
| Bv_G25-127 | 0.443 | 1 | 1 | 1 | 0 | 2 | 0 | 0 | 1 | 0 | 0 | 0 | 0 | 0 | 0 | 0 | 0 | 0 | 0 | 0 | 0 | 0 | 0 |
| Bp_G25-37 | 0.465 | 0 | 1 | 1 | 0 | 6 | 0 | 0 | 0 | 0 | 0 | 0 | 0 | 0 | 0 | 0 | 0 | 0 | 0 | 0 | 0 | 0 | 0 |
| Bp_G25-38 | 0.432 | 0 | 1 | 1 | 0 | 6 | 0 | 0 | 0 | 0 | 0 | 0 | 0 | 0 | 0 | 0 | 0 | 0 | 0 | 0 | 0 | 0 | 0 |
| Bp_G25-63 | 0.275 | 0 | 1 | 1 | 0 | 6 | 0 | 0 | 0 | 0 | 0 | 0 | 0 | 0 | 0 | 0 | 0 | 0 | 0 | 0 | 0 | 0 | 0 |
| Bp_G25-64 | 0.188 | 0 | 1 | 1 | 0 | 6 | 0 | 0 | 0 | 0 | 0 | 0 | 0 | 0 | 0 | 0 | 0 | 0 | 0 | 0 | 0 | 0 | 0 |
| Bst_G25-119 | 0.183 | 0 | 1 | 1 | 0 | 5 | 0 | 0 | 0 | 0 | 0 | 0 | 0 | 0 | 0 | 0 | 0 | 0 | 0 | 0 | 0 | 0 | 0 |
| Lbs_G25-111 | 0.18 | 0 | 1 | 1 | 0 | 1 | 0 | 0 | 0 | 0 | 0 | 0 | 0 | 0 | 0 | 0 | 0 | 0 | 0 | 0 | 0 | 0 | 0 |
| Lbs_G25-112 | 0.246 | 0 | 1 | 1 | 0 | 1 | 0 | 0 | 0 | 0 | 0 | 0 | 0 | 0 | 0 | 0 | 0 | 0 | 0 | 0 | 0 | 0 | 0 |
| Lbs_G25-114 | 0.374 | 0 | 1 | 1 | 0 | 1 | 0 | 0 | 0 | 0 | 0 | 0 | 0 | 0 | 0 | 0 | 0 | 0 | 0 | 0 | 0 | 0 | 0 |
| Note: Trojan horse mechanism of putative Bace16 and Bae16 (subtype 1); factors except putative Bace16 and Bae16 are present or absent at the same column with a difference of number; putative Enp is absent. This putative mechanism is shown as Fig. 3E (left). | | | | | | | | | | | | | | | | | | | | | | | |
| strains | c/10 | Bace16 | PurL | Eca1 | chitinase | BLG4 | Enp | Bmp1 | Bae16 | Thu | Nel | ColB | Cry5B | Cry5C | Cry5E | Cry12A | Cry13A | Cry14A | Cry21A | Cry21B | Cry6A | Cry6B | Cry55A |
| Bt_G25-13 | 0.833 | 0 | 1 | 2 | 0 | 2 | 1 | 1 | 1 | 0 | 0 | 1 | 0 | 0 | 0 | 0 | 0 | 0 | 0 | 0 | 0 | 0 | 0 |
| Bt_G25-94 | 0.558 | 0 | 1 | 2 | 0 | 3 | 2 | 4 | 1 | 0 | 0 | 1 | 0 | 0 | 0 | 0 | 0 | 0 | 0 | 0 | 0 | 0 | 0 |
| Bt_G25-95 | 0.623 | 0 | 1 | 2 | 0 | 3 | 2 | 4 | 1 | 0 | 0 | 1 | 0 | 0 | 0 | 0 | 0 | 0 | 0 | 0 | 0 | 0 | 0 |
| Bf_G25-26 | 0.429 | 0 | 1 | 1 | 0 | 6 | 3 | 0 | 0 | 0 | 0 | 0 | 0 | 0 | 0 | 0 | 0 | 0 | 0 | 0 | 0 | 0 | 0 |
| Bf_G25-58 | 0.449 | 0 | 1 | 1 | 0 | 3 | 1 | 0 | 0 | 0 | 0 | 0 | 0 | 0 | 0 | 0 | 0 | 0 | 0 | 0 | 0 | 0 | 0 |
| Bf_G25-67 | 0.378 | 0 | 1 | 1 | 0 | 3 | 1 | 0 | 0 | 0 | 0 | 0 | 0 | 0 | 0 | 0 | 0 | 0 | 0 | 0 | 0 | 0 | 0 |
| Bf_G25-69 | 0.458 | 0 | 1 | 1 | 0 | 4 | 1 | 0 | 0 | 0 | 0 | 0 | 0 | 0 | 0 | 0 | 0 | 0 | 0 | 0 | 0 | 0 | 0 |
| Bf_G25-70 | 0.44 | 0 | 1 | 1 | 0 | 4 | 1 | 0 | 0 | 0 | 0 | 0 | 0 | 0 | 0 | 0 | 0 | 0 | 0 | 0 | 0 | 0 | 0 |
| Bf_G25-72 | 0.392 | 0 | 1 | 1 | 0 | 4 | 1 | 0 | 0 | 0 | 0 | 0 | 0 | 0 | 0 | 0 | 0 | 0 | 0 | 0 | 0 | 0 | 0 |
| Bf_G25-73 | 0.294 | 0 | 1 | 1 | 0 | 3 | 1 | 0 | 0 | 0 | 0 | 0 | 0 | 0 | 0 | 0 | 0 | 0 | 0 | 0 | 0 | 0 | 0 |
| Fbp_G25-29 | 0.359 | 0 | 1 | 1 | 0 | 3 | 4 | 0 | 0 | 0 | 0 | 0 | 0 | 0 | 0 | 0 | 0 | 0 | 0 | 0 | 0 | 0 | 0 |
| Bbl_G25-129 | 0.481 | 0 | 1 | 1 | 0 | 3 | 1 | 0 | 0 | 0 | 0 | 0 | 0 | 0 | 0 | 0 | 0 | 0 | 0 | 0 | 0 | 0 | 0 |
| Note: Degradation mechanism of combination of putative Bmp1 and Bae16 (subtype 2), both of which may digest proteins from intestine of nematodes; factors except putative Bmp1 and Bae16 are present or absent at the same column with a difference of number. This putative mechanism is shown as Fig. 3C. | | | | | | | | | | | | | | | | | | | | | | | |
| Type 1 of putative degradation mechanisms (cuticle) | | | | | | | | | | | | | | | | | | | | | | | |
| strains | c/10 | Bace16 | PurL | Eca1 | chitinase | BLG4 | Enp | Bmp1 | Bae16 | Thu | Nel | ColB | Cry5B | Cry5C | Cry5E | Cry12A | Cry13A | Cry14A | Cry21A | Cry21B | Cry6A | Cry6B | Cry55A |
| Bf_G25-27 | 0.561 | 0 | 1 | 1 | 0 | 5 | 3 | 0 | 0 | 0 | 0 | 0 | 0 | 0 | 0 | 0 | 0 | 0 | 0 | 0 | 0 | 0 | 0 |
| Bf_G25-55 | 0.722 | 0 | 1 | 1 | 0 | 3 | 1 | 0 | 0 | 0 | 0 | 0 | 0 | 0 | 0 | 0 | 0 | 0 | 0 | 0 | 0 | 0 | 0 |
| Bf_G25-56 | 0.702 | 0 | 1 | 1 | 0 | 3 | 1 | 0 | 0 | 0 | 0 | 0 | 0 | 0 | 0 | 0 | 0 | 0 | 0 | 0 | 0 | 0 | 0 |
| Fba_G25-54 | 0.721 | 0 | 1 | 1 | 0 | 6 | 2 | 0 | 0 | 0 | 0 | 0 | 0 | 0 | 0 | 0 | 0 | 0 | 0 | 0 | 0 | 0 | 0 |
| Pbp_G25-124 | 0.615 | 0 | 1 | 1 | 0 | 2 | 1 | 0 | 0 | 0 | 0 | 0 | 0 | 0 | 0 | 0 | 0 | 0 | 0 | 0 | 0 | 0 | 0 |
| Bbl_G25-128 | 0.574 | 0 | 1 | 1 | 0 | 3 | 1 | 0 | 0 | 0 | 0 | 0 | 0 | 0 | 0 | 0 | 0 | 0 | 0 | 0 | 0 | 0 | 0 |
| Bbl_G25-130 | 0.615 | 0 | 1 | 1 | 0 | 3 | 1 | 0 | 0 | 0 | 0 | 0 | 0 | 0 | 0 | 0 | 0 | 0 | 0 | 0 | 0 | 0 | 0 |
| Bbl_G25-131 | 0.615 | 0 | 1 | 1 | 0 | 3 | 1 | 0 | 0 | 0 | 0 | 0 | 0 | 0 | 0 | 0 | 0 | 0 | 0 | 0 | 0 | 0 | 0 |
| Bbb_G25-125 | 0.571 | 0 | 1 | 1 | 0 | 4 | 1 | 0 | 0 | 0 | 0 | 0 | 0 | 0 | 0 | 0 | 0 | 0 | 0 | 0 | 0 | 0 | 0 |
| Bbb_G25-137 | 0.586 | 0 | 1 | 1 | 0 | 3 | 1 | 0 | 0 | 0 | 0 | 0 | 0 | 0 | 0 | 0 | 0 | 0 | 0 | 0 | 0 | 0 | 0 |
| Bp_G25-37 | 0.465 | 0 | 1 | 1 | 0 | 6 | 0 | 0 | 0 | 0 | 0 | 0 | 0 | 0 | 0 | 0 | 0 | 0 | 0 | 0 | 0 | 0 | 0 |
| Bp_G25-38 | 0.432 | 0 | 1 | 1 | 0 | 6 | 0 | 0 | 0 | 0 | 0 | 0 | 0 | 0 | 0 | 0 | 0 | 0 | 0 | 0 | 0 | 0 | 0 |
| Bp_G25-63 | 0.275 | 0 | 1 | 1 | 0 | 6 | 0 | 0 | 0 | 0 | 0 | 0 | 0 | 0 | 0 | 0 | 0 | 0 | 0 | 0 | 0 | 0 | 0 |
| Bp_G25-64 | 0.188 | 0 | 1 | 1 | 0 | 6 | 0 | 0 | 0 | 0 | 0 | 0 | 0 | 0 | 0 | 0 | 0 | 0 | 0 | 0 | 0 | 0 | 0 |
| Bst_G25-119 | 0.183 | 0 | 1 | 1 | 0 | 5 | 0 | 0 | 0 | 0 | 0 | 0 | 0 | 0 | 0 | 0 | 0 | 0 | 0 | 0 | 0 | 0 | 0 |
| Lbs_G25-111 | 0.18 | 0 | 1 | 1 | 0 | 1 | 0 | 0 | 0 | 0 | 0 | 0 | 0 | 0 | 0 | 0 | 0 | 0 | 0 | 0 | 0 | 0 | 0 |
| Lbs_G25-112 | 0.246 | 0 | 1 | 1 | 0 | 1 | 0 | 0 | 0 | 0 | 0 | 0 | 0 | 0 | 0 | 0 | 0 | 0 | 0 | 0 | 0 | 0 | 0 |
| Lbs_G25-114 | 0.374 | 0 | 1 | 1 | 0 | 1 | 0 | 0 | 0 | 0 | 0 | 0 | 0 | 0 | 0 | 0 | 0 | 0 | 0 | 0 | 0 | 0 | 0 |
| Note: Degradation mechanism of putative Enp, which may digest proteins from cuticle of nematodes; factors except putative Enp are present or absent at the same column with a difference of number. Putative Bae16 is absent. This putative mechanism is shown as Fig. 3F (right). | | | | | | | | | | | | | | | | | | | | | | | |
| Type 1 of putative degradation mechanisms (subtype 1 of combination, intestine and cuticle) | | | | | | | | | | | | | | | | | | | | | | | |
| strains | c/10 | Bace16 | PurL | Eca1 | chitinase | BLG4 | Enp | Bmp1 | Bae16 | Thu | Nel | ColB | Cry5B | Cry5C | Cry5E | Cry12A | Cry13A | Cry14A | Cry21A | Cry21B | Cry6A | Cry6B | Cry55A |
| Bs_G25-83 | 0.85 | 1 | 1 | 1 | 0 | 2 | 1 | 0 | 1 | 0 | 0 | 0 | 0 | 0 | 0 | 0 | 0 | 0 | 0 | 0 | 0 | 0 | 0 |
| Bs_G25-31 | 0.56 | 1 | 1 | 1 | 0 | 2 | 1 | 0 | 1 | 0 | 0 | 0 | 0 | 0 | 0 | 0 | 0 | 0 | 0 | 0 | 0 | 0 | 0 |
| Bs_G25-40 | 0.663 | 1 | 1 | 1 | 0 | 2 | 1 | 0 | 1 | 0 | 0 | 0 | 0 | 0 | 0 | 0 | 0 | 0 | 0 | 0 | 0 | 0 | 0 |
| Bs_G25-135 | 0.588 | 1 | 1 | 1 | 0 | 3 | 1 | 0 | 1 | 0 | 0 | 0 | 0 | 0 | 0 | 0 | 0 | 0 | 0 | 0 | 0 | 0 | 0 |
| Bf_G25-26 | 0.429 | 0 | 1 | 1 | 0 | 6 | 3 | 0 | 0 | 0 | 0 | 0 | 0 | 0 | 0 | 0 | 0 | 0 | 0 | 0 | 0 | 0 | 0 |
| Bf_G25-58 | 0.449 | 0 | 1 | 1 | 0 | 3 | 1 | 0 | 0 | 0 | 0 | 0 | 0 | 0 | 0 | 0 | 0 | 0 | 0 | 0 | 0 | 0 | 0 |
| Bf_G25-67 | 0.378 | 0 | 1 | 1 | 0 | 3 | 1 | 0 | 0 | 0 | 0 | 0 | 0 | 0 | 0 | 0 | 0 | 0 | 0 | 0 | 0 | 0 | 0 |
| Bf_G25-69 | 0.458 | 0 | 1 | 1 | 0 | 4 | 1 | 0 | 0 | 0 | 0 | 0 | 0 | 0 | 0 | 0 | 0 | 0 | 0 | 0 | 0 | 0 | 0 |
| Bf_G25-70 | 0.44 | 0 | 1 | 1 | 0 | 4 | 1 | 0 | 0 | 0 | 0 | 0 | 0 | 0 | 0 | 0 | 0 | 0 | 0 | 0 | 0 | 0 | 0 |
| Bf_G25-72 | 0.392 | 0 | 1 | 1 | 0 | 4 | 1 | 0 | 0 | 0 | 0 | 0 | 0 | 0 | 0 | 0 | 0 | 0 | 0 | 0 | 0 | 0 | 0 |
| Bf_G25-73 | 0.294 | 0 | 1 | 1 | 0 | 3 | 1 | 0 | 0 | 0 | 0 | 0 | 0 | 0 | 0 | 0 | 0 | 0 | 0 | 0 | 0 | 0 | 0 |
| Fbp_G25-29 | 0.359 | 0 | 1 | 1 | 0 | 3 | 4 | 0 | 0 | 0 | 0 | 0 | 0 | 0 | 0 | 0 | 0 | 0 | 0 | 0 | 0 | 0 | 0 |
| Bbl_G25-129 | 0.481 | 0 | 1 | 1 | 0 | 3 | 1 | 0 | 0 | 0 | 0 | 0 | 0 | 0 | 0 | 0 | 0 | 0 | 0 | 0 | 0 | 0 | 0 |
| Note: Combination of Trojan horse mechanism and putative Enp; factors except putative Bace16 and Bae16 are present or absent at the same column with a difference of number; putative Enp is present with different number. This putative mechanism is shown as Fig. 3E (right). | | | | | | | | | | | | | | | | | | | | | | | |
| Type 1 of putative degradation mechanisms (subtype 2 of combination, intestine and cuticle) | | | | | | | | | | | | | | | | | | | | | | | |
| strains | c/10 | Bace16 | PurL | Eca1 | chitinase | BLG4 | Enp | Bmp1 | Bae16 | Thu | Nel | ColB | Cry5B | Cry5C | Cry5E | Cry12A | Cry13A | Cry14A | Cry21A | Cry21B | Cry6A | Cry6B | Cry55A |
| Bs_G25-83 | 0.85 | 1 | 1 | 1 | 0 | 2 | 1 | 0 | 1 | 0 | 0 | 0 | 0 | 0 | 0 | 0 | 0 | 0 | 0 | 0 | 0 | 0 | 0 |
| Bs_G25-31 | 0.56 | 1 | 1 | 1 | 0 | 2 | 1 | 0 | 1 | 0 | 0 | 0 | 0 | 0 | 0 | 0 | 0 | 0 | 0 | 0 | 0 | 0 | 0 |
| Bs_G25-40 | 0.663 | 1 | 1 | 1 | 0 | 2 | 1 | 0 | 1 | 0 | 0 | 0 | 0 | 0 | 0 | 0 | 0 | 0 | 0 | 0 | 0 | 0 | 0 |
| Bs_G25-61 | 0.409 | 1 | 1 | 1 | 0 | 2 | 1 | 0 | 1 | 0 | 0 | 0 | 0 | 0 | 0 | 0 | 0 | 0 | 0 | 0 | 0 | 0 | 0 |
| Bs_G25-90 | 0.426 | 1 | 1 | 1 | 0 | 2 | 1 | 0 | 1 | 0 | 0 | 0 | 0 | 0 | 0 | 0 | 0 | 0 | 0 | 0 | 0 | 0 | 0 |
| Bs_G25-135 | 0.588 | 1 | 1 | 1 | 0 | 3 | 1 | 0 | 1 | 0 | 0 | 0 | 0 | 0 | 0 | 0 | 0 | 0 | 0 | 0 | 0 | 0 | 0 |
| Bam_G25-110 | 0.367 | 1 | 1 | 1 | 0 | 3 | 0 | 0 | 1 | 0 | 0 | 0 | 0 | 0 | 0 | 0 | 0 | 0 | 0 | 0 | 0 | 0 | 0 |
| Bs_G25-60 | 0.357 | 1 | 1 | 1 | 0 | 2 | 0 | 0 | 1 | 0 | 0 | 0 | 0 | 0 | 0 | 0 | 0 | 0 | 0 | 0 | 0 | 0 | 0 |
| Bv_G25-127 | 0.443 | 1 | 1 | 1 | 0 | 2 | 0 | 0 | 1 | 0 | 0 | 0 | 0 | 0 | 0 | 0 | 0 | 0 | 0 | 0 | 0 | 0 | 0 |
| Note: Combination of putative Enp and Bae16; factors except putative Enp are present or absent at the same column with a difference of number; putative Bae16 is present with different number. This putative mechanism is shown as Fig. 3F (left). | | | | | | | | | | | | | | | | | | | | | | | |
| Type 1 of putative degradation mechanisms (subtype 3 of combination, intestine and cuticle) | | | | | | | | | | | | | | | | | | | | | | | |
| strains | c/10 | Bace16 | PurL | Eca1 | chitinase | BLG4 | Enp | Bmp1 | Bae16 | Thu | Nel | ColB | Cry5B | Cry5C | Cry5E | Cry12A | Cry13A | Cry14A | Cry21A | Cry21B | Cry6A | Cry6B | Cry55A |
| Bt_G25-13 | 0.833 | 0 | 1 | 2 | 0 | 2 | 1 | 1 | 1 | 0 | 0 | 1 | 0 | 0 | 0 | 0 | 0 | 0 | 0 | 0 | 0 | 0 | 0 |
| Bt_G25-94 | 0.558 | 0 | 1 | 2 | 0 | 3 | 2 | 4 | 1 | 0 | 0 | 1 | 0 | 0 | 0 | 0 | 0 | 0 | 0 | 0 | 0 | 0 | 0 |
| Bt_G25-95 | 0.623 | 0 | 1 | 2 | 0 | 3 | 2 | 4 | 1 | 0 | 0 | 1 | 0 | 0 | 0 | 0 | 0 | 0 | 0 | 0 | 0 | 0 | 0 |
| Bar_G25-109 | 0.425 | 0 | 1 | 1 | 0 | 1 | 0 | 0 | 1 | 0 | 0 | 0 | 0 | 0 | 0 | 0 | 0 | 0 | 0 | 0 | 0 | 0 | 0 |
| Pbl_G25-75 | 0.506 | 0 | 1 | 1 | 0 | 2 | 0 | 0 | 3 | 0 | 0 | 0 | 0 | 0 | 0 | 0 | 0 | 0 | 0 | 0 | 0 | 0 | 0 |
| Bba_G25-57 | 0.431 | 0 | 1 | 1 | 0 | 3 | 0 | 0 | 1 | 0 | 0 | 0 | 0 | 0 | 0 | 0 | 0 | 0 | 0 | 0 | 0 | 0 | 0 |
| Note: Combination of putative Enp and Bmp1; factors except putative Enp and Bmp1 are present or absent at the same column with a difference of number. This putative mechanism is shown as Fig. 3D. | | | | | | | | | | | | | | | | | | | | | | | |
| Type 1 of putative degradation mechanisms (subtype 4 of combination, intestine and cuticle) | | | | | | | | | | | | | | | | | | | | | | | |
| strains | c/10 | Bace16 | PurL | Eca1 | chitinase | BLG4 | Enp | Bmp1 | Bae16 | Thu | Nel | ColB | Cry5B | Cry5C | Cry5E | Cry12A | Cry13A | Cry14A | Cry21A | Cry21B | Cry6A | Cry6B | Cry55A |
| Bt_G25-13 | 0.833 | 0 | 1 | 2 | 0 | 2 | 1 | 1 | 1 | 0 | 0 | 1 | 0 | 0 | 0 | 0 | 0 | 0 | 0 | 0 | 0 | 0 | 0 |
| Bt_G25-94 | 0.558 | 0 | 1 | 2 | 0 | 3 | 2 | 4 | 1 | 0 | 0 | 1 | 0 | 0 | 0 | 0 | 0 | 0 | 0 | 0 | 0 | 0 | 0 |
| Bt_G25-95 | 0.623 | 0 | 1 | 2 | 0 | 3 | 2 | 4 | 1 | 0 | 0 | 1 | 0 | 0 | 0 | 0 | 0 | 0 | 0 | 0 | 0 | 0 | 0 |
| Bp_G25-37 | 0.465 | 0 | 1 | 1 | 0 | 6 | 0 | 0 | 0 | 0 | 0 | 0 | 0 | 0 | 0 | 0 | 0 | 0 | 0 | 0 | 0 | 0 | 0 |
| Bp_G25-38 | 0.432 | 0 | 1 | 1 | 0 | 6 | 0 | 0 | 0 | 0 | 0 | 0 | 0 | 0 | 0 | 0 | 0 | 0 | 0 | 0 | 0 | 0 | 0 |
| Bp_G25-63 | 0.275 | 0 | 1 | 1 | 0 | 6 | 0 | 0 | 0 | 0 | 0 | 0 | 0 | 0 | 0 | 0 | 0 | 0 | 0 | 0 | 0 | 0 | 0 |
| Bp_G25-64 | 0.188 | 0 | 1 | 1 | 0 | 6 | 0 | 0 | 0 | 0 | 0 | 0 | 0 | 0 | 0 | 0 | 0 | 0 | 0 | 0 | 0 | 0 | 0 |
| Bst_G25-119 | 0.183 | 0 | 1 | 1 | 0 | 5 | 0 | 0 | 0 | 0 | 0 | 0 | 0 | 0 | 0 | 0 | 0 | 0 | 0 | 0 | 0 | 0 | 0 |
| Lbs_G25-111 | 0.18 | 0 | 1 | 1 | 0 | 1 | 0 | 0 | 0 | 0 | 0 | 0 | 0 | 0 | 0 | 0 | 0 | 0 | 0 | 0 | 0 | 0 | 0 |
| Lbs_G25-112 | 0.246 | 0 | 1 | 1 | 0 | 1 | 0 | 0 | 0 | 0 | 0 | 0 | 0 | 0 | 0 | 0 | 0 | 0 | 0 | 0 | 0 | 0 | 0 |
| Lbs_G25-114 | 0.374 | 0 | 1 | 1 | 0 | 1 | 0 | 0 | 0 | 0 | 0 | 0 | 0 | 0 | 0 | 0 | 0 | 0 | 0 | 0 | 0 | 0 | 0 |
| Note: Combination of putative Enp, Bmp1 and Bae16; factors except putative Enp, Bmp1 and Bae16 are present or absent at the same column with a difference of number. Putative chitinases are absent. This putative mechanism is shown as Fig. 3G (right). | | | | | | | | | | | | | | | | | | | | | | | |
| Type 2 of putative degradation mechanisms (combination of chitinases and proteases) | | | | | | | | | | | | | | | | | | | | | | | |
| strains | c/10 | Bace16 | PurL | Eca1 | chitinase | BLG4 | Enp | Bmp1 | Bae16 | Thu | Nel | ColB | Cry5B | Cry5C | Cry5E | Cry12A | Cry13A | Cry14A | Cry21A | Cry21B | Cry6A | Cry6B | Cry55A |
| Bt_G25-1 | 0.56 | 0 | 1 | 2 | 1 | 3 | 1 | 4 | 1 | 0 | 0 | 1 | 0 | 0 | 0 | 0 | 0 | 0 | 0 | 0 | 0 | 0 | 0 |
| Bt_G25-6 | 0.536 | 0 | 1 | 2 | 1 | 3 | 1 | 2 | 1 | 0 | 0 | 1 | 0 | 0 | 0 | 0 | 0 | 0 | 0 | 0 | 0 | 0 | 0 |
| Bt_G25-39 | 0.664 | 0 | 1 | 2 | 1 | 3 | 1 | 4 | 1 | 0 | 0 | 1 | 0 | 0 | 0 | 0 | 0 | 0 | 0 | 0 | 0 | 0 | 0 |
| Bt_G25-76 | 0.85 | 0 | 1 | 2 | 1 | 4 | 1 | 4 | 1 | 0 | 0 | 1 | 0 | 0 | 0 | 0 | 0 | 0 | 0 | 0 | 0 | 0 | 0 |
| Bt_G25-93 | 0.706 | 0 | 1 | 2 | 1 | 3 | 1 | 2 | 1 | 0 | 0 | 1 | 0 | 0 | 0 | 0 | 0 | 0 | 0 | 0 | 0 | 0 | 0 |
| Bt_G25-97 | 0.848 | 0 | 1 | 2 | 1 | 3 | 1 | 2 | 1 | 0 | 0 | 1 | 0 | 0 | 0 | 0 | 0 | 0 | 0 | 0 | 0 | 0 | 0 |
| Bc_G25-65 | 0.729 | 0 | 1 | 2 | 1 | 2 | 1 | 3 | 1 | 0 | 0 | 1 | 0 | 0 | 0 | 0 | 0 | 0 | 0 | 0 | 0 | 0 | 0 |
| Bc_G25-66 | 0.666 | 0 | 1 | 2 | 1 | 2 | 1 | 3 | 1 | 0 | 0 | 1 | 0 | 0 | 0 | 0 | 0 | 0 | 0 | 0 | 0 | 0 | 0 |
| Bc_G25-86 | 0.85 | 0 | 1 | 2 | 1 | 4 | 1 | 2 | 1 | 0 | 0 | 1 | 0 | 0 | 0 | 0 | 0 | 0 | 0 | 0 | 0 | 0 | 0 |
| Bw_G25-84 | 0.769 | 0 | 1 | 2 | 1 | 1 | 1 | 1 | 1 | 0 | 0 | 1 | 0 | 0 | 0 | 0 | 0 | 0 | 0 | 0 | 0 | 0 | 0 |
| Bto_G25-88 | 0.646 | 0 | 1 | 2 | 1 | 3 | 1 | 3 | 1 | 0 | 0 | 1 | 0 | 0 | 0 | 0 | 0 | 0 | 0 | 0 | 0 | 0 | 0 |
| Bto_G25-123 | 0.525 | 0 | 1 | 2 | 1 | 2 | 1 | 4 | 1 | 0 | 0 | 1 | 0 | 0 | 0 | 0 | 0 | 0 | 0 | 0 | 0 | 0 | 0 |
| Ble_G25-134 | 0.462 | 0 | 1 | 1 | 1 | 2 | 0 | 0 | 0 | 0 | 0 | 0 | 0 | 0 | 0 | 0 | 0 | 0 | 0 | 0 | 0 | 0 | 0 |
| Bg_G25-74 | 0.276 | 0 | 1 | 1 | 1 | 1 | 0 | 0 | 0 | 0 | 0 | 0 | 0 | 0 | 0 | 0 | 0 | 0 | 0 | 0 | 0 | 0 | 0 |
| Pba_G25-118 | 0.086 | 0 | 1 | 1 | 2 | 4 | 0 | 0 | 0 | 0 | 0 | 0 | 0 | 0 | 0 | 0 | 0 | 0 | 0 | 0 | 0 | 0 | 0 |
| Note: Combination of putative Enp, Bmp1, Bae16 and chitinases; factors except putative Enp, Bmp1 and Bae16 are present or absent at the same column with a difference of number. Putative chitinases are present. This putative mechanism is shown as Fig. 3G (left). | | | | | | | | | | | | | | | | | | | | | | | |

Note: Strain names were abbreviated as follows: Bt for *B. thuringiensis*, Bc for *B. cereus*, Bw for *B. weihenstephanensis*, Bto for *B. toyonensis*, Bmy for *B. mycoides*, Bam for *B. amyloliquefaciens*, Bs for *B. subtilis*, Bli for *B. licheniformis*, Bat for *B. atrophaeus*, Bv for *B. vallismortis*, Bp for *B. pumilus*, Bf for *B. firmus*, Bm for *B. megaterium*, Bar for *B. aryabhattai*, Bl for *B. lehensis*, Bst for *B. stratosphericus*, Be for *B. endophyticus*, Bmu for *B. muralis*, Bg for *B. galactosidilyticus*, Fbp for *F. phosphorivorans*, Fba for *F. arsenicus*, Lbs for *L. sphaericus*, Lbf for *L. fusiformis*, Pbl for *P. larvae*, Pba for *P. alvei*, Pbd for *P. dendritiformis*, Pbp for *P. polymyxa*, Bbl for *B. laterosporus*, Bbb for *B. brevis* and Bba for *B. agri*.

**Table S9** Domains of putative chitinases in genomes of nematicidal spore-forming Bacilli

|  |  |  | protein_id | List of domain hits | Accession | Interval | E-value | Description |
| --- | --- | --- | --- | --- | --- | --- | --- | --- |
| **virulence chitinases and their homologies** | **group A** | | **G25-39_01563** | GH18_chitinase | cd06548 | 41-450 | 5.74E-151 | The GH18 (glycosyl hydrolases, family 18) type II chitinases hydrolyze chitin, an abundant polymer of N-acetylglucosamine and have been identified in bacteria, fungi, insects, plants, viruses, and protozoan parasites. The structure of this domain is an eight-stranded alpha/beta barrel with a pronounced active-site cleft at the C-terminal end of the beta-barrel. |
|  |  |  |  | FN3 | cd00063 | 485-567 | 6.27E-13 | Fibronectin type 3 domain; One of three types of internal repeats found in the plasma protein fibronectin. Its tenth fibronectin type III repeat contains an RGD cell recognition sequence in a flexible loop between 2 strands. Approximately 2% of all animal proteins contain the FN3 repeat; including extracellular and intracellular proteins, membrane spanning cytokine receptors, growth hormone receptors, tyrosine phosphatase receptors, and adhesion molecules. FN3-like domains are also found in bacterial glycosyl hydrolases. |
|  |  |  |  | CBM_2 | pfam00553 | 580-661 | 2.80E-20 | Two tryptophan residues are involved in cellulose binding. Cellulose binding domain found in bacteria |
|  |  |  |  | Glyco_18 | smart00636 | 40-450 | 2.48E-135 |  |
|  |  |  | G25-96_01701 | GH18_chitinase | cd06548 | 41-450 | 1.09E-151 |  |
|  |  |  |  | FN3 | cd00063 | 485-567 | 1.77E-12 |  |
|  |  |  |  | CBM_2 | pfam00553 | 580-661 | 2.29E-20 |  |
|  |  |  |  | Glyco_18 | smart00636 | 40-450 | 8.99E-136 |  |
|  |  |  | G25-91_06230 | GH18_chitinase | cd06548 | 41-450 | 6.75E-152 |  |
|  |  |  |  | FN3 | cd00063 | 485-567 | 1.33E-12 |  |
|  |  |  |  | CBM_2 | pfam00553 | 580-661 | 9.63E-21 |  |
|  |  |  |  | Glyco_18 | smart00636 | 40-450 | 5.66E-136 |  |
|  |  |  | G25-8_00541 | GH18_chitinase | cd06548 | 41-450 | 1.09E-151 |  |
|  |  |  |  | FN3 | cd00063 | 485-567 | 1.77E-12 |  |
|  |  |  |  | CBM_2 | pfam00553 | 580-661 | 2.29E-20 |  |
|  |  |  |  | Glyco_18 | smart00636 | 40-450 | 8.99E-136 |  |
|  |  |  | G25-50_03945 | GH18_chitinase | cd06548 | 41-450 | 7.68E-152 |  |
|  |  |  |  | FN3 | cd00063 | 485-567 | 5.81E-13 |  |
|  |  |  |  | CBM_2 | pfam00553 | 580-661 | 1.00E-20 |  |
|  |  |  |  | Glyco_18 | smart00636 | 40-450 | 6.04E-136 |  |
|  |  |  | G25-44_02664 | GH18_chitinase | cd06548 | 41-450 | 6.26E-152 |  |
|  |  |  |  | FN3 | cd00063 | 485-567 | 1.76E-13 |  |
|  |  |  |  | CBM_2 | pfam00553 | 580-661 | 1.09E-20 |  |
|  |  |  |  | Glyco_18 | smart00636 | 40-450 | 4.92E-136 |  |
|  |  |  | G25-17_00517 | GH18_chitinase | cd06548 | 41-450 | 7.68E-152 |  |
|  |  |  |  | FN3 | cd00063 | 485-567 | 5.81E-13 |  |
|  |  |  |  | CBM_2 | pfam00553 | 580-661 | 1.00E-20 |  |
|  |  |  |  | Glyco_18 | smart00636 | 40-450 | 6.04E-136 |  |
|  |  |  | G25-120_02224 | GH18_chitinase | cd06548 | 41-450 | 6.46E-152 |  |
|  |  |  |  | FN3 | cd00063 | 485-567 | 6.84E-13 |  |
|  |  |  |  | CBM_2 | pfam00553 | 580-661 | 1.24E-20 |  |
|  |  |  |  | Glyco_18 | smart00636 | 40-450 | 5.19E-136 |  |
|  |  |  | G25-1_00564 | GH18_chitinase | cd06548 | 41-450 | 1.09E-151 |  |
|  |  |  |  | FN3 | cd00063 | 485-567 | 1.77E-12 |  |
|  |  |  |  | CBM_2 | pfam00553 | 580-661 | 2.29E-20 |  |
|  |  |  |  | Glyco_18 | smart00636 | 40-450 | 8.99E-136 |  |
|  |  |  | G25-123_00589 | GH18_chitinase | cd06548 | 41-450 | 6.46E-152 |  |
|  |  |  |  | FN3 | cd00063 | 485-567 | 6.84E-13 |  |
|  |  |  |  | CBM_2 | pfam00553 | 580-661 | 1.24E-20 |  |
|  |  |  |  | Glyco_18 | smart00636 | 40-450 | 5.19E-136 |  |
|  |  |  | G25-43_00331 | GH18_chitinase | cd06548 | 41-450 | 7.68E-152 |  |
|  |  |  |  | FN3 | cd00063 | 485-567 | 5.81E-13 |  |
|  |  |  |  | CBM_2 | pfam00553 | 580-661 | 1.00E-20 |  |
|  |  |  |  | Glyco_18 | smart00636 | 40-450 | 6.04E-136 |  |
|  |  |  | G25-49_04743 | GH18_chitinase | cd06548 | 41-450 | 7.68E-152 |  |
|  |  |  |  | FN3 | cd00063 | 485-567 | 5.81E-13 |  |
|  |  |  |  | CBM_2 | pfam00553 | 580-661 | 1.00E-20 |  |
|  |  |  |  | Glyco_18 | smart00636 | 40-450 | 6.04E-136 |  |
|  |  |  | G25-103_02791 | GH18_chitinase | cd06548 | 41-450 | 2.08E-151 |  |
|  |  |  |  | FN3 | cd00063 | 485-567 | 6.84E-13 |  |
|  |  |  |  | CBM_2 | pfam00553 | 580-661 | 1.03E-20 |  |
|  |  |  |  | Glyco_18 | smart00636 | 40-450 | 1.23E-135 |  |
|  |  |  | G25-78_05350 | GH18_chitinase | cd06548 | 41-450 | 6.75E-152 |  |
|  |  |  |  | FN3 | cd00063 | 485-567 | 1.33E-12 |  |
|  |  |  |  | CBM_2 | pfam00553 | 580-661 | 9.63E-21 |  |
|  |  |  |  | Glyco_18 | smart00636 | 40-450 | 5.66E-136 |  |
|  |  |  | G25-121_00274 | GH18_chitinase | cd06548 | 41-450 | 6.46E-152 |  |
|  |  |  |  | FN3 | cd00063 | 485-567 | 6.84E-13 |  |
|  |  |  |  | CBM_2 | pfam00553 | 580-661 | 1.24E-20 |  |
|  |  |  |  | Glyco_18 | smart00636 | 40-450 | 5.19E-136 |  |
|  |  |  | G25-41_02320 | GH18_chitinase | cd06548 | 41-450 | 7.68E-152 |  |
|  |  |  |  | FN3 | cd00063 | 485-567 | 5.81E-13 |  |
|  |  |  |  | CBM_2 | pfam00553 | 580-661 | 1.00E-20 |  |
|  |  |  |  | Glyco_18 | smart00636 | 40-450 | 6.04E-136 |  |
|  |  |  | G25-47_06165 | GH18_chitinase | cd06548 | 41-450 | 6.90E-152 |  |
|  |  |  |  | FN3 | cd00063 | 485-567 | 5.94E-12 |  |
|  |  |  |  | CBM_2 | pfam00553 | 580-661 | 8.33E-21 |  |
|  |  |  |  | Glyco_18 | smart00636 | 40-450 | 5.66E-136 |  |
|  |  |  | G25-51_05828 | GH18_chitinase | cd06548 | 41-450 | 7.68E-152 |  |
|  |  |  |  | FN3 | cd00063 | 485-567 | 5.81E-13 |  |
|  |  |  |  | CBM_2 | pfam00553 | 580-661 | 1.00E-20 |  |
|  |  |  |  | Glyco_18 | smart00636 | 40-450 | 6.04E-136 |  |
|  |  |  | G25-87_02278 | GH18_chitinase | cd06548 | 41-450 | 6.75E-152 |  |
|  |  |  |  | FN3 | cd00063 | 485-567 | 1.33E-12 |  |
|  |  |  |  | CBM_2 | pfam00553 | 580-661 | 9.63E-21 |  |
|  |  |  |  | Glyco_18 | smart00636 | 40-450 | 5.66E-136 |  |
|  |  |  | G25-9_05283 | GH18_chitinase | cd06548 | 41-450 | 6.90E-152 |  |
|  |  |  |  | FN3 | cd00063 | 485-567 | 5.94E-12 |  |
|  |  |  |  | CBM_2 | pfam00553 | 580-661 | 8.33E-21 |  |
|  |  |  |  | Glyco_18 | smart00636 | 40-450 | 5.66E-136 |  |
|  |  |  | G25-106_00361 | GH18_chitinase | cd06548 | 41-450 | 3.38E-151 |  |
|  |  |  |  | FN3 | cd00063 | 485-567 | 7.59E-13 |  |
|  |  |  |  | CBM_2 | pfam00553 | 580-661 | 1.23E-20 |  |
|  |  |  |  | Glyco_18 | smart00636 | 40-450 | 1.93E-135 |  |
|  |  |  | G25-45_00649 | GH18_chitinase | cd06548 | 41-450 | 3.38E-151 |  |
|  |  |  |  | FN3 | cd00063 | 485-567 | 7.59E-13 |  |
|  |  |  |  | CBM_2 | pfam00553 | 580-661 | 1.23E-20 |  |
|  |  |  |  | Glyco_18 | smart00636 | 40-450 | 1.93E-135 |  |
|  |  |  | G25-48_02343 | GH18_chitinase | cd06548 | 41-450 | 3.38E-151 |  |
|  |  |  |  | FN3 | cd00063 | 485-567 | 6.59E-12 |  |
|  |  |  |  | CBM_2 | pfam00553 | 580-661 | 1.00E-20 |  |
|  |  |  |  | Glyco_18 | smart00636 | 40-450 | 1.74E-135 |  |
|  |  |  | G25-86_00642 | GH18_chitinase | cd06548 | 41-450 | 1.05E-150 |  |
|  |  |  |  | FN3 | cd00063 | 485-567 | 8.19E-13 |  |
|  |  |  |  | CBM_2 | pfam00553 | 580-661 | 1.46E-20 |  |
|  |  |  |  | Glyco_18 | smart00636 | 40-450 | 1.46E-134 |  |
|  |  |  | G25-11_00411 | GH18_chitinase | cd06548 | 41-450 | 6.90E-152 |  |
|  |  |  |  | FN3 | cd00063 | 485-567 | 9.10E-13 |  |
|  |  |  |  | CBM_2 | pfam00553 | 580-661 | 1.06E-20 |  |
|  |  |  |  | Glyco_18 | smart00636 | 40-450 | 5.36E-136 |  |
|  |  |  | G25-2_3520 | GH18_chitinase | cd06548 | 55-464 | 3.80E-152 |  |
|  |  |  |  | FN3 | cd00063 | 499-581 | 6.88E-13 |  |
|  |  |  |  | CBM_2 | pfam00553 | 594-675 | 6.16E-21 |  |
|  |  |  |  | Glyco_18 | smart00636 | 54-464 | 3.84E-136 |  |
|  |  |  | G25-76_02025 | GH18_chitinase | cd06548 | 41-450 | 6.90E-152 |  |
|  |  |  |  | FN3 | cd00063 | 485-567 | 9.10E-13 |  |
|  |  |  |  | CBM_2 | pfam00553 | 580-661 | 1.06E-20 |  |
|  |  |  |  | Glyco_18 | smart00636 | 40-450 | 5.36E-136 |  |
|  |  |  | G25-85_02496 | GH18_chitinase | cd06548 | 41-450 | 6.90E-152 |  |
|  |  |  |  | FN3 | cd00063 | 485-567 | 9.10E-13 |  |
|  |  |  |  | CBM_2 | pfam00553 | 580-661 | 1.06E-20 |  |
|  |  |  |  | Glyco_18 | smart00636 | 40-450 | 5.36E-136 |  |
|  |  |  | G25-18_03184 | GH18_chitinase | cd06548 | 41-450 | 2.31E-151 |  |
|  |  |  |  | FN3 | cd00063 | 485-567 | 3.40E-13 |  |
|  |  |  |  | CBM_2 | pfam00553 | 580-661 | 1.42E-20 |  |
|  |  |  |  | Glyco_18 | smart00636 | 40-450 | 5.86E-135 |  |
|  |  |  | G25-6_00291 | GH18_chitinase | cd06548 | 41-450 | 1.21E-151 |  |
|  |  |  |  | FN3 | cd00063 | 485-567 | 4.67E-13 |  |
|  |  |  |  | CBM_2 | pfam00553 | 580-661 | 1.37E-20 |  |
|  |  |  |  | Glyco_18 | smart00636 | 40-450 | 8.71E-136 |  |
|  |  |  | G25-93_04793 | GH18_chitinase | cd06548 | 41-450 | 8.94E-152 |  |
|  |  |  |  | FN3 | cd00063 | 485-567 | 8.27E-13 |  |
|  |  |  |  | CBM_2 | pfam00553 | 580-661 | 1.46E-20 |  |
|  |  |  |  | Glyco_18 | smart00636 | 40-450 | 4.37E-136 |  |
|  |  |  | G25-97_01545 | GH18_chitinase | cd06548 | 41-450 | 8.94E-152 |  |
|  |  |  |  | FN3 | cd00063 | 485-567 | 8.27E-13 |  |
|  |  |  |  | CBM_2 | pfam00553 | 580-661 | 1.46E-20 |  |
|  |  |  |  | Glyco_18 | smart00636 | 40-450 | 4.37E-136 |  |
|  |  |  | G25-104_02797 | GH18_chitinase | cd06548 | 41-450 | 1.21E-149 |  |
|  |  |  |  | FN3 | cd00063 | 485-567 | 3.39E-12 |  |
|  |  |  |  | CBM_2 | pfam00553 | 580-672 | 3.68E-20 |  |
|  |  |  |  | Glyco_18 | smart00636 | 40-450 | 4.24E-135 |  |
|  |  |  | G25-84_02309 | GH18_chitinase | cd06548 | 41-450 | 3.38E-150 |  |
|  |  |  |  | FN3 | cd00063 | 485-567 | 1.67E-09 |  |
|  |  |  |  | CBM_2 | pfam00553 | 580-672 | 6.09E-20 |  |
|  |  |  |  | Glyco_18 | smart00636 | 40-450 | 1.31E-135 |  |
|  |  |  | G25-105_03876 | GH18_chitinase | cd06548 | 41-450 | 4.83E-151 |  |
|  |  |  |  | FN3 | cd00063 | 485-567 | 6.23E-12 |  |
|  |  |  |  | CBM_2 | pfam00553 | 580-672 | 2.20E-20 |  |
|  |  |  |  | Glyco_18 | smart00636 | 40-450 | 2.64E-135 |  |
|  |  |  | G25-3_01402 | GH18_chitinase | cd06548 | 41-450 | 2.94E-152 |  |
|  |  |  |  | FN3 | cd00063 | 485-567 | 2.61E-11 |  |
|  |  |  |  | CBM_2 | pfam00553 | 580-671 | 5.59E-20 |  |
|  |  |  |  | Glyco_18 | smart00636 | 40-450 | 1.05E-135 |  |
|  |  |  | G25-88_01863 | GH18_chitinase | cd06548 | 41-450 | 3.93E-151 |  |
|  |  |  |  | FN3 | cd00063 | 485-567 | 9.46E-11 |  |
|  |  |  |  | CBM_2 | pfam00553 | 580-662 | 2.71E-19 |  |
|  |  |  |  | Glyco_18 | smart00636 | 40-450 | 1.40E-135 |  |
|  |  |  | G25-4_01222 | GH18_chitinase | cd06548 | 41-450 | 3.13E-151 |  |
|  |  |  |  | FN3 | cd00063 | 485-567 | 1.58E-11 |  |
|  |  |  |  | CBM_2 | pfam00553 | 580-661 | 1.56E-19 |  |
|  |  |  |  | Glyco_18 | smart00636 | 40-450 | 3.84E-136 |  |
|  |  |  | G25-46_02661 | GH18_chitinase | cd06548 | 41-450 | 1.77E-150 |  |
|  |  |  |  | FN3 | cd00063 | 485-567 | 1.74E-11 |  |
|  |  |  |  | CBM_2 | pfam00553 | 580-661 | 1.74E-19 |  |
|  |  |  |  | Glyco_18 | smart00636 | 40-450 | 1.56E-135 |  |
|  |  |  | G25-42_00062 | GH18_chitinase | cd06548 | 41-450 | 5.04E-152 |  |
|  |  |  |  | FN3 | cd00063 | 485-567 | 2.91E-10 |  |
|  |  |  |  | CBM_2 | pfam00553 | 580-662 | 1.64E-20 |  |
|  |  |  |  | Glyco_18 | smart00636 | 40-450 | 1.06E-135 |  |
|  |  |  | G25-53_02041 | GH18_chitinase | cd06548 | 41-450 | 2.75E-152 |  |
|  |  |  |  | FN3 | cd00063 | 485-567 | 3.08E-10 |  |
|  |  |  |  | CBM_2 | pfam00553 | 580-662 | 1.85E-20 |  |
|  |  |  |  | Glyco_18 | smart00636 | 40-450 | 9.39E-136 |  |
|  |  |  | G25-108_01921 | GH18_chitinase | cd06548 | 41-450 | 3.20E-153 |  |
|  |  |  |  | FN3 | cd00063 | 485-567 | 2.61E-11 |  |
|  |  |  |  | CBM_2 | pfam00553 | 580-671 | 5.27E-20 |  |
|  |  |  |  | Glyco_18 | smart00636 | 40-450 | 6.72E-136 |  |
|  |  |  | G25-5_01747 | GH18_chitinase | cd06548 | 41-450 | 4.62E-152 |  |
|  |  |  |  | FN3 | cd00063 | 485-567 | 1.17E-11 |  |
|  |  |  |  | CBM_2 | pfam00553 | 580-662 | 2.58E-19 |  |
|  |  |  |  | Glyco_18 | smart00636 | 40-450 | 1.55E-136 |  |
|  |  |  | G25-101_05211 | GH18_chitinase | cd06548 | 41-450 | 2.34E-152 |  |
|  |  |  |  | FN3 | cd00063 | 485-567 | 1.45E-11 |  |
|  |  |  |  | CBM_2 | pfam00553 | 580-662 | 1.67E-19 |  |
|  |  |  |  | Glyco_18 | smart00636 | 40-450 | 8.80E-136 |  |
|  |  |  | G25-102_00032 | GH18_chitinase | cd06548 | 41-450 | 2.34E-152 |  |
|  |  |  |  | FN3 | cd00063 | 485-567 | 1.45E-11 |  |
|  |  |  |  | CBM_2 | pfam00553 | 580-662 | 1.67E-19 |  |
|  |  |  |  | Glyco_18 | smart00636 | 40-450 | 8.80E-136 |  |
|  |  |  | G25-65_02960 | GH18_chitinase | cd06548 | 41-450 | 2.34E-152 |  |
|  |  |  |  | FN3 | cd00063 | 485-567 | 1.45E-11 |  |
|  |  |  |  | CBM_2 | pfam00553 | 580-662 | 1.67E-19 |  |
|  |  |  |  | Glyco_18 | smart00636 | 40-450 | 8.80E-136 |  |
|  |  |  | G25-66_03277 | GH18_chitinase | cd06548 | 41-450 | 2.34E-152 |  |
|  |  |  |  | FN3 | cd00063 | 485-567 | 1.45E-11 |  |
|  |  |  |  | CBM_2 | pfam00553 | 580-662 | 1.67E-19 |  |
|  |  |  |  | Glyco_18 | smart00636 | 40-450 | 8.80E-136 |  |
|  |  |  | G25-7_01566 | GH18_chitinase | cd06548 | 41-450 | 4.99E-152 |  |
|  |  |  |  | FN3 | cd00063 | 485-567 | 1.63E-11 |  |
|  |  |  |  | CBM_2 | pfam00553 | 580-662 | 1.82E-19 |  |
|  |  |  |  | Glyco_18 | smart00636 | 40-450 | 2.11E-135 |  |
|  | **group B** | **group B1** | **G25-134_00461** | GH18_chitinase | cd06548 | 37-424 | 1.47E-137 |  |
|  |  |  |  | ChiC_BD | cd12215 | 449-492 | 5.09E-13 | Chitin-binding domain of chitinase C of Streptomyces griseus and related proteins. Chitinase C is a family 19 chitinase, and consists of a N-terminal chitin binding domain and a C-terminal chitin-catalytic domain that effects degradation. Chitinases function in invertebrates in the degradation of old exoskeletons, in fungi to utilize chitin in cell walls, and in bacteria which use chitin as an energy source. ChiC contains the characteristic chitin-binding aromatic residues. |
|  |  |  |  | ChiC_BD | cd12215 | 505-545 | 5.10E-11 |  |
|  |  |  |  | ChiA | COG3325 | 20-434 | 3.18E-130 | Chitinase, GH18 family [Carbohydrate transport and metabolism] |
|  |  |  |  | COG3979 | COG3979 | 448-545 | 2.76E-14 | Chitodextrinase [Carbohydrate transport and metabolism] |
|  |  | **group B2** | **G25-122_03240** | GH18_chitinase | cd06548 | 38-428 | 9.88E-145 |  |
|  |  |  |  | ChiC_BD | cd12215 | 545-588 | 1.72E-15 |  |
|  |  |  |  | FN3 | cd00063 | 455-525 | 1.64E-13 |  |
|  |  |  |  | ChiA | COG3325 | 28-445 | 1.56E-159 |  |
|  |  |  |  | COG3979 | COG3979 | 456-592 | 2.80E-17 |  |
|  |  |  | G25-99_02709 | GH18_chitinase | cd06548 | 40-430 | 7.47E-147 |  |
|  |  |  |  | ChiC_BD | cd12215 | 547-590 | 1.94E-14 |  |
|  |  |  |  | FN3 | cd00063 | 457-538 | 1.46E-13 |  |
|  |  |  |  | ChiA | COG3325 | 30-447 | 1.37E-157 |  |
|  |  |  |  | COG3979 | COG3979 | 458-595 | 1.63E-19 |  |
|  | **group C** |  | **G25-74_03105** | GH18_chitinase | cd06548 | 6-328 | 3.01E-114 |  |
|  |  |  |  | Glyco_18 | smart00636 | 5-324 | 6.66E-92 |  |
|  |  |  | G25-126_00586 | GH18_chitinase | cd06548 | 23-330 | 2.93E-121 |  |
|  |  |  |  | Glyco_18 | smart00636 | 9-330 | 1.39E-101 |  |
|  |  |  | G25-117_04734 | GH18_chitinase | cd06548 | 24-330 | 6.26E-121 |  |
|  |  |  |  | Glyco_18 | smart00636 | 19-330 | 2.31E-97 |  |
|  |  |  | G25-118_03195 | GH18_chitinase | cd06548 | 24-330 | 6.26E-121 |  |
|  |  |  |  | Glyco_18 | smart00636 | 19-330 | 2.31E-97 |  |
|  |  |  | G25-126_00743 | GH18_chitinase | cd06548 | 35-363 | 1.68E-91 |  |
|  |  |  |  | Glyco_18 | smart00636 | 33-363 | 1.82E-79 |  |
|  |  |  | G25-117_03754 | GH18_chitinase | cd06548 | 86-364 | 1.61E-86 |  |
|  |  |  |  | Glyco_18 | smart00636 | 55-364 | 2.89E-75 |  |
|  |  |  | G25-118_01075 | GH18_chitinase | cd06548 | 86-364 | 1.61E-86 |  |
|  |  |  |  | Glyco_18 | smart00636 | 55-364 | 2.89E-75 |  |
|  |  | **fungal chitinases** | **Lp_ABQ57240.1** | GH18_chitinase | cd06548 | 40-383 | 2.75E-122 |  |
|  |  |  |  | Glyco_18 | smart00636 | 41-383 | 6.90E-127 |  |
|  |  |  | **Cr_ABV57861.1** | GH18_chitinase | cd06548 | 43-386 | 1.55E-128 |  |
|  |  |  |  | Glyco_18 | smart00636 | 44-386 | 1.11E-124 |  |
|  |  |  | **Tv_AAL78814.1** | GH18_chitinase | cd06548 | 8-352 | 1.27E-132 |  |
|  |  |  |  | Glyco_18 | smart00636 | 7-352 | 1.67E-123 |  |

Note: 1. Protein IDs, such as G25-39_01563 are from nematicidal spore-forming Bacilli. The protein IDs in bold represent sequences of the groups.

2. Domains of proteins, including fungal virulence chitinases are analysed by conserved domain database (CDD) of GenBank.

3. The descriptions of the same domains were omitted for shortening the length of supplementary information.
